# Lung cancer diagnosis from CT scans using artificial intelligence techniques: A global perspective

**DOI:** 10.1016/j.clinsp.2026.100930

**Published:** 2026-04-15

**Authors:** Yuanyuan Wang, Weihong Liu, Yongzhong Cao, Fangxing Chen

**Affiliations:** Department of Radiology, CR&WISCO General Hospital, Wuhan, Hubei, China

**Keywords:** Artificial intelligence, Diagnostic accuracy, Computed tomography, Lung cancer

## Abstract

**Objective:**

In this research, we conducted a systematic review of artificial intelligence techniques used for the diagnosis of lung cancer.

**Methods:**

A systematic search of Web of Science, PubMed, Scopus, Epistemonikos, Cochrane, Medline, and Embase databases was carried out, containing the literature published up to June 2025. Prediction model risk of bias assessment tool (PROBAST) was used to evaluate the risk of bias and applicability of the diagnostic model studies included in the current research.

**Results:**

204 studies were included. The included articles utilized various AI techniques, including CNN (Convolutional Neural Network), SVM (Support Vector Machine), RF (Random Forest), KNN (K-Nearest Neighbor), PM-DL (Pattern Matching combined with Deep Learning), ANN (Artificial Neural Network), DNN (Deep Neural Network), CDNs (Convolutional Dense Networks), DLS (Deep Learning System), LSTM (Long Short-Term Memory), NNE (Neural Network Ensemble), and LDA (Linear Discriminant Analysis). The CNN model appears to be the most commonly used model in the papers. It was observed that applying deep learning models to preprocessed and augmented medical images led to improved performance metrics, including AUC, sensitivity, and accuracy. The accuracy of artificial intelligence techniques ranged from 68.4 to 100, while the sensitivity varied from 50.0 to 100. The specificity of the artificial intelligence techniques ranged from 50.0 to 100. The AUC of the artificial intelligence techniques ranged from 61.0 % to 100 %, while the recall varied from 75.0 to 99.82.

**Conclusion:**

This research accentuates the potential of artificial intelligence techniques in the diagnosis and detection of lung cancer, with diverse levels of diagnostic accuracy. Additional research is required to optimize these artificial intelligence techniques, as well as to ascertain their clinical relevance and appropriateness in real-world clinical applicability.

## Highlights

• Artificial intelligence can be used to detect lung cancer.
• Convolutional Neural Networks (CNN) demonstrated the best performance.
• Deep learning models lead to improved performance metrics.

## Introduction

The worldwide effect of lung cancer is substantial. Lung cancer is one of the leading causes of cancer-related mortality worldwide^[^1, 2, 3, 4, 5 Most cases are diagnosed at an advanced stage of the disease, resulting in a poor prognosis. Early diagnosis and treatment of lung cancer can lessen its critical outcomes. In this regard, lung imaging is frequently conducted in patients with a background of cancer^[^^1^,^3^^,^^6^^–^ Computed Tomography (CT), which is well-known for its non-invasive and versatile nature, is a well-established and reliable imaging technique for detecting lung tumors^[^^1^,^3^^,^^6^^–^ The widespread availability of CT has elevated the incidental detection of internal lesions; however, the clinical significance of these findings often remains unclear. Therefore, it is necessary to undertake follow-up measures such as observation, further diagnostic evaluations, or empirical therapies^[^^1^,^3^^,^^6^^–^

The manual examination of CT scan images by specialists is time-consuming and labor-intensive, requiring detailed inspection of sequential slices in multiple planes to accurately detect lesions^[^^6^,^8^^–^ Furthermore, the classification process is subjective, and heavily dependent on the expertise of medical professionals^[^^6^^,^^7^^,^^9^^,^^10^ Approximately two trillion medical images are captured worldwide each year^[^^6^^,^^7^^,^^9^ However, the number of radiologists is limited, particularly in developing countries^[^^6^^,^^7^^,^^9^ Artificial intelligence techniques can help address this challenge by providing accurate diagnoses and reducing the workload of experts^[^^6^^,^^7^^,^^9^

The expeditious expansion of computing power has enabled the development of more sophisticated algorithms within Artificial Intelligence (AI). The application of these advancements is escalating in the medical field, where a growing number of AI tools assist healthcare professionals in their routine clinical tasks^[^^1^,^3^^,^^6^^–^ Most AI methods utilized in medicine are classified as Machine Learning (ML) algorithms, which can be divided into classical ML algorithms and Deep Learning (DL)^[^^1^,^3^^,^^6^^–^ Machine learning primarily accentuated on analyzing data to create algorithms capable of recognizing behavioral patterns, which has facilitated the development of prognostic models^[^^1^,^3^^,^^6^^–^ Various ML techniques, including Support Vector Machines (SVM), classification and regression trees, and Artificial Neural Networks (ANNs), have been widely applied in medical research^[^^1^,^3^^,^^6^^–^ Over the decades, technological advancements have led to the emergence of deep learning, as an advanced form of ML. This approach involves multi-layered neural network algorithms that utilize methods such as Convolutional Neural Networks (CNN)^[^^1^,^3^^,^^6^^–^

Recent advances in artificial intelligence have considerably promoted the automated analysis of radiographic patterns in medical imaging data^[^^1^,^3^^,^^6^^–^ In oncology, AI has demonstrated its ability to analyze medical images, thereby improving cancer detection and diagnosis^[^^1^,^3^^,^^6^^–^ Advanced AI capabilities can augment the qualitative expertise of specialists^[^^1^,^3^^,^^6^^–^ These include accurately measuring tumor size over time, simultaneously monitoring multiple lesions, translating intratumoral phenotypic particulars into genetic interpretations, and prognosticating patient outcomes by comparing individual tumors to extensive databases containing numerous similar samples^[^^1^,^3^^,^^6^^–^

Numerous techniques have been developed for analyzing CT images of lung cancer. Several studies have reviewed these techniques^[^^1^,^3^^,^^6^^–^ Alina Cornelia Pacurari et al. performed a review on the use of machine and learning artificial intelligence algorithms in the diagnosis and classification of lung cancer, including nine studies in their analysis^[^^3^ Mingsi Liu et al. examined AI and imaging techniques for the automated detection of lung cancer, incorporating fourteen studies^[^^2^ In a systematic review by Mohammed Kanan et al., various AI-driven techniques for lung cancer detection and outcome prediction were investigated, encompassing thirty-nine studies^[^^1^ However, these reviews have only covered a limited range of artificial intelligence techniques for lung cancer detection and diagnosis. Furthermore, due to the inclusion and exclusion criteria applied in these studies, many relevant studies were omitted.

In summary, AI techniques can simplify human activities and reduce certain human errors, particularly in medicine. To this end, numerous techniques have been developed across various fields. However, the large number of these techniques makes it challenging for users to identify the most appropriate one. Consequently, comprehensive research is necessary to thoroughly characterize these methods and evaluate their diagnostic performance. Given the importance of this area, this systematic review aims to examine the AI techniques developed for analyzing CT scan images of lung cancer.

## Methods

### Review protocol

**Fig. 1:**
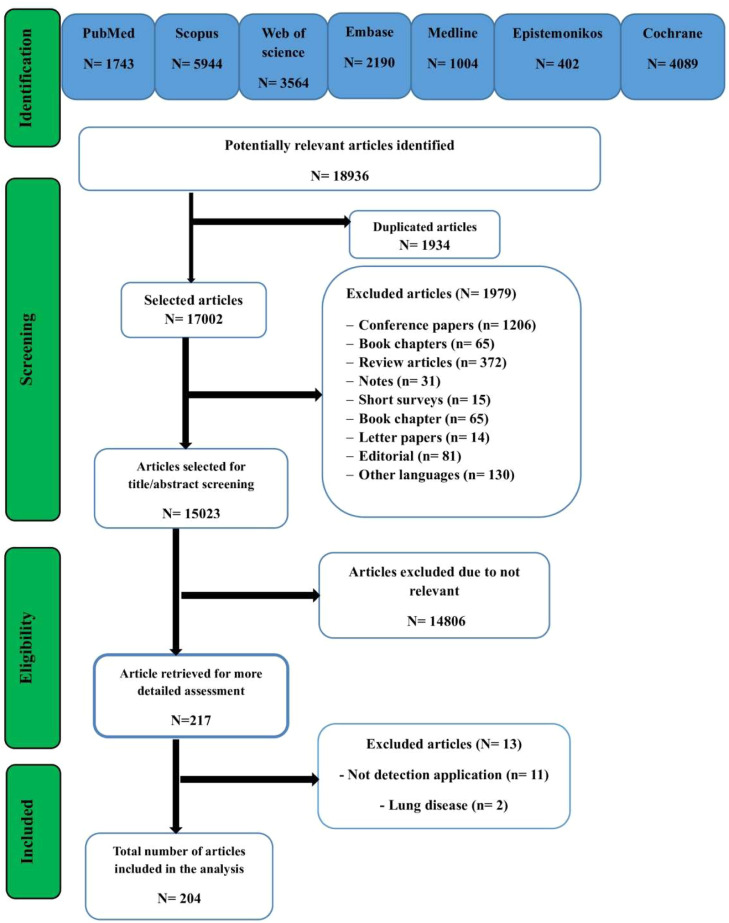
PRISMA flow diagram in the present stud.

This research was registered in the PROSPERO database (CRD420251068938). A comprehensive search was made in accordance with the guidelines presented by the Preferred Reporting Items for Systematic Reviews and Meta-Analyses (PRISMA)^[^^11^ Fig. 1 demonstrates the PRISMA flow diagram in this research.

### Search strategy

A comprehensive search was made across seven electronic bibliographic databases (Web of Science, PubMed, Scopus, Epistemonikos, Cochrane, Medline, and Embase), containing the literature published up to June 2025. This search applied combinations of three sets of keywords identified in the sections of titles, abstracts, and keywords in the papers. The first set of keywords comprised “lung cancer*” OR “lung neoplasm*” OR “pulmonary cancer*” OR “pulmonary neoplasm*”. The second set of keywords was as follows: “AI” OR “artificial intelligence” OR “machine intelligence” OR “computational intelligence” OR “machine learning” OR “deep learning” OR “computer-aided diagnos*” OR “CAD”. The third set of keywords was as follows “CT” OR “computed tomography” OR “computerized tomography”.

The main objective of the current systematic review was to address these research questions:

• How accurate are artificial intelligence techniques in detecting lung cancer?
• What artificial intelligence techniques are currently used?
• What is the clinical significance of the diagnostic imaging techniques?

### Eligibility criteria

The systematic review encompassed studies analyzing artificial intelligence techniques designed for CT scan images of benign or malignant lung nodules and lung cancer diagnosis. The datasets considered included public, clinical cohort, and private sources. The review covered diverse types of investigations published in English. However, it excluded review articles, meta-analyses, editorial letters, conference papers, book chapters, short surveys, notes, and data papers. Additionally, studies focusing on diseases other than lung cancer, using tools other than CT scans, or published in languages other than English were excluded.

### Study selection

All articles retrieved from the databases were imported to Endnote software, where all duplicates were removed. In the next step, two investigators screened the imported papers by scrutinizing the titles and abstracts to specify their relevance and in cases where there was disagreement, agreement was achieved after discussion between the two investigators. Studies with unrelated titles and abstracts were eliminated. The investigator further read the full texts of the remaining studies to select those met the entry criteria. Eventually, appurtenant studies were chosen for final review.

### Quality assessment

Prediction model risk of bias assessment tool (PROBAST) was used to evaluate the risk of bias and applicability of the diagnostic model studies included in the current research. PROBAST was created by a steering committee that reviewed existing Risk of Bias (ROB) tools and reporting standards. It is structured into four key areas: participants, predictors, outcomes, and analysis. Within these areas, there are 20 signaling questions designed to guide a systematic evaluation of ROB, which is defined as bias arising when flaws in study design, execution, or analysis cause consistently skewed estimates of a model’s predictive accuracy. PROBAST provides a focused and transparent method for assessing the risk of bias and relevance of studies that develop, validate, or update prediction models for personalized predictions. While PROBAST was primarily developed for use in systematic reviews, it is also applicable for general critical appraisal of prediction model research^[^^12^^,^^13^

### Data extraction

In this step, relevant information was thoroughly collected from the included studies. The extracted data included the first author's name, year of publication, country, sample size, type and name of the method, type of CT scan, dataset, nature of the dataset, data pre-processing techniques, augmentation and optimization techniques, train/validation/test split methodology, comparison to a baseline, and performance metrics.

### Statistical methods

A meta-analysis was performed, but because of the high heterogeneity and the GRADE evaluation showing very low confidence in the results, the meta-analysis findings were considered unreliable and therefore excluded from the study. The GRADE assessment for this research identified the following issues: serious risk of bias, serious inconsistency, serious indirectness, serious imprecision, and a strong suspicion of publication bias.

## Results

### Search results and study selection

In the current systematic review, 18,936 papers were retrieved from the databases. Among them, 1934 duplicate papers were found and eliminated. Subsequently, the titles and abstracts of the 17,002 remaining papers were scrutinized by investigators. After this initial screening, 15,023 papers were removed after appraisement in accordance with the inclusion and exclusion criteria. Afterwards, the full texts of the 217 remaining papers were scrutinized and 204 studies were ultimately included.

### Specification of the articles

**Fig. 2:**
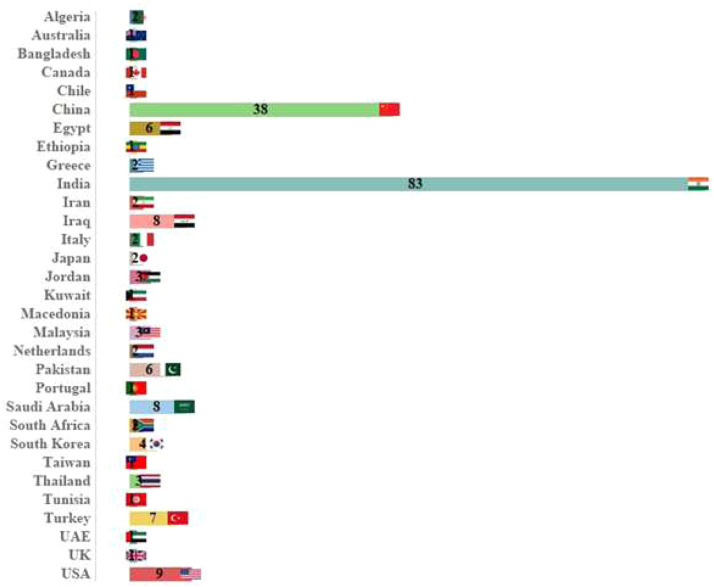
The number of studies conducted on the development of AI techniques for CT scan image analysis of lung cancer in different countries.

**Fig. 3:**
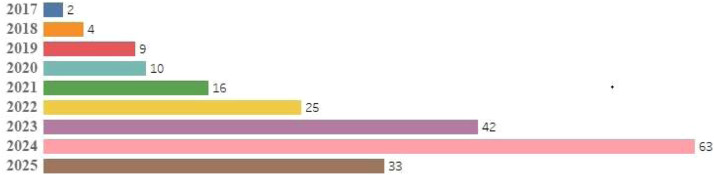
The number of studies conducted on the development of artificial intelligence techniques for CT scan image analysis of lung cancer in the different years.

Fig. 2 illustrates the number of investigations conducted in various countries, while Fig. 3 presents the number of investigations carried out in different years. Additionally, most research articles were published recently, between 2023 and 2025. The majority of investigations were conducted in India (83 papers; 40.68 percent), China (38 papers; 18.62 percent), the United States (9 papers; 4.41 percent), Saudi Arabia (8 papers; 3.92 percent), Iraq (8 papers; 3.92 percent), and Turkey (7 papers; 3.43 percent). Furthermore, most of the studies were carried out in 2024 (63 papers; 30.88 percent), 2023 (42 papers; 20.58 percent), and 2025 (33 papers; 16.17 percent). All the studies included in this research were retrospective.

### Main findings

The comparative analysis section reviewed various studies by different researchers on lung cancer detection using AI techniques. The diagnostic results are evaluated based on parameters such as accuracy, sensitivity/recall, precision, specificity, and Area Under the Curve (AUC). Table 1 presents a summary of the study characteristics for some of the included studies; the full details of all included studies are provided in Tables S1 and S2. The analysis reveals that numerous studies have employed traditional machine learning and deep learning methods for lung cancer diagnosis and detection. It is evident that deep learning approaches generally performed better, achieving higher accuracy in diagnosis and detection results.

**Table 1:** Summary of the study characteristics for some of the included studies.

First author (year)	Country	Accuracy ( %)	Sensitivity ( %)	Specificity ( %)	Precision ( %)	Area under ROC (AUC) ( %)	Recall ( %)	F1- score ( %)	Risk	Concern
Priya A (2024)^6^	India	98.65	98.85	97.91				98.09	Low	Unclear
Bushara A R (2023)^7^	India	94.0		99.07	95.0	98.9	94.5	94.5	Low	Low
Mattakoyya Aharonu (2024)^14^	India	99.92		99.90	99.94		99.94	99.92	Low	Low
Akila Agnes Sundaresan (2018)^15^	India	93.67							High	High
Ahmed Alksas (2023)^16^	USA	96.17	97.14	95.33		98.0			Unclear	Unclear
Firas H Almukhtar (2024)^17^	Iraq	96.67							Unclear	Low
Mohammad H Alshayeji (2023)^18^	Kuwait	99.65	99.64	99.90	99.76				Low	Low
Sajeev Ram Arumugam (2024)^19^	India	93.4			89.8			91.2	High	High
Abolfazl Bagheri Tofighi (2025)^20^	Iran	96.83			96.78		96.83	96.78	Low	Low
Yang Bai (2022)^21^	China	94.6							Unclear	Low
Prasanalakshmi Balaji (2023)^22^	Saudi Arabia				95.75				High	High
Ananya Bhattacharjee (2021)^23^	India	76.0	75.0	78.0	85.71		75.0	79.9	High	High

The accuracy of the artificial intelligence techniques ranged from 68.4 to 100, while the sensitivity varied from 50.0 to 100. The specificity of the artificial intelligence techniques ranged from 50.0 % to 100 %, while the precision varied from 74.7 to 100. The AUC of the artificial intelligence techniques ranged from 61.0 % to 100 %, while the recall varied from 75.0 to 99.82. Finally, the F1-score of the artificial intelligence techniques ranged from 60.9 % to 99.92 %. The remaining information was presented in the Supplementary File.

Data pre-processing techniques varied across studies and included resizing and normalization methods, histogram equalization method, wiener, median, Gaussian, Gabor, bilateral, anisotropic diffusion, Savitzky-Golay, Laplacian, Haar, Sobel, Scharr, and Prewitt filters, as well as the Contrast-Limited Adaptive Histogram Equalization (CLAHE) method.

Several augmentation and optimization techniques were employed in the included studies, such as rescaling, rotation, horizontal and vertical flipping, Social Spider Optimization (SSO), Honey Badger Golden Search Optimization (HBGSO), cuckoo search optimization, particle swarm optimization algorithms, as well as Adam and Stochastic Gradient Descent with Momentum (SGDM) optimizers.

Regarding the train/validation/test split methodology, several approaches were used in the included studies, such as 60 % for training, 20 % for testing, and 20 % for validation, or 75 % for training and 25 % for testing.

Regarding the validation of AI-based models for lung cancer risk detection, studies compared the diagnostic performance of these models with established architectures such as VGG-19, Inception-v3, ResNet-152, and SqueezeNet, as well as with the performance of radiologists.

The results of the PROBAST evaluation are presented in Table S1. Regarding the overall judgment of Risk Of Bias (ROB), fifty-three studies were rated as high ROB, forty-one studies as unclear ROB, and one hundred ten studies as low ROB. Regarding the overall judgement of applicability, fifty-one studies were rated as high concern, fifty-one studies as unclear concern, and one hundred two studies as low concern. The participant and analysis domains were the most common sources of bias.

## Discussion

In this review, we summarize published research that employs AI-based learning methods for diagnosing lung cancer. The study focuses on research involving the identification of benign and malignant lung nodules, as well as the diagnosis of lung cancer using AI-based learning approaches.

AI-based methods have played a major role in advancing cancer research. Most studies reported in the literature have primarily focused on deep learning approaches. Deep learning models have outperformed traditional machine learning models in this field. Among deep learning techniques, Convolutional Neural Networks (CNNs) are the most frequently employed for cancer detection. Additionally, Neural Networks (NN) and Deep Neural Networks (DNN) have been widely utilized. Beyond deep learning, ensemble learning methods such as random forest methods with weighted voting and gradient boosting machines, along with Support Vector Machines (SVM), are also commonly applied in the research. It was observed that applying deep learning models to pre-processed and augmented medical images leads to improved performance metrics, including AUC, sensitivity, and accuracy. Hybrid deep convolutional network in the study of Das et al. (accuracy: 99.92, specificity: 99.90, precision: 99.94, recall: 99.94, F1-score: 99.92),^14^ NoduleDiag in the study of Alshayeji et al. (accuracy: 99.65, sensitivity: 99.64, specificity: 99.90, precision: 99.76),^18^ Internet of Things (IoT)-enabled platform in the study of Chapala et al. (accuracy: 99.59, sensitivity: 99.31) (15), CenterNet in the study of Dawood et al. (precision: 99.89, recall: 99.82, F1-score: 99.85),^24^ federated learning and block chain systems in the study of Heidari et al. (accuracy: 99.69),^25^ ELCD-NSC model in the study of Helaly et al. (accuracy: 99.60, precision: 99.61, Area Under Curve (AUC): 99.75, recall: 99.62, F1-score: 99.70),^23^ DenseNet20, EfficientNetB7, VGG16, MobileNet and VGG19 models in the study of Jain et al. (accuracy: 99.40),^26^ Convolutional Neural Network (CNN) based Long and Short Term Memory (LSTM) in the study of Kanipriya et al. (accuracy: 99.03),^27^ Multi-Head Attention-based fused Depth-wise Convolutional Neural Network (MHA-DCNN) in the study of Kavitha et al. (accuracy: 99.54, precision: 99.7, F1-score: 99.5),^28^ Sooty-LuCaNet in the study of Muthazhagan et al. (accuracy: 99.16),^29^ Squeeze and Excitation convolutional Neural Networks (SENET) in the study of Parvathy et al. (accuracy: 99.2, precision: 99.1),^30^ butterfly optimization algorithm-based k-means clustering (BOAKMC) algorithm in the study of Pethuraj et al. (accuracy: 99.15),^31^ spotted hyena optimization with seagull algorithm in the study of Prasad et al. (accuracy: 99.6, sensitivity: 99.8, specificity: 99.3, precision: 99.14),^32^ federated learning with convolution neural network (ResNet50) in the study of Usharani et al. (accuracy: 99.40),^33^ Unsupervised Anomaly Detection (UAD) in the study of Lee et al. (accuracy: 99.8, sensitivity: 100, specificity: 99.8, precision: 99.8, Area Under Curve (AUC): 100, F1-score: 99.8),^34^ convolutional neural networks + layers with Long Short-Term Memory (CNN+LSTM) in the study of Hammad et al. (accuracy: 99.04, precision: 99.61, recall: 99.23, F1-score: 99.24),^35^ Convolutional Neural Networks & minimum Redundancy Maximum Relevance (CNN & mRMR) in the study of Togaçar et al. (accuracy: 99.51),^36^ attention enhanced inception next-based hybrid deep learning model in the study of Ozdemir et al. (accuracy: 99.54, precision: 99.67, recall: 99.60, F1-score: 99.12),^37^ Xception model in the study of Tawfik et al. (accuracy: 99.03, sensitivity: 99.03, precision: 99.68, F1-score: 99.34),^38^ probabilistic majority voting and optimization techniques in the study of Sünnetci et al. (accuracy: 99.28, sensitivity: 99.25, specificity: 99.91, precision: 99.34, F1-score: 99.28),^39^ segmented 3D tensors and support vector machines in the study of Nisa et al. (accuracy: 99.68),^40^ Convolutional Neural Networks (CNN) model in the study of Çay et al. (accuracy: 99.39, precision: 99.39, recall: 99.39, F1-score: 99.39),^41^ deep neural networks model in the study of Pagadala et al. (accuracy: 99.26, sensitivity: 99.11, specificity: 99.12),^42^ and Dense Net-CNN model in the study of Zhang et al. (accuracy: 99.3, specificity: 99.6, precision: 99.3, recall: 99.3, F1 score: 99.3)^43^ had highest performance.

AI-assisted detection utilizing various algorithms has different detection results. It has been revealed^44^ that conventional shallow learning algorithms have a greater advantage over deep learning algorithms for diseases with small sample sizes. However, for lung nodules due to lung cancer, which is a condition with a large amount of sample data, deep learning algorithms enhance the detection accuracy of lung cancer more than shallow learning algorithms. Various algorithms, particularly radionics and deep learning, have diverse detection capabilities that can not only help identify the benign and malignant nature of lung nodules, but also predict the aggressiveness and prognostication of small cell lung cancer.

Lee et al. developed an Unsupervised Anomaly Detection (UAD) system with high performance. In this study, the technique includes local feature-based UAD network alongside AI model, which can select 3D voxel patches from low-dose CT and diagnoses anomalies. The integration of UAD network and AI model advances the efficacy of this technique by facilitating precise diagnosis of cancerous lesions. A considerable advantage of this approach is its capacity to lessen human errors and its application to large-scale screening processes^[^^34^ CenterNet model in the study of Dawood et al. also showed high performance. In this research, the model includes ResNet-34 and an attention mechanism in the CenterNet framework. This architecture enhanced feature extraction capacities and empowers the model to recognize lung cancer-related patterns in complex backgrounds and fluctuating environmental conditions^[^^24^ Spotted hyena optimization with seagull algorithm in the study of Prasad et al. reported also great performance. The integration of spotted hyena optimization and seagull algorithm improved the effectiveness of the feature selection process. In this study, DCGAN, generative modeling method, was used to conduct data augmentation^[^^32^ Hammad et al. developed a model including Convolutional Neural Networks and Layers with long Short-Term Memory (CNN+LSTM) with high performance in detection of lung cancer. The integration of convolutional neural networks and layers with long short-term memory has several advantages comprising applicability for extensive datasets, advanced feature extraction capacity, improvement of interpretability and accommodation of the various nature of lung cancer^[^^35^ Furthermore, Tawfik et al. developed Xception model with high performance in early detection of lung cancer. In this research, the Contrast Limited Adaptive Histogram Equalization (CLAHE) algorithm is applied to preprocess images and enhance the visual quality. The CutMix technique was used for data augmentation^[^^38^ Dense Net-CNN model in the study of Zhang et al., also, achieved high performance. The integration of Dense Net and convolutional neural network empowers the system to combine information from various sources and increases the validity and accuracy of the model^[^^43^

The type of data utilized to train the diagnostic model greatly influences its performance. The accuracy and diagnostic outcomes depend on the data employed to train the detection model. The majority of research studies examined in this paper have used the LIDC-IDRI dataset, with the LUNA 16 dataset being the second most commonly used.

The findings of this review emphasize the need for a standardized method in developing and validating lung cancer diagnostic models to reduce bias, as participant selection and analysis were identified as the primary sources of bias in AI models. To address this, researchers should employ PROBAST and other quality assessment tools for diagnostic models to ensure that database selection and analysis are conducted with minimal bias. This includes proper management of missing data, evaluation of model performance using discrimination and calibration metrics, and the application of techniques such as bootstrapping and cross-validation to avoid overfitting. Our Risk of Bias (ROB) assessment showed that a significant number of AI models exhibited unclear or high risk of bias. This is partly attributable to the heavy reliance on electronic health record data for validation datasets in AI studies. Utilizing large registries with electronic health records for validation can result in variability in reporting key model variables and incomplete data. Moreover, AI models often demonstrate less transparency in their analytical processes compared to traditional models, leading to unclear handling of missing data and potential biases in diagnostic selection.

According to the results, the AI-based detection system for CT scan imaging has a significant detection accuracy for the diagnosis of lung cancer, which is of great value for lung cancer detection and enables the realization of wider application in the area of clinical diagnosis. Additionally, the findings of the current research are consistent with previous reports,^45^^,^^46^ specifying that the findings of the current research have reference value, artificial intelligence with the assistance of a deep learning technique, can compensate for missed detection because of inexperience or incompetence of physicians and can enhance work efficiency to some extent. Accordingly, the current research suggests that the application of AI in clinical detection of lung cancer should be increased.

In summary, although AI methods demonstrate strong diagnostic capabilities for lung cancer, the current lack of high-quality studies indicates that these AI models are not yet ready for real-world clinical applicability. Future research should focus on clearer reporting of pathological assessments, larger sample sizes, and additional analyses such as diagnostic explanations, failure assessments, and sensitivity evaluations.

This research, also, has certain limitations: the performance of the techniques can be influenced by tools and protocols. In addition, most techniques have limited discrimination capacity for small tumors with low contrast and areas with blurred edges. In addition, comparisons between CT images and identical conditions should be used to compare the methods. Moreover, the high heterogeneity of the included studies may be due to the various sources of study participants, large gaps in sample size between studies, and variable numbers of features extracted by Artificial Intelligence (AI). Moreover, the well-documented issue of over-optimistic performance in AI studies arises from data leakage, inadequate validation, or testing on non-representative datasets.

Although AI-based methods have demonstrated their importance in cancer detection research, researchers still face numerous challenges that need to be addressed.

1 Limited Data Size: A common issue in many studies is the lack of sufficient data to train models. Small sample sizes result in limited training sets, which do not adequately validate the effectiveness of proposed methods. Larger datasets enable better model training.
2 High Dimensionality: Another data-related challenge in cancer research is the presence of a large number of features relative to the number of cases. While various dimensionality reduction techniques exist to tackle this problem, there remains a need for a more universal approach.
3 Class Imbalance Problem: Medical datasets, particularly cancer data, often suffer from uneven class distributions. This imbalance occurs when sample sizes differ significantly between classes, causing classification models to favor the majority class. Most current methods handle binary class imbalance well but struggle with multi-class scenarios.
4 Computational Time: Most studies favor deep learning methods, especially for analyzing medical images in cancer detection. Although deep learning techniques, such as CNN classifiers used in the majority of studies, deliver strong performance, they require substantial computational time and resources.
5 Efficient Feature Selection Techniques: While many studies have achieved impressive diagnostic results, there is still a need for computationally efficient feature selection methods that can reduce data preprocessing efforts while maintaining high accuracy.
6 Model Generalizability: Research should focus more on enhancing model generalizability. Most models are validated using data from a single site, but validating across multiple sites is necessary to improve robustness and applicability.
7 Clinical Implementation: Despite the success of AI models in cancer research, their practical use in clinical settings remains limited. These models require validation in real-world clinical environments to support medical professionals in confirming diagnoses.

## Conclusion

This review aims to provide an overview of various research approaches in AI-driven cancer diagnosis models. Artificial intelligence has proven to be invaluable in healthcare, especially in cancer diagnosis. This paper offers a comprehensive investigation of the most recent advanced methods for cancer diagnosis and detection, including an in-depth review of machine learning and deep learning models used for cancer detection through medical imaging. Our study found that the majority of previous research utilized deep learning methods, particularly Convolutional Neural Networks. We observed that applying deep learning models to pre-processed and augmented medical images leads to improved performance metrics, including AUC, sensitivity, and accuracy. This study emphasizes the challenges encountered by researchers in developing AI-based diagnostic models. In summary, although AI methods demonstrate strong diagnostic capabilities for lung cancer, the current lack of high-quality studies indicates that these AI models are not yet ready for real-world clinical applicability. Future research should focus on clearer reporting of pathological assessments, larger sample sizes, and additional analyses such as diagnostic explanations, failure assessments, and sensitivity evaluations.

## Ethical statement

The authors confirm that they are accountable for all aspects of the work (if applied, including full data access, integrity of the data and the accuracy of the data analysis) in ensuring that questions related to the accuracy or integrity of any part of the work are appropriately investigated and resolved.

## Ethics approval and consent to participate

Not applicable.

## Data availability statement

The datasets generated and/or analyzed during the current study are available from the corresponding author upon reasonable request.

## Consent for publication

Not applicable.

## Permission

Not applicable.

Data availability statement: All data generated or analyzed during this study are included in this published article (and its supplementary information files).

## Authors’ contributions

YW: Conceptualization; Visualization; Supervision; Project administration.

WL: Data curation; Writing-review & editing.

YC: Data curation; Visualization; Writing-review & editing.

FC: Writing-original draft.

## Funding

None declared.

## Declaration of competing interest

The authors declare no conflicts of interest.

## Appendix Supplementary materials

Image, application 1

## Footnotes

Supplementary material associated with this article can be found, in the online version, at doi:10.1016/j.clinsp.2026.100930.

## References

[1] Kanan M., Alharbi H., Alotaibi N. (2024). AI-driven models for diagnosing and predicting outcomes in lung cancer: a systematic review and meta-analysis. Cancers (Basel).

[2] Liu M., Wu J., Wang N. (2023). The value of artificial intelligence in the diagnosis of lung cancer: a systematic review and meta-analysis. PLoS One.

[3] Pacurari A.C., Bhattarai S., Muhammad A. (2023). Diagnostic accuracy of machine learning AI architectures in detection and classification of lung cancer: a systematic review. Diagnostics.

[4] Khoshakhlagh A.H., Askari Majdabadi M., Yazdanirad S., Carlsen L. (2023). Health risk assessment of exposure to benzene, toluene, ethylbenzene, and xylene (BTEX) in a composite manufacturing plant: monte-Carlo simulations. Hum Ecol Risk Assess Int J.

[5] Khoshakhlagh A.H., Ghobakhloo S., Peijnenburg W.J., Gruszecka-Kosowska A., Cicchella D. (2024). To breathe or not to breathe: inhalational exposure to heavy metals and related health risk. Sci Total Environ.

[6] Priya A., Bharathi S. (2024). Performance analysis of lung cancer detection and classification using efficientNet: a deep learning model. Multimed Tools Appl.

[7] AR B., RS V.K., SS K. (2023). LCD-capsule network for the detection and classification of lung cancer on computed tomography images. Multimed Tools Appl.

[8] Khoshakhlagh A.H., Ghasemi M. (2017). Occupational noise exposure and hearing impairment among spinning workers in Iran. Iran Red Crescent Med J.

[9] Aharonu M., Ramasamy L.K. (2025). An intelligent generative adversarial network multistage lung cancer detection and subtypes classification. Int J Mach Learn Cybernet.

[10] Kalteh H.O., Khoshakhlagh A.H., Rahmani N. (2018). Prevalence of musculoskeletal pains and effect of work-related factors among employees on offshore oil and gas installations in Iran. Work.

[11] Khoshakhlagh A.H., Mohammadzadeh M., Ghobakhloo S., Cheng H., Gruszecka-Kosowska A., Knight J. (2024). Health risk assessment from inhalation exposure to indoor formaldehyde: a systematic review and meta-analysis. J Hazard Mater.

[12] Wolff R.F., Moons K.G., Riley R.D. (2019). PROBAST: a tool to assess the risk of bias and applicability of prediction model studies. Ann Int Med.

[13] de Jong Y., Ramspek C.L., Zoccali C., Jager K.J., Dekker F.W., van Diepen M. (2021). Appraising prediction research: a guide and meta-review on bias and applicability assessment using the prediction model Risk of Bias ASsessment Tool (PROBAST). Nephrology.

[14] Aharonu M., Ramasamy L.K. (2024). An intelligent generative adversarial network multistage lung cancer detection and subtypes classification. Int J Mach Learn Cybernet.

[15] Sundaresan A.A. (2018). Automatic lung cancer detection in low-dose lung CTs using transfer learning. J Adv Res Dyn Cont Syst.

[16] Alksas A., Shaffie A., Ghazal M. (2023). A novel higher order appearance texture analysis to diagnose lung cancer based on a modified local ternary pattern. Comput Method Prog Biomed.

[17] Almukhtar F.H. (2024). Lung cancer diagnosis through CT images using principal component analysis (PCA) and error correcting output codes (ECOC). J Control Decision.

[18] Alshayeji M.H., Se Abed (2023). Lung cancer classification and identification framework with automatic nodule segmentation screening using machine learning. Appl Intell.

[19] Arumugam S.R., Ravichandran B., Baskaran D., Annamalai R. (2024). Lung lobe segmentation and lung cancer detection with hybrid optimization enabled deep learning using CT images. J Mech Med Biol.

[20] Bagheri Tofighi A., Ahmadi A., Mosadegh H. (2025). Improving lung cancer detection via MobileNetV2 and stacked-GRU with explainable AI. Int J Inf Tech.

[21] Bai Y., Li D., Duan Q., Chen X. (2022). Analysis of high-resolution reconstruction of medical images based on deep convolutional neural networks in lung cancer diagnostics. Comput Methods Prog Biomed.

[22] Balaji P., Aluvalu R., Sagar K. (2023). Residual attention network based hybrid convolution network model for lung cancer detection. Intell Deci Tech.

[23] Bhattacharjee A., Murugan R., Majumder S., Goel T. (2021). Neural network–based computer-aided lung cancer detection. Res Biomed Eng.

[24] Dawood H., Nawaz M., Ilyas M.U., Nazir T., Javed A. (2025). Attention-guided CenterNet deep learning approach for lung cancer detection. Comput Biol Med.

[25] Heidari A., Javaheri D., Toumaj S., Navimipour N.J., Rezaei M., Unal M. (2023). A new lung cancer detection method based on the chest CT images using Federated Learning and blockchain systems. Artif Intell Med.

[26] Jain R., Singh P., Kaur A. (2024). An ensemble reinforcement learning-assisted deep learning framework for enhanced lung cancer diagnosis. Swarm Evol Comput.

[27] Kanipriya M., Hemalatha C., Sridevi N., SriVidhya S., Shabu S.J. (2022). An improved capuchin search algorithm optimized hybrid CNN-LSTM architecture for malignant lung nodule detection. Biomed Signal Process Control.

[28] Kavitha S., Patnala E., Sangaraju H.R., Bingu R., Adinarayana S., Dhatterwal J.S. (2025). An optimized multi-head attention based fused depthwise convolutional model for lung cancer detection. Expert Syst Appl.

[29] Muthazhagan B., Ravi T., Sooty-LuCaNet Rajinigirinath D. (2023). Sooty tern optimization based deep learning network for lung cancer detection. J Intell Fuzz Syst.

[30] Parvathy C., Jayan J. (2024). Automatic lung cancer detection using computed tomography based on Chan Vese segmentation and SENET. Opt Mem Neur Netw.

[31] Pethuraj M.S., BbM Aboobaider, Salahuddin L.B. (2023). Analyzing CT images for detecting lung cancer by applying the computational intelligence-based optimization techniques. Comput Intell.

[32] Prasad U., Chakravarty S., Mahto G. (2024). Lung cancer detection and classification using deep neural network based on hybrid metaheuristic algorithm. Soft Comput.

[33] Usharani C., Selvapandian A. (2025). FedLRes: enhancing lung cancer detection using federated learning with convolution neural network (ResNet50). Neur Comput Appl.

[34] Lee J.H., Oh S.J., Kim K., Lim C.Y., Choi S.H., Chung M.J. (2025). Improved unsupervised 3D lung lesion detection and localization by fusing global and local features: validation in 3D low-dose computed tomography. Med Image Anal.

[35] Hammad M., ElAffendi M., Asim M., Abd El-Latif A.A., Hashiesh R. (2024). Automated lung cancer detection using novel genetic TPOT feature optimization with deep learning techniques. Res Eng.

[36] Toğaçar M., Ergen B., Cömert Z. (2020). Detection of lung cancer on chest CT images using minimum redundancy maximum relevance feature selection method with convolutional neural networks. Biocybern Biomed Eng.

[37] Ozdemir B., Aslan E., Pacal I. (2025). Attention enhanced inceptionnext based hybrid deep learning model for lung cancer detection. IEEE Access.

[38] Tawfik N., Emara H.M., El-Shafai W., Soliman N.F., Algarni A.D., Abd El-Samie F.E (2024). Enhancing early detection of lung cancer through advanced image processing techniques and deep learning architectures for CT scans. Comp Mater Contin.

[39] Sünnetci K.M., Alkan A. (2022). Lung cancer detection by using probabilistic majority voting and optimization techniques. Int J Imaging Syst Technol.

[40] un Nisa Z., Jaffar A., Bhatti S.M., Butt U.M. (2023). Lung cancer detection using segmented 3D tensors and support Vector machines. Int J Adv Comp Sci Appl.

[41] ÇAY T. (2025). Lung cancer diagnosis with GAN supported deep learning models. Biomed Mater Eng.

[42] Pagadala P.K., Pinapatruni S.L., Chanda R.K., Katakam S., Peri L.S.K., Reddy D.A (2023). Enhancing lung cancer detection from lung CT scan using image processing and deep neural networks. Revue d'Intelligence Artificielle.

[43] Zhang C., Aamir M., Guan Y. (2024). Enhancing lung cancer diagnosis with data fusion and mobile edge computing using DenseNet and CNN. J Cloud Comput.

[44] Sun W., Zheng B., Qian W. (2017). Automatic feature learning using multichannel ROI based on deep structured algorithms for computerized lung cancer diagnosis. Comput Biol Med.

[45] Jacobs C., van Rikxoort E.M., Murphy K., Prokop M., Schaefer-Prokop C.M., van Ginneken B. (2016). Computer-aided detection of pulmonary nodules: a comparative study using the public LIDC/IDRI database. Euro Radiol.

[46] Gu Y., Lu X., Yang L. (2018). Automatic lung nodule detection using a 3D deep convolutional neural network combined with a multi-scale prediction strategy in chest CTs. Comput Biol Med.

