# Supplementary material for: Lung cancer diagnosis from CT scans using artificial intelligence techniques: A global perspective
Source: Clinics (Sao Paulo). 2026 Apr 15;81:100930. doi: 10.1016/j.clinsp.2026.100930 (PMC13094484; doi:10.1016/j.clinsp.2026.100930)
Supplement: Supplementary file 1 [file mmc1.docx]

CLINICS-D-25-01568_Supplementary Material

**Table S1** Detailed information on the performance of methods developed for the detection and diagnosis of lung cancer.

| **First author (year)** | **Country** | **Sample size** | **Type/name of method** | **Type of CT scan** | **Accuracy (%)** | **Sensitivity (%)** | **Specificity (%)** | **Precision (%)** | **Area under ROC (AUC) (%)** | **Recall (%)** | **F1- score (%)** | **Risk** | **Concern** |
| --- | --- | --- | --- | --- | --- | --- | --- | --- | --- | --- | --- | --- | --- |
| Priya A (2024)[1] | India | 48 | EfficientNet | CT | 98.65 | 98.85 | 97.91 |  |  |  | 98.09 | Low | Unclear |
| Bushara A R (2023) [2] | India | ‒ | Capsule neural network (CapsNet) | Low-dose CT | 94.0 |  | 99.07 | 95.0 | 98.9 | 94.5 | 94.5 | Low | Low |
| Mattakoyya Aharonu (2024) [3] | India | ‒ | Deep Convolutional cross max-out kernel graph-based Generative Adversarial Network with Enhanced Prism refraction Search (DCGAN-EPS) | Low-dose CT | 99.92 |  | 99.90 | 99.94 |  | 99.94 | 99.92 | Low | Low |
| Akila Agnes Sundaresan (2018) [4] | India | ‒ | Transfer learning based feed-forward deep neural network | Low-dose CT | 93.67 |  |  |  |  |  |  | High | High |
| Ahmed Alksas (2023) [5] | USA | ‒ | Hyper-tuned stacked generalization-based classification architecture | CT | 96.17 | 97.14 | 95.33 |  | 98.0 |  |  | Unclear | Unclear |
| Firas H Almukhtar (2024) [6] | Iraq | ‒ | Error correcting output codes (ECOC) | CT | 96.67 |  |  |  |  |  |  | Unclear | Low |
| Mohammad H Alshayeji (2023) [7] | Kuwait | ‒ | NoduleDiag | CT | 99.65 | 99.64 | 99.90 | 99.76 |  |  |  | Low | Low |
| Sajeev Ram Arumugam (2024) [8] | India | ‒ | Fractional honey badger golden search optimization algorithm (FHBGSO)-based Shepard convolutional neural networks (ShCNN) | CT | 93.4 |  |  | 89.8 |  |  | 91.2 | High | High |
| Abolfazl Bagheri Tofighi (2025) [9] | Iran | ‒ | MobileNetV2-SGRU | CT | 96.83 |  |  | 96.78 |  | 96.83 | 96.78 | Low | Low |
| Yang Bai (2022) [10] | China | 74 | High-resolution reconstruction model | Spiral CT | 94.6 |  |  |  |  |  |  | Unclear | Low |
| Prasanalakshmi Balaji (2023) [11] | Saudi Arabia | ‒ | Multi serial hybrid convolution based residual attention network (MSHCRAN) | CT |  |  |  | 95.75 |  |  |  | High | High |
| Ananya Bhattacharjee (2021) [12] | India | ‒ | Feed forward neural network | CT | 76.0 | 75.0 | 78.0 | 85.71 |  | 75.0 | 79.9 | High | High |
| Ananya Bhattacharjee (2022) [13] | India | ‒ | Adaptive boost-based grid search optimized random forest (Ada-GridRF) | CT | 97.97 | 100 | 96.0 | 96.08 | 99.8 |  | 98.0 | Low | Unclear |
| A R Bushara (2023) [14] | India | ‒ | Visual geometry group - capsule network (VGG-CapsNet) | CT | 98.61 |  | 99.07 | 99.07 | 98.0 | 98.16 | 98.61 | Low | Low |
| Venkatesh Chapala (2021) [15] | India | ‒ | Internet of Things (IoT)-enabled platform | CT | 99.59 | 99.31 |  |  |  |  |  | Unclear | Unclear |
| Liuyin Chen (2024) [16] | China | 476 | Self-distillation trained multitask dense-attention network (SD-MdaNet) | CT |  |  |  |  | 98.7 |  |  | Unclear | Unclear |
| Hussain Dawood (2025) [17] | UAE | ‒ | CenterNet | CT |  |  |  | 99.89 |  | 99.82 | 99.85 | Unclear | Low |
| S G Devi (2024) [18] | India | ‒ | Advanced machine learning algorithms | CT | 88.0 |  |  |  |  |  |  | High | High |
| Ashit Kumar Dutta (2022) [19] | India | ‒ | Random forest (RF) classifier with Convolutional Neural Network (CNN) | Low-dose CT | 93.25 |  |  |  |  |  | 91.75 | High | High |
| Veerraju Gampala (2024) [20] | India | ‒ | Archimedes flow regime optimization (AFRO) | CT | 95.8 |  |  | 93.6 |  |  | 92.6 | Unclear | Low |
| Nandita Gautam (2024) [21] | India | ‒ | ResNet-152, DenseNet-169, and EfficientNet-B7 models | CT | 97.23 | 98.6 |  |  |  |  |  | Unclear | Unclear |
| Arash Heidari (2023) [22] | Iran | ‒ | Federated learning and block chain systems | CT | 99.69 |  |  |  |  |  |  | Low | Unclear |
| Hadeer A Helaly (2024) [23] | Egypt | ‒ | ELCD-NSC model | CT | 99.60 |  |  | 99.61 | 99.75 | 99.62 | 99.70 | Low | Low |
| Shengchao Hou (2022) [24] | USA | ‒ | U-Net-Newton’s method | CT | 98.57 | 92.1 | 77.6 |  |  |  |  | Low | Low |
| Ratishchandra Huidrom (2022) [25] | India | ‒ | Neuro-evolutional based computer aided detection system | CT | 95.5 | 95.8 | 95.3 |  |  |  |  | Low | Low |
| Richa Jain (2024) [26] | India | ‒ | DenseNet20, EfficientNetB7, VGG16, MobileNet and VGG19 models | CT | 99.40 |  |  |  |  |  |  | Unclear | Unclear |
| M Kanipriya (2022) [27] | India | ‒ | Convolutional neural network (CNN) based long and short-term memory (LSTM) | CT | 99.03 |  |  |  |  |  |  | Low | Unclear |
| Mehr Kashyap (2025) [28] | USA | ‒ | Three-dimensional U-Net ensemble approach | CT |  | 92.0 | 82.0 |  |  |  |  | Low | Low |
| Sadam Kavitha (2025) [29] | India | ‒ | Multi-head attention-based fused depth wise convolutional neural network (MHA-DCNN) | CT | 99.54 |  |  | 99.7 |  |  | 99.5 | Low | Low |
| Suhad Jasim Khalefa (2024) [30] | Iraq | ‒ | Finite element method- deep auto encoder algorithm-convolutional neural network (FEM-DCNN) | CT | 98.0 |  |  | 97.0 |  | 98.0 | 99.0 | Unclear | High |
| Geethu Lakshmi G (2025) [31] | India | ‒ | Squeeze-Inception-ResNeXt model | CT | 97.7 | 98.1 | 97.4 |  |  |  |  | Low | Low |
| Madhusudan G Lanjewar (2023) [32] | India | ‒ | DenseNet | CT | 95.0 |  |  |  |  |  |  | Low | Unclear |
| Zhengrong J Liang (2025) [33] | USA | ‒ | Machine Learning (ML) models | Low-dose CT |  |  |  |  | 98.0 |  |  | Unclear | High |
| Lei Ma (2024) [34] | China | ‒ | GoogLeNet with Adaptive Layers (GoogLeNet-AL) | CT | 98.74 |  |  | 99.74 |  |  | 98.96 | Low | Low |
| Selma Mammeri (2024) [35] | Algeria | ‒ | YOLO v7 | CT |  |  |  | 81.28 |  |  |  | Low | Unclear |
| Suguna Mariappan (2024) [36] | India | ‒ | Hybrid efficient-ShuffleNet (HES-Net) method | CT | 91.3 |  |  |  |  |  |  | High | High |
| B Muthazhagan (2023) [37] | India | ‒ | Sooty-LuCaNet | CT | 99.16 |  |  |  |  |  |  | Unclear | Unclear |
| Potti Nagaraja (2023) [38] | India | ‒ | Ensemble-based deep learning model (EDLM) | CT | 96.6 | 96.56 | 96.99 | 96.68 |  |  | 96.62 | High | Unclear |
| M Navaneethakrishnan (2023) [39] | India | ‒ | Bat deer hunting optimization algorithm-based deep convolutional neural network (BDHOA-based DCNN) | CT | 92.43 | 94.21 | 89.15 |  |  |  |  | Low | Low |
| Neslihan Ozcelik (2024) [40] | Turkey | 40 | CNNs model | CT | 97.4 | 97.5 | 97.3 |  |  |  |  | High | Unclear |
| Yong-Jin Park (2021) [41] | South Korea | ‒ | Deep transfer learning (TL) models | CT |  |  |  |  | 87.7 |  |  | Unclear | Low |
| C S Parvathy (2024) [42] | India | ‒ | Squeeze and excitation convolutional neural networks (SENET) | CT | 99.2 |  |  | 99.1 |  |  |  | Unclear | Unclear |
| Mohamed Shakeel Pethuraj (2023) [43] | Malaysia | ‒ | Butterfly optimization algorithm-based k-means clustering (BOAKMC) algorithm | CT | 99.15 |  |  |  |  |  |  | Low | Unclear |
| Umesh Prasad (2024) [44] | India | ‒ | Spotted hyena optimization with seagull algorithm | CT | 99.6 | 99.8 | 99.3 | 99.14 |  |  |  | Low | Low |
| Syeda Reeha Quasar (2024) [45] | India | ‒ | BEiT, DenseNet, AND, OR, weighted box fusion, and boosting models | CT | 98.0 |  |  |  |  |  |  | Low | Unclear |
| N Rathan (2024) [46] | India | ‒ | High-resolution net (HRNet) | CT | 98.4 |  |  |  |  |  |  | Low | Low |
| Amjad Rehman (2023) [47] | Saudi Arabia | ‒ | Convolution neural network (CNN) algorithm | CT | 98.33 |  |  |  |  |  |  | Low | Unclear |
| Mohammad Hosein Sabzalian (2023) [48] | Chile | 110 | Bidirectional recurrent neural network (BRNN) | CT | 93.46 |  | 92.18 | 94.15 |  | 92.65 | 93.26 | Low | Low |
| Nishat Shaikh (2023) [49] | India | ‒ | Modified dimension range-based cat swarm optimization (MDR-CSO) | CT | 95.0 |  |  |  |  |  |  | Unclear | High |
| P Mohamed Shakeel (2022) [50] | Malaysia | 48 | Spiral optimization intelligent-generalized rough set approach | CT | 96.2 |  | 98.4 | 97.4 |  | 98.0 | 98.4 | Low | Low |
| Malayil Shanid (2021) [51] | India | ‒ | Adaptive-SALP-elephant herding optimisation algorithm (adaptive-SEOA) | CT | 96.09 |  |  |  |  |  |  | Unclear | Unclear |
| Zhiqiang Shen (2023) [52] | China | ‒ | Weakly-supervised lung cancer detection and diagnosis network (WS-LungNet) | CT |  |  |  |  | 88.63 |  |  | Unclear | Low |
| Feng Shi (2021) [53] | China | ‒ | Semi-supervised deep transfer learning (SDTL) | CT | 88.3 |  |  |  | 91.0 |  |  | Unclear | Low |
| Ebtasam Ahmad Siddiqui (2023) [54] | India | ‒ | Enhanced deep belief network (E-DBN) | CT | 99.42 | 98.49 | 98.31 |  |  |  | 99.37 | Low | Low |
| Amit Singh (2024) [55] | India | ‒ | VGG16 | CT | 97.8 |  |  |  |  |  |  | Low | Unclear |
| Prasath Sivasankaran, (2024) [56] | India | ‒ | Deep belief networks (DBN) stacked Denotion Autoencoders (SDAE) | CT | 80.08 |  |  |  |  |  |  | High | High |
| S Sridevi (2024) [57] | India | ‒ | 3D Trans-DenseUnet++ with Novel Loss Function (3D-TD ++-NLF) | CT | 92.92 |  |  | 93.0 |  |  |  | Low | Low |
| R Sudha (2024) [58] | India | ‒ | Hybrid particle snake swarm optimization (PS2OA) | CT | 97.67 |  |  |  |  |  |  | Unclear | Low |
| R Sudha (2025) [59] | India | ‒ | Adaptive pelican optimization with optimized mask region-based convolutional neural network (R-CNN) | CT | 97.67 |  |  | 95.7 |  | 99.0 | 95.67 | Low | Low |
| C Usharani (2025) [60] | India | 822 | Federated learning with convolution neural network (ResNet50) | CT | 99.40 |  |  |  |  |  |  | Unclear | Low |
| Lakshmana Rao Vadala (2025) [61] | India | ‒ | SpiLenet | CT | 92.10 |  |  | 91.10 |  |  | 90.40 | Low | Low |
| N Velmurugan (2025) [62] | India | ‒ | Rat swarm political optimizer (RSPO) based shepard convolutional neural networks (ShCNN) (RSPO_ShCNN) | CT | 94.8 |  |  | 92.6 |  |  | 93.7 | Low | Low |
| C Venkatesh (2022) [63] | India | ‒ | Dynamic optimization and deep learning technique | CT | 98.0 |  |  |  |  |  |  | Unclear | Unclear |
| Guangxi Wang (2022) [64] | China | 1036 | Lung cancer artificial intelligence detector | Low-dose CT |  | 90.0 | 92.0 |  |  |  |  | Low | Low |
| Meng Wang (2025) [65] | China | ‒ | 3D auto-encoder networks | CT | 93.4 | 90.2 |  |  | 94.1 |  |  | Unclear | Low |
| Niyaz Ahmad Wani (2024) [66] | India | ‒ | DeepXplainer | CT | 97.43 | 98.71 |  |  |  |  | 98.08 | Low | Low |
| Yifei Xu (2022) [67] | China | ‒ | YOLOv3 network | CT | 93.82 | 94.85 | 92.59 |  |  |  |  | Low | Low |
| Yeguo Xu (2022) [68] | China | ‒ | Alexnet optimized by modified Bowerbird optimization algorithm | CT |  |  |  |  |  | 98.06 |  | Low | Unclear |
| Chaohua Yan (2023) [69] | China | 110 | CNN model optimized by Snake optimization algorithm | CT | 96.58 | 95.38 | 94.08 | 84.16 |  |  | 91.53 | Low | Low |
| Runhuang Yang (2024) [70] | China | ‒ | Multimodal integrated feature neural network (MIFNN) | CT |  |  |  |  | 86.65 |  |  | Unclear | Low |
| Sufyan Othman Zaben (2024) [71] | Iraq | ‒ | Convolution neural networks (CNN) model | CT | 96.0 |  |  | 94.0 | 96.0 | 90.0 | 76.0 | Low | Low |
| Qinghe Zheng (2025) [72] | China | ‒ | Reconstruction error based implicit regularization method (REbIRM) | Low-dose CT | 90.06 |  |  |  | 92.77 |  | 81.04 | Low | Low |
| A Angel Mary (2024) [73] | India | 1010 | Novel deep learning-based lung cancer detection (DL-LCD) model | Low-dose CT | 97.88 |  |  | 94.94 |  | 97.63 | 96.42 | High | High |
| Shalini Wankhade (2023) [74] | India | 888 | Cancer cell detection using hybrid neural network (CCDCHNN) | CT | 88.9 | 89.8 | 89.2 | 90.0 |  |  |  | Low | Low |
| Juyoung Lee (2023) [75] | South Korea | 168 | IDCal model | CT |  | 97.0 |  |  |  |  |  | Low | Unclear |
| Ju Hwan Lee (2025) [76] | South Korea | 900 | Unsupervised anomaly detection (UAD) | Low-dose CT | 99.8 | 100 | 99.8 | 99.8 | 100 |  | 99.8 | Low | Low |
| Anum Masood (2018) [77] | China | 18 | DFCNet | Low-dose CT | 86.02 | 80.91 | 83.22 |  |  |  |  | Low | Low |
| Yunzeng Zhang (2025) [78] | China | 210 | Multi-omics model | Low-dose CT |  |  |  |  | 82.3 |  |  | Low | Unclear |
| Jayapradha J (2025) [79] | India | 140 | Enhanced Integrated model for Lung Tumor Identification (EIM-LTI) | CT | 98.52 | 99.03 | 97.44 | 97.14 |  |  | 98.08 | Low | Low |
| Mohamed Hammad (2024) [80] | Saudi Arabia | ‒ | Convolutional neural networks+ layers with long short-term memory (CNN+LSTM) | CT | 99.04 |  |  | 99.61 |  | 99.23 | 99.24 | Low | Low |
| R. Pandian (2022) [81] | India | ‒ | CNN and Google Net deep learning algorithms | CT | 98.0 | 99.0 | 99.0 |  |  |  | 98.0 | High | High |
| Mohammad Q Shatnawi (2025) [82] | Jordan | ‒ | Enhanced CNN model | CT | 100 |  |  | 99.2 |  | 98.0 | 98.4 | High | Unclear |
| A A Abe (2025) [83] | South Africa | 110 | DeepNodule-Detect | CT | 98.17 | 98.21 | 98.13 |  |  |  |  | High | High |
| Michail Tsivgoulis (2022) [84] | Greece | ‒ | SqueezeNet model | CT | 94.3 | 91.3 | 95.3 |  |  |  |  | High | High |
| Negar Maleki (2023) [85] | USA | ‒ | Gradient Boosting (GB), Random Forest (RF), Support Vector Machine (SVM) models | CT | 80.0 |  |  | 74.7 |  | 79.7 | 75.7 | Unclear | Low |
| Masahiro Yanagawa (2020) [86] | Japan | 285 | Three-dimensional convolutional neural network (3D-CNN) | CT | 73.67 | 85.44 | 60.34 |  | 73.0 |  |  | Low | Low |
| Mohammad Alamgeer (2022) [87] | Saudi Arabia | ‒ | DLCADLC-BCT technique | CT | 99.33 | 97.22 | 93.34 | 94.08 |  |  |  | Low | Low |
| Luis Gonçalves (2018) [88] | Portugal | 1010 | CAD system | CT | 68.4 | 55.0 | 87.5 |  | 90.5 |  |  | Unclear | Low |
| Mesut Togaçar (2019) [89] | Turkey | 69 | Convolutional neural networks & minimum redundancy maximum relevance (CNN & mRMR) | CT | 99.51 |  |  |  |  |  |  | Unclear | Unclear |
| Kaiqiang Yang (2020) [90] | China | 997 | U-Net integrated with capsule networks (CapNets) | CT |  |  |  |  | 84.0 |  |  | Unclear | Low |
| Ayomide Abe (2025) [91] | South Africa | 110 | AlexNet, ResNet-50, GoogLeNet models | CT | 95.56 | 99.37 |  |  | 99.66 |  |  | High | High |
| Sreedar Bhukya (2025) [92] | India | ‒ | Learning-based method for lung cancer detection (LbM-LCD) | CT | 98.34 |  |  | 97.98 |  | 93.86 | 95.92 | High | High |
| Jenita Subash (2023) [93] | India | 110 | Oppositional based chimp optimization algorithm (OChOA) ‒ recurrent neural network (RNN) | CT | 97.13 | 95.88 | 97.69 |  |  |  |  | High | High |
| Krmanj F (2024) [94] | Iraq | ‒ | Mask-RCNN | CT | 96.59 | 95.0 | 95.0 |  |  |  | 99.65 | High | High |
| Chitra Thangavel (2023) [95] | India | ‒ | Decked dragonfly optimization-speculative deceptive network (DDO-SDN) | Low-dose CT | 99.25 | 98.7 | 98.9 | 99.2 |  | 98.9 | 98.7 | High | Unclear |
| J Maruthi Nagendra Prasad (2022) [96] | India | 1018 | Fuzzy K-means algorithm | CT | 96.0 | 99.0 | 100 |  |  |  |  | Unclear | Low |
| Abdalbasit Mohammed Qadir (2024) [97] | Iraq | 110 | Hybrid lung cancer stage classifier and diagnosis model (hybrid-LCSCDM) | CT | 98.54 |  |  | 98.63 |  | 96.35 | 97.36 | High | High |
| Bassant Mostafa (2024) [98] | Egypt | 110 | Convolutional neural networks -bi-directional long short-term memory (CNN-Bi LSTM) model | CT | 99.2 |  |  | 97.0 |  | 95.0 | 99.0 | High | Unclear |
| Onur Ozdemir (2019) [99] | USA | 2101 | 3D probabilistic deep learning system | Low-dose CT |  |  |  |  | 87.0 |  |  | Unclear | Low |
| Nabila Elloumi (2023) [100] | Tunisia | ‒ | U-NET convolution network architecture | CT | 98.9 | 97.99 |  |  |  |  |  | High | High |
| Shanchen Pang (2019) [101] | China | ‒ | Densely connected convolutional networks and adaptive boosting | CT | 89.85 |  |  |  |  |  |  | Unclear | Unclear |
| Liu chenyang (2020) [102] | China | ‒ | Joint nodule segmentation/recognition (JNSC) network | CT | 90.82 | 88.79 | 91.78 |  |  |  |  | Low | Low |
| Bhavani Krishna (2024) [103] | India | ‒ | Mask region-based convolutional neural network (Mask-RCNN) | CT | 99.32 |  |  | 98.77 |  | 96.62 | 97.23 | High | High |
| Shuyin Duan (2020) [104] | China | 842 | Artificial neural network (ANN-3) | CT | 90.91 | 88.24 | 93.75 |  | 91.0 |  |  | High | Unclear |
| Shalini Chowdary (2024) [105] | India | ‒ | Transfer operator-based archimedes optimization -advanced dilated ensemble convolutional neural networks (MTO-AO- ADECNN) | Low-dose CT | 97.39 | 97.36 | 97.42 | 97.30 |  |  | 97.33 | Low | Unclear |
| Goran Jakimovski (2019) [106] | Macedonia | 95 | Double convolutional deep neural network (CDNN) | CT | 99.62 | 99.91 | 98.66 |  |  |  |  | Low | Low |
| Burhanettin Ozdemir (2025) [107] | Saudi Arabia | 110 | Attention enhanced inception next-based hybrid deep learning model | CT | 99.54 |  |  | 99.67 |  | 99.60 | 99.12 | Low | Low |
| Yossra Hussain Ali (2023) [108] | Iraq | 19 | Particle swarm optimization (PSO) algorithm | 3D CT | 98.45 | 98.45 | 98.62 | 98.89 |  |  | 98.85 | Low | Low |
| Sotiris Raptis (2024) [109] | Greece | ‒ | DenseNet-201 CNN, XGBoost models | CT | 91.05 | 90.0 | 91.85 |  | 92.0 |  |  | High | Unclear |
| Ailing Liu (2020) [110] | China | 875 | Radiomics nomogram model | CT |  |  |  |  | 80.9 |  |  | Unclear | Low |
| Imran Shafi (2022) [111] | Pakistan | ‒ | Deep learning-assisted SVM-based model | CT | 94.0 |  |  | 95.0 |  | 94.5 | 94.5 | Low | Low |
| Martina Sollini (2023) [112] | Italy | ‒ | Computer-aided diagnosis (CADx) module | Low-dose CT | 80.0 | 85.7 | 73.0 |  |  |  |  | Low | Low |
| Mohammad Alamgeer (2023) [113] | Saudi Arabia | ‒ | Dung beetle optimization modified deep feature fusion model for lung cancer detection and classification (DBOMDFF-LCC) technique | CT | 99.17 | 98.72 | 99.37 | 98.81 |  |  |  | Low | Low |
| Anum Masood (2019) [114] | China | ‒ | Cloud-based 3D deep convolutional neural network (3DDCNN) | CT | 98.51 | 98.4 | 92.0 |  | 96.0 |  |  | Low | Low |
| Ling Zhang (2023) [115] | China | 104 | Radiomics model | CT | 76.6 | 85.7 | 73.9 |  | 83.0 |  |  | Low | Unclear |
| Guanghui Song (2024) [116] | China | ‒ | Two-dimensional sequence detection model based on a ConvLSTM (Convolutional Long Short-Term Memory) network and U-shaped structure network | CT | 87.80 |  |  |  |  |  |  | Low | Unclear |
| Mohd Munazzer Ansari (2025) [117] | China | ‒ | SVMVGGNet-16 | CT | 96.72 |  |  | 87.40 | 96.87 | 84.67 | 85.73 | Low | Unclear |
| Mohammad A Alzubaidi (2021) [118] | Jordan | 1000 | Support vector machine -histogram of oriented gradients (SVM-HOG) | CT | 97.0 | 96.0 | 97.0 |  |  |  |  | Unclear | Low |
| Xiao Zhang (2024) [119] | China | 1088 | Deep convolutional neural network (DCNN) model | CT | 92.25 |  |  |  | 98.12 | 91.54 |  | High | High |
| Nevin Aydın (2021) [120] | Turkey | 301 | Deep CNN methods | CT |  | 93.0 |  | 82.0 |  |  | 87.0 | Unclear | Unclear |
| Vinit Kumar Gunjan (2022) [121] | India | ‒ | Grey wolf optimization algorithm and recurrent neural network (GWO + RNN) | CT | 99.63 | 97.21 | 98.45 | 98.39 |  |  |  | Low | Low |
| Worku J Sori (2021) [122] | Ethiopia | ‒ | Two-path convolutional neural network (DFD-Net) | CT | 87.8 |  | 89.0 |  |  | 87.3 |  | Low | Low |
| Hye Soo Cho (2025) [123] | South Korea | 878 | Artificial intelligence system | CT |  |  |  |  | 91.1 |  |  | Low | Unclear |
| Nitha V R (2023) [124] | India | 110 | ExtRanFS | CT | 99.09 | 98.33 |  | 98.33 |  |  | 98.33 | Low | Low |
| Chia Chi Hsiao (2023) [125] | Taiwan | 241 | Machine learning-based method | Low-dose CT | 95.96 | 96.19 |  | 96.33 | 98.55 |  | 96.19 | Low | Low |
| Isha Bhatia (2024) [126] | India | ‒ | Lightweight advanced deep neural network (DNN) model | CT | 98.2 |  |  | 98.2 |  | 98.2 |  | Low | Unclear |
| Amira Bouamrane (2024) [127] | Algeria | ‒ | Gradient-weighted class activation mapping (Grad-CAM) | CT | 99.38 | 98.76 | 100 | 100 | 100 |  | 99.37 | Low | Low |
| Rajeev Dixit (2025) [128] | India | ‒ | Attention + Cross-Average Pooling | CT | 94.26 |  |  | 93.78 |  | 92.54 | 93.15 | High | High |
| Rana M Mahmoud (2024) [129] | Egypt | 1,018 | MobileNet model | CT | 93.3 |  |  |  |  |  |  | High | High |
| Daisuke Yamada (2024) [130] | Japan | 62 | Multimodal hybrid model | Low-dose CT |  |  |  |  | 90.0 |  |  | Low | Low |
| Kavitha Belegere Chandraiah (2024) [131] | India | ‒ | Convolutional neural network model | CT | 98.44 |  |  | 98.40 | 99.70 | 98.40 | 98.30 | High | High |
| Sneha S Nair (2024) [132] | India | ‒ | Artificial neural network (ANN) model | CT | 96.25 | 98.38 | 68.42 |  |  |  |  | Low | High |
| Venkata Tulasiramu Ponnada (2019) [133] | India | ‒ | efficient CNN (EFFI-CNN) | CT | 87.02 |  |  | 81.0 |  | 98.0 |  | High | High |
| Aswathy S U (2022) [134] | USA | 290 | Bag of visual words-convolutional recurrent neural network model (BoVW–CRNN) | CT | 99.35 | 97.0 | 99.0 | 96.5 |  |  | 95.5 | Low | Low |
| Alaa Wehbe (2024) [135] | Italy | ‒ | YOLOv8 small model | CT |  |  |  | 96.1 |  | 94.5 | 95.2 | Low | Low |
| Nahed Tawfik (2024) [136] | Egypt | ‒ | Xception model | CT | 99.03 | 99.03 |  | 99.68 |  |  | 99.34 | Low | Low |
| Suseela Triveni Vemula (2024) [137] | India | 69 | Convolutional neural network (CNN) model | CT | 91.34 | 91.67 |  |  |  | 92.67 | 92.0 | High | High |
| Sayana Sharma (2019) [138] | India | ‒ | Convolutional neural network (CNN) model | CT | 98.08 | 90.39 | 96.6 |  |  |  |  | Low | Low |
| Ananya Bhattacharjee (2023) [139] | India | 110 | Modified Xception model | CT | 99.39 |  |  | 99.33 |  | 98.0 | 98.67 | Low | Low |
| Shaik Karimullah (2024) [140] | India | ‒ | Colliding bodies optimization + densely connected CNN (CBO + DenseNet CNN) | CT | 98.17 |  | 97.32 | 97.46 |  | 97.89 |  | Low | Low |
| Chapala Venkatesh (2022) [141] | India | ‒ | Cuckoo search optimization + convolutional neural network + local binary pattern (CSO+CNN+LBP) | CT | 96.97 | 97.80 | 92.67 |  |  |  |  | Low | Low |
| Sugandha Saxena (2024) [142] | India | ‒ | Maximum sensitivity neural network (MSNN) model | CT | 96.9 | 94.6 | 100 | 100 |  |  | 97.22 | High | High |
| Shanchen Pang (2020) [143] | China | 125 | VGG16-T | CT | 86.58 | 86.0 | 72.63 |  |  |  |  | Low | Unclear |
| Sunil Kumar (2025) [144] | India | ‒ | Residual U-Net + ResNet-101 | CT | 98.01 |  |  | 90.39 |  | 97.37 | 93.71 | High | High |
| N Raghapriya (2024) [145] | India | ‒ | Convolutional neural network- long short-term memory (CNN-LSTM) model | CT | 96.35 | 96.5 | 96.0 | 97.0 |  | 96.5 | 97.0 | High | Unclear |
| Saurabh Singh Raghuvanshi (2024) [146] | India | 1000 | Particle swarm optimization and bayesian optimization (PSbBO-Net) | CT | 98.8 |  |  | 97.4 |  | 98.3 | 98.6 | High | Unclear |
| Yifan Wang (2024) [147] | USA | 1951 | Radiomics-based reinforcement learning (RRL) model | Low-dose CT |  |  |  |  | 85.0 |  |  | Low | Unclear |
| Lihaoyun Huang (2025) [148] | China | 610 | Generative artificial intelligence (Gen-AI) models | CT | 82.97 |  |  |  | 61.0 |  |  | Low | Low |
| Muayed S AL Huseiny (2021) [149] | Iraq | 110 | Transfer learning with GoogLeNet | CT | 94.38 | 95.08 | 93.7 |  |  |  |  | High | High |
| Laxmikant Tiwari (2021) [150] | India | ‒ | Target based weighted Elman DL neural network (TWEDLNN) model | CT | 96.0 | 94.0 | 97.0 |  |  |  |  | Low | Low |
| Qingji Tian (2021) [151] | China | 61 | Enhanced capsule networks- converged search and rescue (ECN-CSAR) model | CT | 96.65 |  |  | 96.35 |  | 96.07 | 96.41 | Low | Low |
| Malayil Shanid (2020) [152] | India | ‒ | SALP-elephant herding optimization algorithm-based deep belief network (SEOA-DBN) model | CT | 96.0 |  |  |  |  |  |  | High | High |
| Muhammad Asim Saleem (2023) [153] | Thailand | ‒ | Sooty tern optimization (SHOA)-optimized DNN model | CT | 91.67 |  | 98.38 | 88.44 |  | 87.89 | 87.93 | Low | Low |
| Rakesh Sankaran (2024) [154] | India | ‒ | Probabilistic bilateral convolutional neural networks (PB-CNN) models | CT | 85.0 |  |  | 93.60 |  | 85.45 | 90.0 | High | High |
| Yu Ding (2023) [155] | China | 424 | Artificial intelligence diagnosis model | CT |  | 82.29 | 90.48 |  | 89.9 |  |  | Low | Low |
| Rapeepat Klangbunrueang (2024) [156] | Thailand | 110 | VGG16 model | CT | 98.18 |  |  |  |  |  |  | Low | Unclear |
| Venkata Tulasiramu Ponnada (2019) [157] | India | ‒ | Edge AI system | CT | 85.23 |  |  | 78.0 |  | 97.0 |  | High | High |
| Colin Jacobs (2021) [158] | Netherlands | 300 | Deep learning algorithms | Low-dose CT |  |  |  |  | 89.3 |  |  | Low | Low |
| Nasr Y Gharaibeh (2024) [159] | Jordan | ‒ | Convolutional neural network- probabilistic neural network (SCNN- PNN) | 3D CT | 97.5 | 99.5 |  | 95.5 |  |  |  | Low | Unclear |
| Mohammad Javad Shafiee (2017) [160] | Canada | 93 | Evolved deep radiomic sequencer (EDRS) | CT | 88.78 | 93.42 | 82.39 |  |  |  |  | Low | Low |
| Wang Du (2022) [161] | China | 152 | Artificial intelligence (AI) model | CT | 89.69 | 92.98 | 65.22 |  |  |  |  | High | High |
| Imran Nazir (2023) [162] | Pakistan | 61 | Machine learning (ML) model | CT | 98.2 | 96.4 | 97.2 |  |  |  |  | Unclear | High |
| Mahir Afser Pavel (2024) [163] | Bangladesh | 422 | Knowledge distillation approach with teaching assistant | CT | 94.53 |  |  |  |  |  | 94.65 | Low | Unclear |
| S Lalitha (2021) [164] | India | ‒ | Machine learning algorithm | CT | 98.7 | 97.9 | 97.2 |  |  |  |  | Low | Unclear |
| Wafaa Alakwaa (2017) [165] | Egypt | 1397 | 3D convolutional neural network (3D-CNN) | CT | 86.6 |  |  |  |  |  |  | High | High |
| P Mohamed Shakeel (2019) [166] | Malaysia | ‒ | Deep learning with instantaneously trained neural networks (DITNN) | CT | 98.42 |  |  | 98.8 |  | 98.26 | 98.64 | Low | Low |
| Suren Makaju (2018) [167] | Australia | 1018 | Computer aided diagnosis (CAD) model | CT | 92.0 | 100 | 50.0 |  |  |  |  | High | High |
| Annemneedi Lakshmanarao (2025) [168] | India | ‒ | Machine learning (ML) algorithms | CT | 89.0 |  |  | 87.84 |  | 88.34 | 86.34 | High | Unclear |
| Chamak Saha (2025) [169] | USA | ‒ | Lung-AttNet | CT | 91.5 |  |  | 91.69 | 98.94 | 91.5 | 91.48 | Low | Low |
| Kubilay Muhammed Sünnetci (2022) [170] | Turkey | ‒ | Probabilistic majority voting and optimization techniques | CT | 99.28 | 99.25 | 99.91 | 99.34 |  |  | 99.28 | Low | Low |
| Zaib un Nisa (2023) [171] | Pakistan | 888 | Segmented 3D tensors and support vector machines | CT | 99.68 |  |  |  |  |  |  | High | High |
| Talip Çay (2025) [172] | Turkey | ‒ | Convolutional neural networks (CNN) model | CT | 99.39 |  |  | 99.39 |  | 99.39 | 99.39 | High | Unclear |
| Rabbia Mahum (2023) [173] | Pakistan | ‒ | Lung-RetinaNet | CT | 99.8 |  |  | 99.4 | 98.9 | 99.3 | 99.5 | Low | Low |
| S Vishwa Kiran (2022) [174] | India | ‒ | Machine learning (ML) with data science-enabled lung cancer diagnosis and classification (MLDS-LCDC) | CT | 98.11 | 97.01 | 98.64 |  |  |  |  | High | High |
| Ganashree TS (2025) [175] | Thailand | ‒ | CNN model | CT | 95.0 |  | 97.0 | 94.0 |  |  | 95.0 | High | High |
| Zhanlin Ji (2023) [176] | China | ‒ | ELCT-YOLO model | CT |  |  |  | 92.3 |  | 95.1 |  | Unclear | Unclear |
| B Karthikeyan (2024) [177] | India | ‒ | Optimal transfer learning method for lung cancer detection and classification (MFFOTL-LCDC) | CT | 99.05 | 98.41 | 99.26 | 98.72 |  |  |  | High | High |
| Furkan Berk Seyrek (2024) [178] | Turkey | 1010 | Deep learning model | CT | 98.41 | 98.39 | 97.95 |  |  |  | 98.17 | Unclear | High |
| A Angel Mary (2024) [179] | India | 1010 | Block chain and deep learning with dynamic pattern features | Low-dose CT | 98.64 |  |  | 92.42 |  | 93.76 | 93.08 | Unclear | High |
| C Venkatesh (2020) [180] | India | ‒ | Bio-inspired algorithm | CT | 93.53 | 92.96 | 98.52 |  |  |  |  | High | High |
| Xiaofeng Lin (2021) [181] | China | 915 | Clinical image radiomics deep learning (CIRDL) model | CT | 84.82 | 87.63 | 79.09 |  | 90.69 |  | 88.54 | Low | Low |
| Atul Tiwari (2023) [182] | India | ‒ | Hybrid recurrent neural network generative adversarial network (RNN-GAN) model | CT | 98.6 |  |  | 100 |  | 99.0 |  | High | High |
| Ahmed Elnakib (2020) [183] | Egypt | 50 | Deep learning optimization model | CT | 96.25 | 97.5 | 95.0 |  |  |  |  | Low | High |
| Roopa Chandrika R (2023) [184] | India | ‒ | Deep learning model | CT | 99.6 |  | 99.2 | 98.6 |  | 99.2 |  | Unclear | High |
| Vikul J Pawar (2023) [185] | India | ‒ | Modified cat swarm optimization ‒ layer fused conventional neural network (MCSO+LF-CNN) model | CT | 91.57 |  |  |  |  |  |  | High | High |
| Wei Fan (2024) [186] | China | 130 | Artificial intelligence model | CT |  | 95.40 | 75.0 |  | 79.8 |  |  | Low | Low |
| Benjamin Hunter (2022) [187] | UK | 499 | Radiomics-based decision support tool | CT | 76.0 | 90.0 | 53.0 |  |  |  | 83.0 | Low | Low |
| Sneha S Nair (2024) [188] | India | 1010 | Random walker- artificial neural networks (RW-ANN) | CT | 96.82 | 99.35 | 63.17 | 97.28 |  |  | 98.31 | Low | Low |
| Kang Qi (2024) [189] | China | 402 | Lung-PNet | CT | 76.9 | 50.0 | 92.0 |  |  |  | 60.9 | Low | Low |
| Mian Muhammad Naeem Abid (2021) [190] | Pakistan | ‒ | Multi-view convolutional recurrent neural network (MV-CRecNet) | CT | 97.1 | 97.5 | 96.6 | 96.7 |  |  | 97.1 | Low | Low |
| Pavan Kumar Pagadala (2023) [191] | India | ‒ | Deep neural networks model | CT | 99.26 | 99.11 | 99.12 |  |  |  |  | High | High |
| Bin Li (2022) [192] | China | ‒ | Computer aided diagnosis (CAD) model | CT | 77.61 |  |  |  |  |  |  | Unclear | Low |
| A Asuntha (2020) [193] | India | ‒ | Fuzzy particle swarm optimization convolution neural network (FPSOCNN) | CT | 94.97 | 96.68 | 95.89 |  |  |  |  | Low | Low |
| Abdulqader Faris Abdulqader (2025) [194] | Iraq | 1608 | Multi‑objective deep learning model | CT | 97.11 |  |  | 97.99 |  | 97.33 |  | Low | Low |
| Anindita Saha (2024) [195] | India | 1653 | Novel transfer learning model (VER-Net) | CT | 91.0 |  |  | 92.0 |  | 91.0 | 91.3 | Low | Low |
| Han Yang (2022) [196] | China | 93 | Pulmonary nodules artificial intelligence diagnostic system (PNAIDS) model | CT |  |  |  |  | 69.6 |  |  | Unclear | Unclear |
| Ran Ni (2025) [197] | China | 1121 | Early lung cancer diagnosis model | CT |  | 92.0 | 97.0 |  | 99.0 |  |  | Low | Low |
| Chengping Zhang (2024) [198] | China | ‒ | Dense Net-CNN model | CT | 99.3 |  | 99.6 | 99.3 |  | 99.3 | 99.3 | Low | Low |
| Asghar Ali Shah (2023) [199] | Pakistan | ‒ | Deep learning ensemble 2D CNN model | CT | 95.0 |  |  | 93.0 |  | 80.0 |  | Low | Low |
| Sana Alazwari (2024) [200] | Saudi Arabia | ‒ | Computer-aided diagnosis for lung cancer by utilizing the waterwheel plant algorithm with deep learning (CADLC-WWPADL) | CT | 99.05 | 98.55 | 99.35 | 98.33 |  |  |  | Low | Low |
| Mohamed Hammad (2025) [201] | Saudi Arabia | ‒ | Explainable AI gradient-weighted class activation mapping (Grad-CAM) model | CT | 93.06 |  |  | 95.53 | 97.25 | 93.09 | 93.84 | Low | Low |
| Ward Hendrix (2023) [202] | Netherlands | 100 | Artificial intelligence model | CT |  | 96.9 |  |  |  |  |  | Unclear | Low |
| Gür Emre Güraksın (2025) [203] | Turkey | 110 | Lung ensemble convolutional neural network (LECNN) model | CT | 99.0 | 98.82 | 99.48 | 99.06 |  |  | 98.94 | Low | Low |
| Shaohua Zheng (2021) [204] | China | ‒ | Radiology analysis and malignancy evaluation network (R2MNet) model | CT | 94.74 |  |  |  | 97.52 |  |  | Low | Unclear |

**References**

1 SB, P. Performance analysis of lung cancer detection and classification using efficientNet: a deep learning model. Multimed Tools Appl (2024).

2 AR, B., RS, V. K. & SS, K. LCD-capsule network for the detection and classification of lung cancer on computed tomography images. Multimedia Tools and Applications 82, 37573-37592 (2023).

3 Aharonu, M. & Ramasamy, L. K. An intelligent generative adversarial network multistage lung cancer detection and subtypes classification. International Journal of Machine Learning and Cybernetics, 1-24 (2024).

4 Sundaresan, A. A. Automatic lung cancer detection in low-dose lung CTs using transfer learning. Journal of Advanced Research in Dynamical and Control Systems 10, 195-201 (2018).

5 Alksas, A. et al. A novel higher order appearance texture analysis to diagnose lung cancer based on a modified local ternary pattern. Computer Methods and Programs in Biomedicine 240, 107692 (2023).

6 Almukhtar, F. H. Lung cancer diagnosis through CT images using principal component analysis (PCA) and error correcting output codes (ECOC). Journal of Control and Decision 11, 472-482 (2024).

7 Alshayeji, M. H. & Abed, S. e. Lung cancer classification and identification framework with automatic nodule segmentation screening using machine learning. Applied Intelligence 53, 19724-19741 (2023).

8 Arumugam, S. R., Ravichandran, B., Baskaran, D. & Annamalai, R. Lung Lobe Segmentation and lung Cancer Detection with Hybrid Optimization Enabled Deep Learning Using CT Images. Journal of Mechanics in Medicine and Biology (2024).

9 Bagheri Tofighi, A., Ahmadi, A. & Mosadegh, H. Improving lung cancer detection via MobileNetV2 and stacked-GRU with explainable AI. International Journal of Information Technology 17, 1189-1196 (2025).

10 Bai, Y., Li, D., Duan, Q. & Chen, X. Analysis of high-resolution reconstruction of medical images based on deep convolutional neural networks in lung cancer diagnostics. Computer Methods and Programs in Biomedicine 217, 106592 (2022).

11 Balaji, P., Aluvalu, R. & Sagar, K. Residual attention network based hybrid convolution network model for lung cancer detection. Intelligent Decision Technologies 17, 1475-1488 (2023).

12 Bhattacharjee, A., Murugan, R., Majumder, S. & Goel, T. Neural network–based computer-aided lung cancer detection. Research on Biomedical Engineering 37, 657-671 (2021).

13 Bhattacharjee, A., Murugan, R., Soni, B. & Goel, T. Ada-gridrf: A fast and automated adaptive boost based grid search optimized random forest ensemble model for lung cancer detection. Physical and Engineering Sciences in Medicine 45, 981-994 (2022).

14 Bushara, A., Kumar, R. V. & Kumar, S. An ensemble method for the detection and classification of lung cancer using Computed Tomography images utilizing a capsule network with Visual Geometry Group. Biomedical Signal Processing and Control 85, 104930 (2023).

15 Chapala, V. & Bojja, P. IoT based lung cancer detection using machine learning and cuckoo search optimization. International Journal of Pervasive Computing and Communications 17, 549-562 (2021).

16 Chen, L. & Zhang, Z. The self‐distillation trained multitask dense‐attention network for diagnosing lung cancers based on CT scans. Medical Physics 51, 1738-1753 (2024).

17 Dawood, H., Nawaz, M., Ilyas, M. U., Nazir, T. & Javed, A. Attention-guided CenterNet deep learning approach for lung cancer detection. Computers in Biology and Medicine 186, 109613 (2025).

18 DEVI, S. et al. TRANSITIONING FROM RADIOGRAPHS TO PRECISION DIAGNOSTICS FOR ATYPICAL CARCINOIDS, ADENOSQUAMOUS CARCINOMA, AND MUCOEPIDERMOID CARCINOMA USING ADVANCED MACHINE LEARNING ALGORITHMS. Journal of the Balkan Tribological Association 30 (2024).

19 Dutta, A. K. Detecting Lung Cancer Using Machine Learning Techniques. Intelligent Automation & Soft Computing 31 (2022).

20 Gampala, V., Ramya, V., Maram, B. & Pappu, S. R. Identification of lung cancer using archimedes flow regime optimization enabled deep belief network. Multimedia Tools and Applications 83, 78659-78688 (2024).

21 Gautam, N., Basu, A. & Sarkar, R. Lung cancer detection from thoracic CT scans using an ensemble of deep learning models. Neural Computing and Applications 36, 2459-2477 (2024).

22 Heidari, A. et al. A new lung cancer detection method based on the chest CT images using Federated Learning and blockchain systems. Artificial intelligence in medicine 141, 102572 (2023).

23 Helaly, H. A., Badawy, M., El-Gendy, E. M. & Haikal, A. Y. Elcd-nsc2: a novel early lung cancer detection and non-small cell classification framework. Neural Computing and Applications 36, 15149-15164 (2024).

24 Hou, S. Auxiliary tumour diagnosis image with deep learning technology. The Journal of Supercomputing 78, 578-595 (2022).

25 Huidrom, R., Chanu, Y. J. & Singh, K. M. Neuro-evolutional based computer aided detection system on computed tomography for the early detection of lung cancer. Multimedia Tools and Applications 81, 32661-32673 (2022).

26 Jain, R., Singh, P. & Kaur, A. An ensemble reinforcement learning-assisted deep learning framework for enhanced lung cancer diagnosis. Swarm and Evolutionary Computation 91, 101767 (2024).

27 Kanipriya, M., Hemalatha, C., Sridevi, N., SriVidhya, S. & Shabu, S. J. An improved capuchin search algorithm optimized hybrid CNN-LSTM architecture for malignant lung nodule detection. Biomedical Signal Processing and Control 78, 103973 (2022).

28 Kashyap, M. et al. Automated Deep Learning-Based Detection and Segmentation of Lung Tumors at CT. Radiology 314, e233029 (2025).

29 Kavitha, S. et al. An optimized multi-head attention based fused depthwise convolutional model for lung cancer detection. Expert Systems with Applications, 126596 (2025).

30 Khalefa, S. J. Finite element method and hybrid deep learning approaches: high-accuracy lung cancer detection model. Multiscale and Multidisciplinary Modeling, Experiments and Design 7, 3017-3029 (2024).

31 Nagaraj, P. Lung cancer detection and classification using optimized CNN features and Squeeze-Inception-ResNeXt model. Computational Biology and Chemistry 117, 108437 (2025).

32 Lanjewar, M. G., Panchbhai, K. G. & Charanarur, P. Lung cancer detection from CT scans using modified DenseNet with feature selection methods and ML classifiers. Expert Systems with Applications 224, 119961 (2023).

33 Liang, Z. J. et al. Leveraging prior knowledge in machine intelligence to improve lesion diagnosis for early cancer detection. Medical Physics (2025).

34 Ma, L., Wu, H. & Samundeeswari, P. Googlenet-al: A fully automated adaptive model for lung cancer detection. Pattern Recognition 155, 110657 (2024).

35 Mammeri, S., Amroune, M., Haouam, M.-Y., Bendib, I. & Corrêa Silva, A. Early detection and diagnosis of lung cancer using YOLO v7, and transfer learning. Multimedia Tools and Applications 83, 30965-30980 (2024).

36 Mariappan, S. & Moses, D. Deep learning-based lung cancer detection using CT images. International Journal of Ad Hoc and Ubiquitous Computing 47, 143-157 (2024).

37 Muthazhagan, B., Ravi, T. & Rajinigirinath, D. Sooty-LuCaNet: Sooty tern optimization based deep learning network for lung cancer detection. Journal of Intelligent & Fuzzy Systems 45, 8823-8836 (2023).

38 Nagaraja, P. & Chennupati, S. K. Integration of adaptive segmentation with heuristic-aided novel ensemble-based deep learning model for lung cancer detection using CT images. Intelligent Decision Technologies 17, 1135-1160 (2023).

39 Navaneethakrishnan, M., Anand, M. V., Vasavi, G. & Rani, V. V. Deep Fuzzy SegNet-based lung nodule segmentation and optimized deep learning for lung cancer detection. Pattern Analysis and Applications 26, 1143-1159 (2023).

40 Ozcelik, N., Kıvrak, M., Kotan, A. & Selimoğlu, İ. Lung cancer detection based on computed tomography image using convolutional neural networks. Technology and Health Care 32, 1795-1805 (2024).

41 Park, Y.-J., Choi, D., Choi, J. Y. & Hyun, S. H. Performance evaluation of a deep learning system for differential diagnosis of lung cancer with conventional CT and FDG PET/CT using transfer learning and metadata. Clinical Nuclear Medicine 46, 635-640 (2021).

42 Parvathy, C. & Jayan, J. Automatic Lung Cancer Detection Using Computed Tomography Based on Chan Vese Segmentation and SENET. Optical Memory and Neural Networks 33, 339-354 (2024).

43 Pethuraj, M. S., Aboobaider, B. b. M. & Salahuddin, L. B. Analyzing CT images for detecting lung cancer by applying the computational intelligence‐based optimization techniques. Computational Intelligence 39, 930-949 (2023).

44 Prasad, U., Chakravarty, S. & Mahto, G. Lung cancer detection and classification using deep neural network based on hybrid metaheuristic algorithm. Soft Computing 28, 8579-8602 (2024).

45 Quasar, S. R. et al. Ensemble methods for computed tomography scan images to improve lung cancer detection and classification. Multimedia Tools and Applications 83, 52867-52897 (2024).

46 Rathan, N. & Lokesh, S. Enhanced Lung Cancer Diagnosis and Staging With HRNeT: A Deep Learning Approach. International Journal of Imaging Systems and Technology 34, e23193 (2024).

47 Rehman, A. et al. Detection of lungs tumors in ct scan images using convolutional neural networks. IEEE/ACM Transactions on Computational Biology and Bioinformatics 21, 769-777 (2023).

48 Sabzalian, M. H. et al. New bidirectional recurrent neural network optimized by improved Ebola search optimization algorithm for lung cancer diagnosis. Biomedical Signal Processing and Control 84, 104965 (2023).

49 Shaikh, N. & Shah, P. Automated lung cancer diagnosis using swarm intelligence with deep learning. Computer Methods in Biomechanics and Biomedical Engineering: Imaging & Visualization 11, 2363-2385 (2023).

50 Shakeel, P. M., Burhanuddin, M. & Desa, M. I. Automatic lung cancer detection from CT image using improved deep neural network and ensemble classifier. Neural Computing and Applications, 1-14 (2022).

51 Shanid, M. & Anitha, A. Adaptive optimisation driven deep belief networks for lung cancer detection and severity level classification. International Journal of Bio-Inspired Computation 18, 114-121 (2021).

52 Shen, Z., Cao, P., Yang, J. & Zaiane, O. R. WS-LungNet: A two-stage weakly-supervised lung cancer detection and diagnosis network. Computers in Biology and Medicine 154, 106587 (2023).

53 Shi, F. et al. Semi-supervised deep transfer learning for benign-malignant diagnosis of pulmonary nodules in chest CT images. IEEE transactions on medical imaging 41, 771-781 (2021).

54 Siddiqui, E. A., Chaurasia, V. & Shandilya, M. Detection and classification of lung cancer computed tomography images using a novel improved deep belief network with Gabor filters. Chemometrics and Intelligent Laboratory Systems 235, 104763 (2023).

55 Singh, A., Dwivedi, R. K. & Rastogi, R. Machine Learning Based Framework for Lung Cancer Detection and Image Feature Extraction Using VGG16 with PCA on CT-Scan Images. SN Computer Science 5, 1040 (2024).

56 Sivasankaran, P. & Dhanaraj, K. R. Lung Cancer Detection Using Image Processing Technique Through Deep Learning Algorithm. Revue d'Intelligence Artificielle 38 (2024).

57 Sridevi, S. & RajivKannan, A. Development of 3DTDUnet++ with novel function and multi-scale dilated-based deep learning model for lung cancer diagnosis using CT images. Biomedical Signal Processing and Control 94, 106243 (2024).

58 Sudha, R. & Maheswari, K. U. Automatic lung cancer detection using hybrid particle snake swarm optimization with optimized mask RCNN. Multimedia Tools and Applications 83, 76807-76831 (2024).

59 Sudha, R. & Maheswari, K. Adaptive pelican optimization with optimized mask RCNN for automatic lung cancer detection. Multimedia Tools and Applications, 1-21 (2025).

60 Usharani, C. & Selvapandian, A. FedLRes: enhancing lung cancer detection using federated learning with convolution neural network (ResNet50). Neural Computing and Applications, 1-12 (2025).

61 Vadala, L. R., Das, M., Madhuri, C. R. & Merugula, S. SpiLenet based detection and severity level classification of lung cancer using CT images. Computers and Electrical Engineering 123, 110036 (2025).

62 Velmurugan, N., Rajeswari, R., Naganjaneyulu, S. & Anupama, A. Rat swarm political optimizer based deep learning approach for lung lobe segmentation and lung cancer detection using CT images. Biomedical Signal Processing and Control 105, 107612 (2025).

63 Venkatesh, C. & Bojja, P. A dynamic optimization and deep learning technique for detection of lung cancer in CT images and data access through Internet of Things. Wireless Personal Communications 125, 2621-2646 (2022).

64 Wang, G. et al. Lung cancer scRNA-seq and lipidomics reveal aberrant lipid metabolism for early-stage diagnosis. Science translational medicine 14, eabk2756 (2022).

65 Wang, M., Yang, Z. & Zhao, R. Advancing lung cancer diagnosis: Combining 3D auto-encoders and attention mechanisms for CT scan analysis. Journal of X-Ray Science and Technology, 08953996241313120 (2025).

66 Wani, N. A., Kumar, R. & Bedi, J. DeepXplainer: An interpretable deep learning based approach for lung cancer detection using explainable artificial intelligence. Computer Methods and Programs in Biomedicine 243, 107879 (2024).

67 Xu, Y. et al. Identification of benign and malignant lung nodules in CT images based on ensemble learning method. Interdisciplinary Sciences: Computational Life Sciences, 1-11 (2022).

68 Xu, Y., Wang, Y. & Razmjooy, N. Lung cancer diagnosis in CT images based on Alexnet optimized by modified Bowerbird optimization algorithm. Biomedical Signal Processing and Control 77, 103791 (2022).

69 Yan, C. & Razmjooy, N. Optimal lung cancer detection based on CNN optimized and improved Snake optimization algorithm. Biomedical Signal Processing and Control 86, 105319 (2023).

70 Yang, R. et al. Development and external validation of a multimodal integrated feature neural network (MIFNN) for the diagnosis of malignancy in small pulmonary nodules (≤ 10 mm). Biomedical Physics & Engineering Express 10, 045008 (2024).

71 Zaben, S. O. Improving lung cancer diagnoses: a machine learning approach for detection and prediction in CT-Scan image analysis. Service Oriented Computing and Applications, 1-16 (2024).

72 Zheng, Q. et al. Reconstruction error based implicit regularization method and its engineering application to lung cancer diagnosis. Engineering Applications of Artificial Intelligence 139, 109439 (2025).

73 Thanammal, K. Lung cancer detection via deep learning-based pyramid network with honey badger algorithm. Measurement: Sensors 31, 100993 (2024).

74 Wankhade, S. & Vigneshwari, S. A novel hybrid deep learning method for early detection of lung cancer using neural networks. Healthcare Analytics 3, 100195 (2023).

75 Lee, J., Chun, J., Kim, H., Kim, J. S. & Park, S. Y. Development and evaluation of an integrated model based on a deep segmentation network and demography-added radiomics algorithm for segmentation and diagnosis of early lung adenocarcinoma. Computerized Medical Imaging and Graphics 109, 102299 (2023).

76 Lee, J. H. et al. Improved unsupervised 3D lung lesion detection and localization by fusing global and local features: Validation in 3D low-dose computed tomography. Medical Image Analysis 103, 103559 (2025).

77 Masood, A. et al. Computer-assisted decision support system in pulmonary cancer detection and stage classification on CT images. Journal of biomedical informatics 79, 117-128 (2018).

78 Zhang, Y. et al. Multi-omics model is an effective means to diagnose benign and malignant pulmonary nodules. Clinics 80, 100599 (2025).

79 Jayapradha, J. et al. EMI-LTI: An enhanced integrated model for lung tumor identification using Gabor filter and ROI. MethodsX 14, 103247 (2025).

80 Hammad, M., ElAffendi, M., Asim, M., Abd El-Latif, A. A. & Hashiesh, R. Automated lung cancer detection using novel genetic TPOT feature optimization with deep learning techniques. Results in Engineering 24, 103448 (2024).

81 Pandian, R., Vedanarayanan, V., Kumar, D. R. & Rajakumar, R. Detection and classification of lung cancer using CNN and Google net. Measurement: Sensors 24, 100588 (2022).

82 Shatnawi, M. Q., Abuein, Q. & Al-Quraan, R. Deep learning-based approach to diagnose lung cancer using CT-scan images. Intelligence-Based Medicine 11, 100188 (2025).

83 Abe, A. A. et al. A Robust Deep Learning Algorithm for Lung Cancer Detection from Computed Tomography Images. Intelligence-Based Medicine, 100203 (2025).

84 Tsivgoulis, M., Papastergiou, T. & Megalooikonomou, V. An improved SqueezeNet model for the diagnosis of lung cancer in CT scans. Machine Learning with Applications 10, 100399 (2022).

85 Maleki, N. & Niaki, S. T. A. An intelligent algorithm for lung cancer diagnosis using extracted features from Computerized Tomography images. Healthcare Analytics 3, 100150 (2023).

86 Yanagawa, M. et al. Diagnostic performance for pulmonary adenocarcinoma on CT: comparison of radiologists with and without three-dimensional convolutional neural network. European Radiology 31, 1978-1986 (2021).

87 Alamgeer, M. et al. Deep Learning Enabled Computer Aided Diagnosis Model for Lung Cancer using Biomedical CT Images. Computers, Materials & Continua 73 (2022).

88 Gonçalves, L., Novo, J., Cunha, A. & Campilho, A. Learning lung nodule malignancy likelihood from radiologist annotations or diagnosis data. Journal of Medical and Biological Engineering 38, 424-442 (2018).

89 Toğaçar, M., Ergen, B. & Cömert, Z. Detection of lung cancer on chest CT images using minimum redundancy maximum relevance feature selection method with convolutional neural networks. Biocybernetics and Biomedical Engineering 40, 23-39 (2020).

90 Yang, K. et al. Identification of benign and malignant pulmonary nodules on chest CT using improved 3D U-Net deep learning framework. European journal of radiology 129, 109013 (2020).

91 Abe, A., Nyathi, M. & Okunade, A. Lung cancer diagnosis from computed tomography scans using convolutional neural network architecture with Mavage pooling technique. AIMS Medical Science 12, 13-27 (2025).

92 Bhukya, S., Ganagoni, V., Nangunoori, S. S. & Enapothula, S. T. A Deep Learning Framework Using Enhanced Convolutional Neural Network for Detection of Lung Cancer from CT Images. International Research Journal of Multidisciplinary Technovation 7, 138-150 (2025).

93 JENITA SUBASH, D. K. S. OCHOA-RNN: OPPOSITIONAL BASED CHIMP OPTIMIZATION ALGORITHM (OCHOA) AND RECURRENT

NEURAL NETWORK (RNN) HYBRID CLASSIFIER MODEL FOR LUNG CANCER DIAGNOSIS. Journal of Theoretical and Applied Information Technology 101, 2442-2458 (2023).

94 Taher, K. F., Ali, A. M. & Hadi, G. M. MASK-RCNN on the diagnoses of lung cancer in Kurdistan Region of Iraq. Zanco Journal of Pure and Applied Sciences 36, 40-48 (2024).

95 Thangavel, C., Palanichamy, J. & Chinnasamy, S. An advanced perceptual U-Net segmentation based DDO-SDN classification system for lung pulmonary cancer detection. Imaging 15, 12-22 (2023).

96 Prasad, J., Chakravarty, S. & Krishna, M. V. Lung cancer detection using an integration of fuzzy K-means clustering and deep learning techniques for CT lung images. Bulletin of the Polish Academy of Sciences Technical Sciences, e139006-e139006 (2022).

97 Qadir, A. M., Abdalla, P. A. & Abd, D. F. A Hybrid Lung Cancer Model for Diagnosis and Stage Classification from Computed Tomography Images. Iraqi Journal for Electrical & Electronic Engineering 20 (2024).

98 Mostafa, B., Sakr, M. & Keshk, A. Employing the Capabilities of LSTM and Bi-LSTM for Lung Cancer Detection and Classification. International Journal of Intelligent Engineering & Systems 17 (2024).

99 Ozdemir, O., Russell, R. L. & Berlin, A. A. A 3D probabilistic deep learning system for detection and diagnosis of lung cancer using low-dose CT scans. IEEE transactions on medical imaging 39, 1419-1429 (2019).

100 Elloumi, N., Seddik, H., Chaabane, S. B. & Nadra, T. A 3D Processing Technique to Detect Lung Tumor. 2023 Published by (IJACSA) (2023).

101 Pang, S., Zhang, Y., Ding, M., Wang, X. & Xie, X. A deep model for lung cancer type identification by densely connected convolutional networks and adaptive boosting. IEEE Access 8, 4799-4805 (2019).

102 Chenyang, L. & Chan, S.-C. A joint detection and recognition approach to lung cancer diagnosis from CT images with label uncertainty. IEEE Access 8, 228905-228921 (2020).

103 Bhavani Krishna, G. M. T. Advanced mask region-based convolutional neural network based deep-learning model for lung cancer detection. IAES International Journal of Artificial Intelligence 14, 1179-1186 (2024).

104 Duan, S. et al. Development of a machine learning-based multimode diagnosis system for lung cancer. Aging (Albany NY) 12, 9840 (2020).

105 Chowdary, S. & Purushotaman, S. B. An Improved Archimedes Optimization-aided Multi-scale Deep Learning Segmentation with dilated ensemble CNN classification for detecting lung cancer using CT images. Network: Computation in Neural Systems, 1-39 (2024).

106 Jakimovski, G. & Davcev, D. Using double convolution neural network for lung cancer stage detection. Applied Sciences 9, 427 (2019).

107 Ozdemir, B., Aslan, E. & Pacal, I. Attention Enhanced InceptionNeXt Based Hybrid Deep Learning Model for Lung Cancer Detection. IEEE Access (2025).

108 Hussain Ali, Y. et al. Optimization system based on convolutional neural network and internet of medical things for early diagnosis of lung cancer. Bioengineering 10, 320 (2023).

109 Raptis, S., Ilioudis, C. & Theodorou, K. Uncovering the Diagnostic Power of Radiomic Feature Significance in Automated Lung Cancer Detection: An Integrative Analysis of Texture, Shape, and Intensity Contributions. BioMedInformatics 4, 2400-2425 (2024).

110 Liu, A. et al. Preoperative diagnosis of malignant pulmonary nodules in lung cancer screening with a radiomics nomogram. Cancer Communications 40, 16-24 (2020).

111 Shafi, I. et al. An effective method for lung cancer diagnosis from ct scan using deep learning-based support vector network. Cancers 14, 5457 (2022).

112 Sollini, M. et al. The Development of an Intelligent Agent to Detect and Non-Invasively Characterize Lung Lesions on CT Scans: Ready for the “Real World”? Cancers 15, 357 (2023).

113 Alamgeer, M., Alruwais, N., Alshahrani, H. M., Mohamed, A. & Assiri, M. Dung beetle optimization with deep feature fusion model for lung cancer detection and classification. Cancers 15, 3982 (2023).

114 Masood, A. et al. Cloud-based automated clinical decision support system for detection and diagnosis of lung cancer in chest CT. IEEE journal of translational engineering in health and medicine 8, 1-13 (2019).

115 Zhang, L. et al. Application Potential of Radiomics based on the Unenhanced CT Image for the Identification of Benign or Malignant Pulmonary Nodules. Current Medical Imaging 20, e15734056246425 (2024).

116 Song, G., Dai, Q., Nie, Y. & Chen, G. Differential diagnosis of benign and malignant pulmonary nodules in ct images based on multitask learning. Current Medical Imaging 20, e15734056252399 (2024).

117 Ansari, M. M. et al. SVMVGGNet-16: A Novel Machine and Deep Learning Based Approaches for Lung Cancer Detection using Combined SVM and VGGNet-16. Current Medical Imaging 21, e15734056348824 (2025).

118 Alzubaidi, M. A., Otoom, M. & Jaradat, H. Comprehensive and comparative global and local feature extraction framework for lung cancer detection using CT scan images. IEEe Access 9, 158140-158154 (2021).

119 Zhang, X., Wang, X., Huang, T. & Sheng, J. Deep Learning-Powered Lung Cancer Diagnosis: Harnessing IoT Medical Data and CT Images. International Journal of Advanced Computer Science & Applications 15 (2024).

120 Aydın, N. et al. Detection of lung cancer on computed tomography using artificial intelligence applications developed by deep learning methods and the contribution of deep learning to the classification of lung carcinoma. Current Medical Imaging Reviews 17, 1137-1141 (2021).

121 Gunjan, V. K., Singh, N., Shaik, F. & Roy, S. Detection of lung cancer in CT scans using grey wolf optimization algorithm and recurrent neural network. Health and Technology 12, 1197-1210 (2022).

122 Sori, W. J., Feng, J., Godana, A. W., Liu, S. & Gelmecha, D. J. DFD-Net: lung cancer detection from denoised CT scan image using deep learning. Frontiers of Computer Science 15, 1-13 (2021).

123 Cho, H. S., Hwang, E. J., Yi, J., Choi, B. & Park, C. M. Artificial intelligence system for identification of overlooked lung metastasis in abdominopelvic computed tomography scans of patients with malignancy. Diagnostic and Interventional Radiology 31, 102 (2025).

124 VR, N. & Chandra SS, V. ExtRanFS: An automated lung cancer malignancy detection system using extremely randomized feature selector. Diagnostics 13, 2206 (2023).

125 Hsiao, C.-C., Peng, C.-H., Wu, F.-Z. & Cheng, D.-C. Impact of Voxel Normalization on a Machine Learning-Based Method: A Study on Pulmonary Nodule Malignancy Diagnosis Using Low-Dose Computed Tomography (LDCT). Diagnostics 13, 3690 (2023).

126 Bhatia, I., Aarti, Ansarullah, S. I., Amin, F. & Alabrah, A. Lightweight Advanced Deep Neural Network (DNN) Model for Early-Stage Lung Cancer Detection. Diagnostics 14, 2356 (2024).

127 Bouamrane, A. et al. Toward Robust Lung Cancer Diagnosis: Integrating Multiple CT Datasets, Curriculum Learning, and Explainable AI. Diagnostics 15, 1 (2024).

128 Rajeev Dixit, P. K., Shashank Ojha. Implementing Deep Learning Models for Early Detection and Segmentation of Lung Cancer from Medical Imaging. Communications on Applied Nonlinear Analysis 32, 545-567 (2025).

129 Rana M Mahmoud, M. E., Mohamed Taha. 3D visualization diagnostics for lung cancer detection. IAES International Journal of Artificial Intelligence 13, 4630-4641 (2024).

130 Yamada, D. et al. Multimodal modeling with low-dose CT and clinical information for diagnostic artificial intelligence on mediastinal tumors: a preliminary study. BMJ Open Respiratory Research 11, e002249 (2024).

131 Kavitha Belegere Chandraiah, N. K. B. An optimal model for detection of lung cancer using convolutional neural network. Indonesian Journal of Electrical Engineering and Computer Science 34, 134-143 (2024).

132 Nair, S. S., Devi, V. M. & Bhasi, S. Lung Cancer Detection from CT Images: Modified Adaptive Threshold Segmentation with Support Vector Machines and Artificial Neural Network Classifier. Current Medical Imaging 20, e140723218727 (2024).

133 Ponnada, V. T. & Srinivasu, S. N. Efficient CNN for lung cancer detection. Int J Recent Technol Eng 8, 3499-3505 (2019).

134 SU, A., PP, F. R., Abraham, A. & Stephen, D. Deep learning-based BoVW–CRNN model for lung tumor detection in nano-segmented CT images. Electronics 12, 14 (2022).

135 Wehbe, A., Dellepiane, S. & Minetti, I. Enhanced Lung Cancer Detection and TNM Staging Using YOLOv8 and TNMClassifier: An Integrated Deep Learning Approach for CT Imaging. IEEE Access (2024).

136 Tawfik, N. et al. Enhancing Early Detection of Lung Cancer through Advanced Image Processing Techniques and Deep Learning Architectures for CT Scans. Computers, Materials and Continua 81, 271-307 (2024).

137 Vemula, S. T. et al. Deep Learning Techniques for Lung Cancer Recognition. Engineering, Technology & Applied Science Research 14, 14916-14922 (2024).

138 Sayana Sharma, M. K., Deepak Saini. Lung Cancer Detection using Convolutional Neural Network. International Journal of Engineering and Advanced Technology 8, 3256-3262 (2019).

139 Bhattacharjee, A. et al. A multi-class deep learning model for early lung cancer and chronic kidney disease detection using computed tomography images. Frontiers in Oncology 13, 1193746 (2023).

140 Karimullah, S., Khan, M., Shaik, F., Alabduallah, B. & Almjally, A. An integrated method for detecting lung cancer via CT scanning via optimization, deep learning, and IoT data transmission. Frontiers in Oncology 14, 1435041 (2024).

141 Venkatesh, C. et al. A neural network and optimization based lung cancer detection system in CT images. Frontiers in public health 10, 769692 (2022).

142 Saxena, S. & Prasad, N. Design of novel convolution neural network model for lung cancer detection by using sensitivity maps. Int J Artif Intell ISSN 2252, 3219 (2024).

143 Pang, S. et al. VGG16-T: a novel deep convolutional neural network with boosting to identify pathological type of lung cancer in early stage by CT images. International Journal of Computational Intelligence Systems 13, 771-780 (2020).

144 Kumar, S. Optimized Lung Cancer Identification in CT Imaging: A Synergistic Deep Learning Approach with Residual U-Net Segmentation and Swin Transformer Feature Extraction. International Journal of Computer Information Systems and Industrial Management Applications 17, 16-16 (2025).

145 N Raghapriya, Y. K. Lung Cancer Detection Using Integration of Hybrid Segmentation Approach and DL Techniques. SSRG International Journal of Electronics and Communication Engineering 11, 148-157 (2024).

146 Raghuvanshi, S. S., Arya, K. & Patel, V. PSbBO-Net: A Hybrid Particle Swarm and Bayesian Optimization-based DenseNet for Lung Cancer Detection using Histopathological and CT Images. International Journal of Electrical and Electronics Research 12, 1074-1086 (2024).

147 Wang, Y. et al. Leveraging serial low-dose CT scans in radiomics-based reinforcement learning to improve early diagnosis of lung cancer at baseline screening. Radiology: Cardiothoracic Imaging 6, e230196 (2024).

148 Huang, L. et al. Unveiling large multimodal models in pulmonary CT: A comparative assessment of generative AI performance in lung cancer diagnostics. View, 20250077 (2025).

149 Al-Huseiny, M. S. & Sajit, A. S. Transfer learning with GoogLeNet for detection of lung cancer. Indonesian Journal of Electrical Engineering and computer science 22, 1078-1086 (2021).

150 Tiwari, L. et al. Detection of lung nodule and cancer using novel Mask-3 FCM and TWEDLNN algorithms. Measurement 172, 108882 (2021).

151 Tian, Q., Wu, Y., Ren, X. & Razmjooy, N. A new optimized sequential method for lung tumor diagnosis based on deep learning and converged search and rescue algorithm. Biomedical Signal Processing and Control 68, 102761 (2021).

152 Shanid, M. & Anitha, A. Lung cancer detection from CT images using salp-elephant optimization-based deep learning. Biomedical Engineering: Applications, Basis and Communications 32, 2050001 (2020).

153 Saleem, M. A. et al. Sooty tern optimization algorithm-based deep learning model for diagnosing NSCLC tumours. Sensors 23, 2147 (2023).

154 Rakesh Sankaran, S. S., Lakshay Jeet Singh, Jaspreet Sidhu, Anisha Chaudhary, Jagtej Singh. Deep learning-based computerized diagnosis of lung cancer. Salud, Ciencia y Tecnologia 4, 1-8 (2024).

155 Ding, Y. et al. Improving the efficiency of identifying malignant pulmonary nodules before surgery via a combination of artificial intelligence CT image recognition and serum autoantibodies. European Radiology 33, 3092-3102 (2023).

156 Klangbunrueang, R., Pookduang, P., Chansanam, W. & Lunrasri, T. in Informatics. 18 (MDPI).

157 Ponnada, V. T. & Srinivasu, S. N. Integrated clinician decision supporting system for pneumonia and lung cancer detection. International Journal of Innovative Technology and Exploring Engineering (IJITEE) (2019).

158 Jacobs, C. et al. Deep learning for lung cancer detection on screening CT scans: results of a large-scale public competition and an observer study with 11 radiologists. Radiology: Artificial Intelligence 3, e210027 (2021).

159 Gharaibeh, N. Y., De Fazio, R., Al-Naami, B., Al-Hinnawi, A.-R. & Visconti, P. Automated Lung Cancer Diagnosis Applying Butterworth Filtering, Bi-Level Feature Extraction, and Sparce Convolutional Neural Network to Luna 16 CT Images. Journal of Imaging 10, 168 (2024).

160 Shafiee, M. J., Chung, A. G., Khalvati, F., Haider, M. A. & Wong, A. Discovery radiomics via evolutionary deep radiomic sequencer discovery for pathologically proven lung cancer detection. Journal of medical imaging 4, 041305-041305 (2017).

161 Du, W., He, B., Luo, X. & Chen, M. Diagnostic value of artificial intelligence based on CT image in benign and malignant pulmonary nodules. Journal of Oncology 2022, 5818423 (2022).

162 Nazir, I., Haq, I. u., AlQahtani, S. A., Jadoon, M. M. & Dahshan, M. Machine Learning‐Based Lung Cancer Detection Using Multiview Image Registration and Fusion. Journal of Sensors 2023, 6683438 (2023).

163 Pavel, M. A., Islam, R., Babor, S. B., Mehadi, R. & Khan, R. Non-small cell lung cancer detection through knowledge distillation approach with teaching assistant. PloS one 19, e0306441 (2024).

164 Lalitha, S. An automated lung cancer detection system based on machine learning algorithm. Journal of Intelligent & Fuzzy Systems 40, 6355-6364 (2021).

165 Alakwaa, W., Nassef, M. & Badr, A. Lung cancer detection and classification with 3D convolutional neural network (3D-CNN). International Journal of Advanced Computer Science and Applications 8 (2017).

166 Shakeel, P. M., Burhanuddin, M. A. & Desa, M. I. Lung cancer detection from CT image using improved profuse clustering and deep learning instantaneously trained neural networks. Measurement 145, 702-712 (2019).

167 Makaju, S., Prasad, P., Alsadoon, A., Singh, A. & Elchouemi, A. Lung cancer detection using CT scan images. Procedia Computer Science 125, 107-114 (2018).

168 Annemneedi Lakshmanarao, N. G., Nagagopiraju Vullam, Mandapati Sridhar, Modalavalasa Krishna Kanth, Uma Maheswari Rayudu. Lung cancer detection using hybrid integration of autoencoder feature extraction and ML techniques. Indonesian Journal of Electrical Engineering and Computer Science 37, 416~424 (2025).

169 Saha, C. et al. Lung-AttNet: An Attention Mechanism based CNN Architecture for Lung Cancer Detection with Federated Learning. IEEE Access (2025).

170 Sünnetci, K. M. & Alkan, A. Lung cancer detection by using probabilistic majority voting and optimization techniques. International Journal of Imaging Systems and Technology 32, 2049-2065 (2022).

171 un Nisa, Z., Jaffar, A., Bhatti, S. M. & Butt, U. M. Lung Cancer Detection using Segmented 3D Tensors and Support Vector Machines. International Journal of Advanced Computer Science and Applications 14 (2023).

172 ÇAY, T. Lung cancer diagnosis with GAN supported deep learning models. Bio-Medical Materials and Engineering, 09592989241308775 (2025).

173 Mahum, R. & Al-Salman, A. S. Lung-RetinaNet: Lung cancer detection using a RetinaNet with multi-scale feature fusion and context module. IEEE Access 11, 53850-53861 (2023).

174 Vishwa Kiran, S. et al. Machine learning with data science-enabled lung cancer diagnosis and classification using computed tomography images. International Journal of Image and Graphics 23, 2240002 (2023).

175 Ganashree TS, M. C., Mithileysh Sathiyanarayanan. Convolutional Neural Networks and AI Technology for Early Detection of Lung Cancer: A Deep Learning Approach. International Research Journal of Multidisciplinary Scope 6, 141-151 (2025).

176 Ji, Z. et al. ELCT-YOLO: An efficient one-stage model for automatic lung tumor detection based on CT images. Mathematics 11, 2344 (2023).

177 Karthikeyan, B. et al. Multimodal Feature Fusion Using Optimal Transfer Learning Approach for Lung Cancer Detection and Classification on CT Images. Full Length Article 12, 84-84 (2024).

178 Seyrek, F. B. & Yiğit, H. Diagnosis of lung cancer from computed tomography scans with deep learning methods. Journal of Universal Computer Science 30, 1089 (2024).

179 Mary, A. A. & Thanammal, K. BlockChain and Deep Learning with Dynamic Pattern Features for Lung Cancer Diagnosis. International Journal of Advanced Computer Science & Applications 15 (2024).

180 Venkatesh, C. & Bojja, P. Lung cancer detection using bio-inspired algorithm in CT scans and secure data transmission through IoT cloud. International Journal of Advanced Computer Science and Applications 11 (2020).

181 Lin, X. et al. Lung cancer and granuloma identification using a deep learning model to extract 3-dimensional radiomics features in CT imaging. Clinical Lung Cancer 22, e756-e766 (2021).

182 Tiwari, A. et al. Optimized ensemble of hybrid rnn-gan models for accurate and automated lung tumour detection from ct images. International Journal of Advanced Computer Science and Applications (IJACSA) 14 (2023).

183 Elnakib, A., Amer, H. M. & Abou-Chadi, F. E. Early lung cancer detection using deep learning optimization. (2020).

184 Anitha, V., Behera, N. R., Krishna, P. V., NamdeoraoJogekar, R. & Singh, K. Detection of Lung Cancer Using Optimal Hybrid Segmentation and Classification. International Journal of Computer Information Systems and Industrial Management Applications 15, 11-11 (2023).

185 Pawar, V. J. & Premchand, P. in Web Intelligence. 37-59 (SAGE Publications Sage UK: London, England).

186 Fan, W. et al. Diagnostic value of artificial intelligence based on computed tomography (CT) density in benign and malignant pulmonary nodules: a retrospective investigation. PeerJ 12, e16577 (2024).

187 Hunter, B. et al. A radiomics-based decision support tool improves lung cancer diagnosis in combination with the Herder score in large lung nodules. EBioMedicine 86 (2022).

188 Nair, S. S., Devi, V. M. & Bhasi, S. Enhanced lung cancer detection: Integrating improved random walker segmentation with artificial neural network and random forest classifier. Heliyon 10 (2024).

189 Qi, K. et al. Lung-PNet: an automated deep learning model for the diagnosis of invasive adenocarcinoma in pure ground-glass nodules on chest CT. American Journal of Roentgenology 222, e2329674 (2024).

190 Abid, M. M. N., Zia, T., Ghafoor, M. & Windridge, D. Multi-view convolutional recurrent neural networks for lung cancer nodule identification. Neurocomputing 453, 299-311 (2021).

191 Pagadala, P. K. et al. Enhancing Lung Cancer Detection from Lung CT Scan Using Image Processing and Deep Neural Networks. Revue d'Intelligence Artificielle 37, 1597 (2023).

192 Li, B., Gao, M. & Wang, T. The role of visual sensor-based CT imaging for rapid diagnosis of lung cancer markers in athletic patients. REVISTA INTERNACIONAL DE MEDICINA Y CIENCIAS DE LA ACTIVIDAD FISICA Y DEL DEPORTE 22, 318-335 (2022).

193 Asuntha, A. & Srinivasan, A. Deep learning for lung Cancer detection and classification. Multimedia Tools and Applications 79, 7731-7762 (2020).

194 Abdulqader, A. F. et al. Multi-objective deep learning for lung cancer detection in CT images: enhancements in tumor classification, localization, and diagnostic efficiency. Discover Oncology 16, 529 (2025).

195 Saha, A. et al. VER-Net: a hybrid transfer learning model for lung cancer detection using CT scan images. BMC medical imaging 24, 120 (2024).

196 Yang, H. et al. Diagnostic value of circulating genetically abnormal cells to support computed tomography for benign and malignant pulmonary nodules. BMC cancer 22, 382 (2022).

197 Ni, R. et al. An early lung cancer diagnosis model for non-smokers incorporating ct imaging analysis and circulating genetically abnormal cells (CACs). BMC cancer 25, 124 (2025).

198 Zhang, C. et al. Enhancing lung cancer diagnosis with data fusion and mobile edge computing using DenseNet and CNN. Journal of Cloud Computing 13, 91 (2024).

199 Shah, A. A., Malik, H. A. M., Muhammad, A., Alourani, A. & Butt, Z. A. Deep learning ensemble 2D CNN approach towards the detection of lung cancer. Scientific reports 13, 2987 (2023).

200 Alazwari, S. et al. Computer-aided diagnosis for lung cancer using waterwheel plant algorithm with deep learning. Scientific Reports 14, 20647 (2024).

201 Hammad, M. et al. Explainable AI for lung cancer detection via a custom CNN on CT images. Scientific Reports 15, 12707 (2025).

202 Hendrix, W. et al. Deep learning for the detection of benign and malignant pulmonary nodules in non-screening chest CT scans. Communications medicine 3, 156 (2023).

203 Güraksın, G. E. & Kayadibi, I. A Hybrid LECNN Architecture: A Computer-Assisted Early Diagnosis System for Lung Cancer Using CT Images. International Journal of Computational Intelligence Systems 18, 35 (2025).

204 Zheng, S. et al. Interpretative computer-aided lung cancer diagnosis: from radiology analysis to malignancy evaluation. Computer Methods and Programs in Biomedicine 210, 106363 (2021).

**Table S2** Technical details of methods developed for the detection and diagnosis of lung cancer.

| **First author (year)** | **Type/name of method** | **Dataset** | **Nature** | **Data pre-processing technique** | **Augmentation/optimization technique** | **Train/validation/test split methodology** | **Comparison** |
| --- | --- | --- | --- | --- | --- | --- | --- |
| Priya A (2024) [1] | EfficientNet | Cancer imaging archive (CIA) | Cohort size | Transition pre-training model | Modified Gravitational Searching algorithm | 75% for training, 25% for testing | Better performance compared to VGG-19, Inception-v3, ResNet 152, and SqueezeNet models; lowest performance compared to Inception-v3 and SqueezeNet models |
| Bushara A R (2023) [2] | Capsule neural network (CapsNet) | LIDC-IDRI | Public | ImageDataGenerator | Rescaling, rotation, and horizontal and vertical flips | 60% for training, 20% for testing, 20% for validation | Better performance compared to multi-corp CNN, 3D-MCN, CNN, MIXCAPS, CapsNet methods |
| Mattakoyya Aharonu (2024) [3] | Deep Convolutional cross max-out kernel graph-based Generative Adversarial Network with Enhanced Prism refraction Search (DCGAN-EPS) | LIDC-IDRI, Chest CT Scan, IQ-OTH/NCCD | Public | Adaptive wavelet denoising | Enhanced prism refraction search (EPS) algorithm | 60% for training, 20% for testing, 20% for validation | Better performance compared to GF-EDBN, EDL, DLCNN, UDCT, MobileNetV2-SGRU methods |
| Akila Agnes Sundaresan (2018) [4] | Transfer learning-based feed-forward deep neural network | LIDC-IRDI | Public | ‒ | Transfer learning approach | ‒ | Better performance compared to VGG16 model |
| Ahmed Alksas (2023) [5] | Hyper-tuned stacked generalization-based classification architecture | LIDC-IDRI | Public | Modified local ternary pattern | Near-optimal model | ‒ | Better performance compared to multi-level CNN, ensemble deep learning approach, 3D gray density coding feature, SHAP-based interpretation-model |
| Firas H Almukhtar (2024) [6] | Error correcting output codes (ECOC) | Al-Salam Hospital | Private | Binary operation | Social spider optimization (SSO) | ‒ | Better performance compared to deep learning with instantaneously trained neural networks (DITNN) approach, novel neural-network based algorithm, wavelet feature descriptor and feed forward back propagation neural networks classifier method |
| Mohammad H Alshayeji (2023) [7] | NoduleDiag | LIDC-IDRI | Public | Pylidc package, normalization, median filter | Adam and stochastic gradient descent with momentum (SGDM) optimizers | Ten train-test splits | Better performance compared to CNN based on Le-Net, Deep feature extractor (ResNet-50)+ SVM-RBF classifier, Extracted self-learned features with 8 layered CNN, Gaussian mixture model in region-based convolutional neural network methods |
| Sajeev Ram Arumugam (2024) [8] | Fractional honey badger golden search optimization algorithm (FHBGSO)-based Shepard convolutional neural networks (ShCNN) | LIDC-IDRI | Public | Adaptive wiener filter | Honey badger golden search optimization algorithm (HBGSO) | ‒ | Better performance compared to MLRPN, DL, DNN, and CNN methods |
| Abolfazl Bagheri Tofighi (2025) [9] | MobileNetV2-SGRU | Q-OTH/NCCD | Public | Resizing and normalization | Horizontal flip, rotation, bright-ness adjustment, zooming, width shift, and height shift operations | 80% for training, 20% for testing | Better performance compared to ResNet50-SGRU, VGG19-SGRU, VGG16-SGRU, DenseNet121-SGRU methods |
| Yang Bai (2022) [10] | High-resolution reconstruction model | CT database | Cohort | Denoising and image enhancement operations, histogram equalization processing | Translation, rotation, vertical flip, horizontal flip, and zoom operations | ‒ | ‒ |
| Prasanalakshmi Balaji (2023) [11] | Multi serial hybrid convolution based residual attention network (MSHCRAN) | Chest CT-Scan images Dataset | Public | Residual attention network | ‒ | 70% for training, 20% for testing | Better performance compared to CNN, ResNet, LSTM, CRAN methods |
| Ananya Bhattacharjee (2021) [12] | Feed forward neural network | LIDC-IDRI | Public | ROI selection | Gray values cumulative function | 90% for training, 10% for testing | Better performance compared to GLCM, deep features |
| Ananya Bhattacharjee (2022) [13] | Adaptive boost-based grid search optimized random forest (Ada-GridRF) | LIDC-IDRI | Public | Regionprops function | Grid search optimized random forest | 80% for training, 20% for testing | Better performance compared to spatial kernelized fuzzy c-means and ensemble learning, hyperparameter optimization of convolutional neural networks, segmented vessel tree and kernel method, convolutional neural network-based PSO |
| A R Bushara (2023) [14] | Visual geometry group - capsule network (VGG-CapsNet) | LIDC-IDRI, Kaggle | Public | Resizing and normalization | Rescaling, rotation, and horizontal and vertical flips operations | 60% for training, 30% for testing, 10% for validation | Better performance compared to CMixNet, mRFCN, 3D-MCN, M-CNN, Augmented CNN, DFD-Net, GoogLeNet, CapsNet, CNN-CapsNet methods |
| Venkatesh Chapala (2021) [15] | Internet of Things (IoT)-enabled platform | CT databases | Public, private | Median filter | Cuckoo search optimization | ‒ | Better performance compared to Hessan Matrix + SVM, FF + FFBPNN, ACO + SVM, FCM + Region growing, GA + SVM |
| Liuyin Chen (2024) [16] | Self-distillation trained multitask dense-attention network (SD-MdaNet) | NanFang Hospital | Private | ‒ | ‒ | ‒ | ‒ |
| Hussain Dawood (2025) [17] | CenterNet | LUNA-16, Kaggle | Public | ‒ | Attention mechanism | ‒ | Better performance compared to deep CNN, effective deep learning approach, AlexNet, hybrid deep learning methods |
| S G Devi (2024) [18] | Advanced machine learning algorithms | CT scan dataset | Private | ‒ | ‒ | ‒ | Better performance compared to traditional CNN |
| Ashit Kumar Dutta (2022) [19] | Random forest (RF) classifier with Convolutional Neural Network (CNN) | LIDC-IDRI, VIA/I-ELCAP | Public | Filtering technique | Optimization module | 70% for training, 30% for testing | ‒ |
| Veerraju Gampala (2024) [20] | Archimedes flow regime optimization (AFRO) –based deep belief network (DBN) | IDC-IDRI | Public | Wiener filter | Archimedes optimization algorithms (AOA) | 90% for training, 10% for testing | Better performance compared to CNN, SE-ResNeXt, 3D-CNN, WSLnO-based ShCNN |
| Nandita Gautam (2024) [21] | ResNet-152, DenseNet-169, and EfficientNet-B7 models | IDC-IDRI | Public | - | Weight optimization technique | 60% for training, 20% for testing, 20% for validation | Better performance compared to forward and backward GAN and multi-scale VGG16, Deep residual learning, resolved ambiguity local binary pattern, novel DCNN, LCD-CapsNet methods |
| Arash Heidari (2023) [22] | Federated learning and block chain systems | Cancer imaging archive (CIA), Kaggle data science bowl (KDSB), LUNA 16, local dataset | Public | Image histogram approach, weighted mean histogram equalization strategy, spatial normalization and signal Normalization methods | Adam optimizer | 35% for training, 35% for testing, 30% for validation | Better performance compared to CNN, DNN, Discriminative extreme learning machine, ResNet152V2 + GRU model, CNN + SVM + KNN |
| Hadeer A Helaly (2024) [23] | ELCD-NSC model | Cancer imaging archive (CIA), Kaggle dataset | Public | Autoencoder technique structure | Rotation and reflection techniques | 80% for training, 10% for testing, 10% for validation | Better performance compared to deep learning model, machine learning-based model, high ranking deep ensemble learning-based lung cancer diagnosis model (HRDEL) |
| Shengchao Hou (2022) [24] | U-Net-Newton’s method | LIDC-IDRI | Public | ‒ | ‒ | ‒ | Better performance compared to genetic algorithm-back propagation (GA-BP), random forest (RF), Semi-Naïve Bayesian (SNB), and filtered back projection (FBP) algorithms |
| Ratishchandra Huidrom (2022) [25] | Neuro-evolutional based computer aided detection system | LIDC-IDRI | Public | ‒ | Particle swarm optimization | 70% for training, 30% for testing | Better performance compared to MTANNs, ANN with fixed topology, Ensemble learning, Two-stage CNN, FLD, 2D-CNN, GA + PSO methods |
| Richa Jain (2024) [26] | DenseNet20, EfficientNetB7, VGG16, MobileNet and VGG19 models | IQ-OTH/NCCD | Public | Gaussian blur, normalization method | Elastic transformation, Adam (Adaptive Moment Estimation), Adagrad (Adaptive Gradient Descent), Adadelta, SGD (Stochastic Gradient Descent) and RMSprop (Root Mean Square Propagation) optimizers | 70% for training, 15% for testing, 15% for validation | Better performance compared to LungNet22, EfficientNet v2-M, VGG 19 + CNN, 7 Pre-trained CNN models, CNN and GoogleNet, AMPWSVM classifier |
| M Kanipriya (2022) [27] | Convolutional neural network (CNN) based long and short-term memory (LSTM) | LIDC-IDRI | Public | Binary operation | Improved Capuchin Search Algorithm (ICSA) | ‒ | Better performance compared to DCNN, SANet, CONVNet, Multilevel Particle Swarm Optimization (MPSO) optimized CNN (Hybrid MPSO-CNN), SVM, Rule-based filtering (RBF), k-means clustering and SVM, ensemble CNN, and particle swarm optimization (PSO) optimized Hybrid LSTM-CNN with ICSA optimized hybrid CNN-LSTM model |
| Mehr Kashyap (2025) (28) | Three-dimensional U-Net ensemble approach | Medical center datasets | Private | Lung region isolation model | Adam optimizer | 80% for training, 5% for testing, 15% for validation | Physician performance |
| Sadam Kavitha (2025) [29] | Multi-head attention-based fused depth wise convolutional neural network (MHA-DCNN) | IQ-OTH/NCCD | Public | Disperse wiener filter | Enhanced binary black widow optimization (EBWO), adaptive equilibrium optimization (AEO) | ‒ | Better performance compared to IDNN, DFDN, CFPSOA, AHHMM, NB-DT-SDS, D-ShrimpNet methods |
| Suhad Jasim Khalefa (2024) [30] | Finite element method-deep auto encoder algorithm- convolutional neural network (FEM-DCNN) | LIDC-IDRI | Public | Non-local means filter, wiener filter, and median filter | Transformation methods such as cropping, flipping and rotating | ‒ | Better performance compared to single CNN approach |
| Geethu Lakshmi G (2025) [31] | Squeeze-Inception-ResNeXt model | Kaggle dataset | Public | Color space conversion, resizing, and normalization methods | Slime Mould algorithm (SMA) | 80% for training, 20% for testing | Better performance compared to CNN, SNN, RetinaNet, Hybrid SqueezeNet-Inception V3, VER-Net |
| Madhusudan G Lanjewar (2023) [32] | DenseNet | Kaggle dataset | Public | Resizing, and normalization methods | Adam optimizer | 90% for training, 10% for testing | Better performance compared to SVM with GWO-GA, CNN, CNN, DNN, and SAE, BPNN, U-Net and 3D multipath methods |
| Zhengrong J Liang (2025) [33] | Machine learning (ML) models | LdCT dataset | Private | Affine transformation | ‒ | 80% for training, 20% for testing | Better performance compared to RF-HFs, 3D-DL-Clinic, 3D-DL-Public, 3D-DL-IRIS models |
| Lei Ma (2024) [34] | GoogLeNet with Adaptive Layers (GoogLeNet-AL) | Q-OTH/NCCD, chest CT-scan image dataset | Public | Resizing, and normalization methods | Rotation, scaling, flipping, and translation operations | ‒ | Better performance compared to EESNN-FSOA, LCGAN, SENET, T-GAN, ECNN methods |
| Selma Mammeri (2024) [35] | YOLO v7 | LIDC-IDRI | Public | Median and adaptive median filters | ‒ | 70% for training, 20% for testing, 10% for validation | Better performance compared to YOLO v3, YOLO V5, Faster RCNN, YOLO v5 algorithms |
| Suguna Mariappan (2024) [36] | Hybrid efficient-ShuffleNet (HES-Net) method | LIDC-IDRI | Public | Bilateral filter | FC minimalizing method | 90% for training, 10% for testing | Better performance compared to DFD-Net, CNN+SVM, TWED LNN, Deep ensemble 2D CNN techniques |
| B Muthazhagan (2023) [37] | Sooty-LuCaNet | LIDC-IDRI | Public | Gaussian filter, contrast stretching histogram equalization | Sooty tern optimization algorithm | 75% for training, 25% for testing | Better performance compared to Alex Net, Google Net, Res Net, Mobile Net, Shuffle Net methods |
| Potti Nagaraja (2023) [38] | Ensemble-based deep learning model (EDLM) | Chest CT-Scan images | Public | Weighted mean histogram equalization and mean filte | Improved Harris Hawks Optimization (IHHO) | 70% for training, 10% for validation, 20% for testing | Better performance compared to DHOA-EDLM, TSA-EDLM, SSA-EDLM, HHO- EDLM algorithms |
| M Navaneethakrishnan (2023) [39] | Bat deer hunting optimization algorithm-based deep convolutional neural network (BDHOA-based DCNN) | LIDC-IDRI | Public | Median filter | Bat deer hunting optimization algorithm, random erasing and cropping model | 90% for training, 10% for testing | Better performance compared to Segmentation + Deep CNN, NN, Optical flow model, deep learning methods |
| Neslihan Ozcelik (2024) [40] | CNNs model | Recep Tayyip Erdogan University Chest Disease clinic | Private | Moving average method- fastest blur algorithm | Adamax optimizer | 80% for training and 20% for testing | - |
| Yong-Jin Park (2021) [41] | Deep transfer learning (TL) models | Chest CT dataset | Cohort | ‒ | Horizontal and vertical flips, random rotation methods, stochastic gradient descent optimizer | ‒ | ‒ |
| C S Parvathy (2024) [42] | Squeeze and excitation convolutional neural networks (SENET) | IQ-OTH/NCCD | Public | Adaptive bilateral filter (ABF), reformed histogram equalization (RHE) | Tuna Swam optimization algorithm | 80% for training, 20% for testing | Better performance compared to DenseNet-121, 3D-CNN, lung cancer prediction convolutional neural network (LCP-CNN), adaptive hierarchical heuristic mathematical model (AHHMM) |
| Mohamed Shakeel Pethuraj (2023) [43] | Butterfly optimization algorithm-based k-means clustering (BOAKMC) algorithm | Cancer imaging archive (CIA) | Public | Mean filter and histogram equalization | Butterfly optimization algorithm, supervised Jaya optimized rough set related feature selection (SJORSFS) | 60% for training, 40% for testing | Better performance compared to region growing (RG), global thresholding (GT), fuzzy c-means (FCM), canny segmentation (CS), Sobel segmentation (SS) methods |
| Umesh Prasad (2024) [44] | Spotted hyena optimization with seagull algorithm | LIDC-IDRI | Public | Bicubic-interpolation (BI) technique, Gaussian, guided filter, median filter, top and bottom hat, adaptive histogram equalization | Spotted hyena optimization with seagull optimization algorithm (SH-SOA), deep convolutional generative adversarial networks (DCGAN) | 80% for training, 20% for testing | Better performance compared to ODNN, EBT, KNN, optimized 2DCNN, IDNN methods |
| Syeda Reeha Quasar (2024) [45] | BEiT, DenseNet, AND, OR, weighted box fusion, and boosting models | Kaggle dataset | Public | Noise reduction, contrast adjustment, brightness leveling methods | resizing and cropping methods | 80% for training, 10% for validation, 10% for testing | Better performance compared to ResNet50, InceptionV3, DenseNet121, Xception, SE-ResNet50, DenseNet201, NASNet, Sequential CNN, VGG16, decision tree models |
| N Rathan (2024) [46] | High-resolution net (HRNet) | LIDC-IDRI | Public | ‒ | ‒ | 85% for training, 15% for testing | Better performance compared to BPSO-DT, CNN and RNN, 3D CNN, 3D CNN-AlexNet, DenseNet methods |
| Amjad Rehman (2023) [47] | Convolution neural network (CNN) algorithm | LIDC-IDRI | Public | Gaussian filter, Sobel filter | ‒ | ‒ | Better performance compared to Toboggan, RF+fCRF, Unet, segnet, FRRN methods |
| Mohammad Hosein Sabzalian (2023) (48) | Bidirectional recurrent neural network (BRNN) | IQ-OTH/NCCD, Lung-PET-CT-Dx | Public | Noise cancellation, contrast enhancement | SdSmote technique, Ebola optimization search algorithm | ‒ | Better performance compared to minimum redundancy maximum relevance feature selection (MRMRFS) method, Co-learning feature fusion maps (CO-l), probability of malignancy calculation (PMC), wavelet feature descriptor (Wavelet-NN) and FNN classifier, and MC-SVM classifier |
| Nishat Shaikh (2023) [49] | Modified dimension range-based cat swarm optimization (MDR-CSO) | Chest CT-scan images dataset, medical segmentation decathlon | Public | Frangi filter, median filter, CLAHE technique | Cat swarm optimization | 70% for training, 30% for testing | Better performance compared to TSA-ERNN, DHOA-ERNN, CSO-ERNN, SSA-ERNN methods |
| P Mohamed Shakeel (2022) [50] | Spiral optimization intelligent-generalized rough set approach | Cancer imaging archive (CIA) | Cohort size | Multilevel brightness-preserving approach | Hybrid spiral optimization intelligent-generalized rough set approach | 70% for training, 30% for testing | Better performance compared to wrapper approach (GAWA), particle swarm optimization-based multiobjective selection (PSOMS) and ant colony optimization (ACO) methods |
| Malayil Shanid (2021) [51] | Adaptive-SALP-elephant herding optimization algorithm (adaptive-SEOA) - deep belief network (adaptive-SEOA-DBN) | LIDC-IDRI | Public | Noise removing method | Salp-elephant herding optimization algorithm | 90% for training, 10% for testing | Better performance compared to fuzzy C-means (MSFCM), DFCNet, deep belief network (DBN), hybrid geometric active contour methods |
| Zhiqiang Shen (2023) [52] | Weakly-supervised lung cancer detection and diagnosis network (WS-LungNet) | LIDC-IDRI | Public | Intensity clip and normalization methods | Adam optimizer, random scale, random crop, random horizontal flip, vertical flip | ‒ | Better performance compared to nodule-ResNet, MV-KBC, TurmorNet, PN-SAMP, local-global networks, MSCS-DeepLN methods |
| Feng Shi (2021) [53] | Semi-supervised deep transfer learning (SDTL) | University datasets | Cohort | Trilinear interpolation method, Z-score standardization method | Random flipping, rotation, and scaling methods | 80% for training, 20% for testing | ‒ |
| Ebtasam Ahmad Siddiqui (2023) [54] | Enhanced deep belief network (E-DBN) | LIDC-IDRI, LUNA 16 | Public | Gabor filter | Adam optimizer | 50% for training, 50% for testing | Better performance compared to wavelet feature descriptor and support vector machine, optimal deep belief network with opposition-based hybrid grasshopper and honeybee optimization algorithm, optimal deep learning model |
| Amit Singh (2024) [55] | VGG16 | Kaggle | Public | Filters | ‒ | 70% for training, 30% for testing | Better performance compared to transfer learning, hybrid deep learning, SVM & Navie Bayes classifiers, deep ensemble 2D-CNN methods |
| Prasath Sivasankaran (2024) [56] | Deep belief networks (DBN) stacked Denotion Autoencoders (SDAE) | LIDC-IDRI | Public | Anisotropic diffusion filter (ADF) | ‒ | ‒ | Better performance compared to DBN, CNN methods |
| S Sridevi (2024) [57] | 3D Trans-DenseUnet++ with Novel Loss Function (3D-TD ++-NLF) | Kaggle | Public | ‒ | ‒ | ‒ | Better performance compared to LSTM, RNN, ResNet, DenseNet methods |
| R Sudha (2024) [58] | Hybrid particle snake swarm optimization (PS2OA) | LIDC-IDRI | Public | Adaptive right median filter (AMF), contrast limited adaptive histogram equalization (CLAHE) | Hybrid particle snake swarm optimization (PS2 OA) | ‒ | Better performance compared to MMEL-3DCNN, CNN, Texture CNN, BCNN methods |
| R Sudha (2025) [59] | Adaptive pelican optimization with optimized mask region-based convolutional neural network (R-CNN) | LIDC-IDRI | Public | Gaussian filter | Adaptive pelican optimization (APO) algorithm | 80% for training, 20% for testing | Better performance compared to MMEL-3DCNN, CNN, Texture CNN, BCNN |
| C Usharani (2025) [60] | Federated learning with convolution neural network (ResNet50) | IQ-OTH/NCCD | Public | ‒ | ‒ | 75% for training, 25% for testing | Better performance compared to CNN optimized and improved Snake optimization algorithm, KNN, CFM, WSDL, CLFM, DFD-Net methods |
| Lakshmana Rao Vadala (2025) [61] | SpiLenet | LIDC-IDRI | Public | Savitzky-Golay (SG) filter | ‒ | 90% for training, 10% for testing | Better performance compared to improved DNN, DGMM-RBCNN, DCNN, HSEFS methods |
| N Velmurugan (2025) [62] | Rat swarm political optimizer (RSPO) based Shepard convolutional neural networks (ShCNN) (RSPO_ShCNN) | LIDC-IDRI, Medical Deepfakes: lung cancer dataset | Public | Laplacian filter | Rat swarm political optimizer (RSPO) | 90% for training, 10% for testing | Better performance compared to mRFCN, KNG-CNN, FFBP-NN, ANN methods |
| C Venkatesh (2022) [63] | Dynamic optimization and deep learning technique | Laboratory dataset | Private | Median filter | Cuckoo-search optimization algorithm, dynamic particle swarm optimization method | ‒ | Better performance compared to existing methods |
| Guangxi Wang (2022) [64] | Lung cancer artificial intelligence detector | Hospital dataset | Cohort | L2 normalization method | ‒ | 75% for training, 25% for testing | ‒ |
| Meng Wang (2025) [65] | 3D auto-encoder networks | CT scan dataset | Private | Resizing and normalization methods | random rotation, horizontal flipping, random scaling methods, Adam optimizer | 70% for training, 15% for validation, 15% for testing | Better performance compared to existing models |
| Niyaz Ahmad Wani (2024) [66] | DeepXplainer | Survey lung cancer (SLC) | Public | Filters | Adam optimizer | ‒ | Better performance compared to support vector machine (SVM), ANN, ANN-based classification system methods |
| Yifei Xu (2022) [67] | YOLOv3 network | LIDC-IDRI, Hospital dataset | Public, private | Maximum interclass variance method | ‒ | 70% for training, 30% for testing | Better performance compared to CNN, DNN, SAE, DTCNN-ELM, MC-CNN, Multi-view CNN, ANN, ML-xResNet methods |
| Yeguo Xu (2022) [68] | Alexnet optimized by modified Bowerbird optimization algorithm | Cancer imaging archive (CIA) | Public | Wiener filter | Satin bowerbird optimization algorithm | 80% for training, 20% for validation | Better performance compared to KNN, RF, SVM, MLP, ANN-TEO methods |
| Chaohua Yan (2023) [69] | CNN model optimized by Snake optimization algorithm | IQ-OTH/NCCD | Public | Median filter, contrast enhancement | SMOTE technique, improved snake optimization algorithm | 75% for training, 25% for testing | Better performance compared to k-Nearest neighbors (KNN), integrated contrast /features-based method (CFM), weakly supervised deep learning (WSDL), Co-learning feature fusion maps (CLFM), and DFD net |
| Runhuang Yang (2024) [70] | Multimodal integrated feature neural network (MIFNN) | LUNA16, specialized center dataset | Public, private | ‒ | ‒ | ‒ | Better performance compared to existing models |
| Sufyan Othman Zaben (2024) [71] | Convolution neural networks (CNN) model | Cancer imaging archive (CIA) | Public | Histogram equalization, resizing and normalization methods | Adam optimizer | 33% for training, 67% for testing | Better performance compared to SVM with GWO-GA, deep CNN, SAE, DNN, CNN, Feed-forward BPNN, U-Net, 3D VGG-like network methods |
| Qinghe Zheng (2025) [72] | Reconstruction error based implicit regularization method (REbIRM) | DSB 2017, TCGA-LUAD, RIDER Lung CT | Public | Resizing and normalization methods | Transformation methods such as cropping, flipping and rotating | 80% for training, 20% for testing | Better performance compared to DisturbLabel, SoftLabel, NoiseLayer, Distillation, Dropout, DropConnect methods |
| A Angel Mary (2024) [73] | Novel deep learning-based lung cancer detection (DL-LCD) model | LIDCIDRI | Public | Contrast limited adaptive histogram equalization (CLAHE) | Global optimization algorithm, adaptive contrast augmentation technique | ‒ | Better performance compared to CNN, FPSOCNN, ODNN methods |
| Shalini Wankhade (2023) [74] | Cancer cell detection using hybrid neural network (CCDCHNN) | LUNA 16 | Public | Convolutional filter | Adam optimizer | 80% for training, 20% for validation | Better performance compared to 3D-CNN, RNN, DNN methods |
| Juyoung Lee (2023) [75] | IDCal model | University dataset | Cohort | Resizing and normalization methods | Adam optimizer | 80% for training, 20% for validation | Physician performance |
| Ju Hwan Lee (2025) [76] | Unsupervised anomaly detection (UAD) | Samsung medical center (SMC) dataset, LIDC-IDRI, Cancer imaging archive (TCIA) | Private, public | Resizing and normalization methods | Adam optimizer | 60% for training, 40% for testing | Better performance compared to pDDPM, mDDPM, G-UADL, SL methods |
| Anum Masood (2018) [77] | DFCNet | Hospital dataset | Private | Resizing and normalization methods | Stochastic Gradient Descent (SGD), batch gradient descent (BGD) | 60% for training, 40% for testing | Better performance compared to CNN, DFCNet, TumorNet methods |
| Yunzeng Zhang (2025) [78] | Multi-omics model | DICOM, Hospital dataset | Public, private | ‒ | Optimization model | ‒ | Better performance compared to metabonomic, radiomics models |
| Jayapradha J (2025) [79] | Enhanced Integrated model for Lung Tumor Identification (EIM-LTI) | IQ-OTH/NCCD | Public | Haar and Gabor filters, sharpening and cropping methods | Convolutional neural network, enhanced integrated model, Adam optimizer | 80% for training, 20% for testing | Better performance compared to CNN, SVM, 3D-CNN methods |
| Mohamed Hammad (2024) [80] | Convolutional neural networks+ layers with long short-term memory (CNN+LSTM) | Kaggle | Public | Resizing and normalization methods, linear discriminant analysis (LDA) | Tree-based pipeline optimization tool (TPOT), modified gravitational search algorithm (MGSA) | 80% for training, 20% for testing | Better performance compared to CNN+LDA+MGSA, CNN+IPCT, CNN+SVM, Deep Ensemble 2D CNN, 3D-VNet +3D-ResNet, VER-Net, STN +VGG-16 +CNN methods |
| R. Pandian (2022) [81] | CNN and Google Net deep learning algorithms | Hospital dataset | Private | ‒ | ‒ | 70% for training, 30% for validation | ‒ |
| Mohammad Q Shatnawi (2025) [82] | Enhanced CNN model | IQ-OTH/NCCD | Public | Histogram equalization (HE), contrast-limited adaptive histogram equalization (CLAHE), median filter (MF) | Shear range, horizontal flip, vertical flip methods, Adam optimizer | 70% for training, 10% for validation, 20% for testing | Better performance compared to ConvNeXt, VGG16, ResNet50, IneseptionV3, EfficientNetB0 methods |
| A A Abe (2025) [83] | DeepNodule-Detect | IQ-OTH/NCCD | Public | ‒ | Vertical flip, horizontal flip, image rotation methods, Adam optimizer | 80% for training, 20% for testing | Better performance compared to GoogLeNet, SVM, CNN methods |
| Michail Tsivgoulis (2022) [84] | SqueezeNet model | LUNA16 | Public | Resizing and normalization methods | Generative adversarial networks (GAN), symmetric augmentation method | ‒ | Better performance compared to light CNN, ShuffleNet, mobile Net v2, LeNet-50, DenseNet-121, ResNet-50, VGG-11 methods |
| Negar Maleki (2023) [85] | Gradient Boosting (GB), Random Forest (RF), Support Vector Machine (SVM) models | Hospital dataset | Private | Resizing and denoising methods, Gabor filter, Sobel filter, Scharr filter, Prewitt filter, Gaussian filter | ‒ | 90% for training, 10% for testing | Better performance compared to CNN, ANN methods |
| Masahiro Yanagawa (2020) [86] | Three-dimensional convolutional neural network (3D-CNN) | Hospital dataset | Cohort | Normalization method | Flip method, Adam optimizer | 80% for training, 10% for validation, 10% for testing | Radiologist performance |
| Mohammad Alamgeer (2022) [87] | DLCADLC-BCT technique | Lung cancer dataset | Private | ‒ | Moth swarm optimization (MSO) algorithm | 70% for training, 30% for testing | Better performance compared to optimal DNN, RBF, LDC, KNN, DNN models |
| Luís Goncçalves (2018) [88] | CAD system | LIDC–IDRI | Public | Gabor filter | ‒ | 50% for training, 50% for testing | Radiologist performance |
| Mesut Togaçar (2019) [89] | Convolutional neural networks & minimum redundancy maximum relevance (CNN & mRMR) | Cancer imaging archive | Public | Resizing method | Stochastic gradient descent (SGD), RMSProp, Adam optimizers | ‒ | Better performance compared to GLCM, Deep CNN, CNN & Deep CNN & SAE, CNN & Multiview-CNN, CNN & Handcrafted methods & non-Handcrafted methods, CNN & Handcrafted methods & augmentation method |
| Kaiqiang Yang (2020) [90] | U-Net integrated with capsule networks (CapNets) | Hospital dataset | Cohort | Medical image labeling software | Rotation, folding, scaling and exchange methods | ‒ | Radiologist performance |
| Ayomide Abe (2025) [91] | AlexNet, ResNet-50, GoogLeNet models | IQ_OTH/NCCD, Kaggle | Public | Resizing method | Random method, Adam optimizer | 70% for training, 30% for testing | Better performance compared to transfer learning with GoogLeNet, CNN based model |
| Sreedar Bhukya (2025) [92] | Learning-based method for lung cancer detection (LbM-LCD) | LUNA-16 | Public | Resizing method | Rotation, flip, zoom, shear and centre shift methods, Adam optimizer | 80% for training, 20% for testing | Better performance compared to CNN, ANN models |
| Jenita Subash (2023) [93] | Oppositional based chimp optimization algorithm (OChOA) recurrent neural network (RNN) | IQ_OTH/NCCD | Public | Block matching and 3D filtering (BM3D), adaptive bilateral filter (ABF) | Chimp optimization algorithm (OChOA), social spider optimization (SSO), particle swarm optimization (PSO) | ‒ | Better performance compared to GA-RNN, PSO-RNN, SSO-RNN methods |
| Krmanj F (2024) [94] | Mask-RCNN | Hospital dataset | Private | Resizing method | Rotating, flipping, and scaling methods | 70% for training, 20% for validation, and 10% for testing | Radiologist performance |
| Chitra Thangavel (2023) [95] | Decked dragonfly optimization- speculative deceptive network (DDO-SDN) | EL-CAP | Public | Tanh normalization technique | Decked Dragonfly optimization (DDO) technique |  | Better performance compared to decision tree, KNN, GA-KNN methods |
| J Maruthi Nagendra Prasad (2022) [96] | Fuzzy K-means algorithm | LIDC–IDRI | Public | Wiener filter, blurring filter | Crow search optimization algorithm | ‒ | Better performance compared to inception-V3, Incep.ResNet-V2, GoogleNet, ResNet-101, ResNet-18 methods |
| Abdalbasit Mohammed Qadir (2024) [97] | Hybrid lung cancer stage classifier and diagnosis model (hybrid-LCSCDM) | IQ_OTH/NCCD | Public | Resizing and normalization methods | Stochastic gradient descent (SGD), RMSProp, Adam optimizers | ‒ | Better performance compared to GoogLeNet, SVM, AlexNet methods |
| Bassant Mostafa (2024) [98] | Convolutional neural networks -bi-directional long short-term memory (CNN-Bi LSTM) model | IQ_OTH/NCCD | Public | Median filter, resizing, shuffling, and normalization methods | Rotation, random, and flip methods | 80% for training, 20% for testing | Better performance compared to GoogleNet, VGG-16, AlexNet, DenseNet, R2MNet methods |
| Onur Ozdemir (2019) [99] | 3D probabilistic deep learning system | LUNA16, LIDC-IDRI | Public | Clipping and normalization methods, Gaussian filter | Affine transform augmentation method | ‒ | ‒ |
| Nabila Elloumi (2023) [100] | U-NET convolution network architecture | Hospital dataset | Private | Syngovia software | Adam optimizer | ‒ | Better performance compared to 3D-Unet, 3D-CNN methods |
| Shanchen Pang (2019) [101] | Densely connected convolutional networks and adaptive boosting | Hospital dataset | Private | Histogram equalization method | Rotation, translation and transformation methods | 70% for training, 30% for testing | Better performance compared to VGG-16, AlexNet, DenseNet, ResNet methods |
| Liu chenyang (2020) [102] | Joint nodule segmentation/recognition (JNSC) network | LUNA16, LIDC-IDRI | Public | Clipping and normalization methods | Adam optimizer | ‒ | Better performance compared to MC-CNN, Fuse-TSD, TMME, 2D-MV-KBC, 3D-MV-KBC methods |
| Bhavani Krishna (2024) [103] | Mask region-based convolutional neural network (Mask-RCNN) | LUNA16, LIDC-IDRI | Public | Resizing method | Smudge optimizer | 80% for training, 20% for testing | Better performance compared to general mask RCNN |
| Shuyin Duan (2020) [104] | Artificial neural network (ANN-3) | Hospital dataset | Private | ‒ | ‒ | ‒ | Better performance compared to C5.0-1, ANN-1, SVM-1, C5.0-2, ANN-2, SVM-2 models |
| Shalini Chowdary (2024) [105] | Transfer operator-based archimedes optimization - advanced dilated ensemble convolutional neural networks (MTO-AO- ADECNN) | Data science bowl 2017 lung cancer detection (DSB3), chest CT-scan images | Public | Normalization method | Mine blast optimization (MBO), Chameleon swarm optimization (CSO), black widow optimization (BWO) | ‒ | Better performance compared to MBO-ADECNN, CSO-ADECNN, BWO-ADECNN, AOA-ADECNN methods |
| Goran Jakimovski (2019) [106] | Double convolutional deep neural network (CDNN) | LONI | Public | ‒ | ‒ | 90% for training, 10% for testing | Better performance compared to regular CDNN |
| Burhanettin Ozdemir (2025) [107] | Attention enhanced inception next-based hybrid deep learning model | IQ-OTH/NCCD, Kaggle | Public | Resizing method | SGD optimizer, cropping, rotation, translation, scaling, and random noise methods | 60% for training, 10% for validation, and 30% for testing | Better performance compared to ResNet50, DensNet169, EffiecientNetv2-medium, ConvNeXt-base methods |
| Yossra Hussain Ali (2023) [108] | Particle swarm optimization (PSO) algorithm | LISS, LIDC‑IDRI | Public | Bayesian threshold‑based Taylor series approach, normalization method | Particle swarm optimization method | 80% for training, 20% for testing | Better performance compared to support vector machine (SVM), probabilistic neural network (PNN), conventional CNN methods |
| Sotiris Raptis (2024) [109] | DenseNet-201 CNN, XGBoost models | CT-DICOM | Public | Resizing and normalization methods | Random rotations, flipping, cropping, and scaling method | 60% for training, 20% for validation, and 30% for testing | Better performance compared to Grad-CAM, Local Interpretable Model-agnostic explanations (LIME), decision trees/rule-based models |
| Ailing Liu (2020) [110] | Radiomics nomogram model | Hospital dataset | Private | Gaussian filter, Wavelet filter | ‒ | 70% for training, 30% for validation | Radiologist performance |
| Imran Shafi (2022) [111] | Deep learning-assisted SVM-based model | LUNA16 | Public | Deep learning-based capsule network CapsNet method | ‒ | 80% for training, 10% for validation, and 10% for testing | Better performance compared to Watershed segmentation, KNN, NB, SVM, CNN, Google Net, DCNN, GBM + 3D CNN methods |
| Martina Sollini (2023) [112] | Computer-aided diagnosis (CADx) module | LUNA 16, ICH_s1 and ICH_s2 | Public | Neural network preprocessor | Adam optimizer, random rotations, flipping, and brightness variation methods | 70% for training, 20% for validation, and 10% for testing | ‒ |
| Mohammad Alamgeer (2023) [113] | Dung beetle optimization modified deep feature fusion model for lung cancer detection and classification (DBOMDFF-LCC) technique | Lungdb | Public | ‒ | Dung beetle optimization method | 70% for training, 30% for testing, 80% for training, 20% for testing | Better performance compared to ODNN, KNN, DNN, YOLO-DLN, DBN-LND, AGFLCC-DGM methods |
| Anum Masood (2019) [114] | Cloud-based 3D deep convolutional neural network (3DDCNN) | LUNA16, ANODE09, LIDC-IDR | Public | Multi-scale ROI patches, multi-angle ROI patches, multi-view combination | Cropping, duplicating, random translation methods, Adam optimizer | ‒ | Better performance compared to CNN, MTANNs, FCN, MC-CNN methods |
| Ling Zhang (2023) [115] | Radiomics model | Hospital dataset | Cohort | ‒ | ‒ | 70% for training, 30% for testing | - |
| Guanghui Song (2024) [116] | Two-dimensional sequence detection model based on a ConvLSTM (Convolutional Long Short-Term Memory) network and U-shaped structure network | LIDC-IDRI, LUNA16 | Public | Preprocessing methods | Stochastic gradient descent (SGD) algorithm | 60% for training, 40% for testing | Better performance compared to 3D RPN, NoduleNet methods |
| Mohd Munazzer Ansari (2025) [117] | SVMVGGNet-16 | LIDC-IDRI | Public | Median filter, histogram equalization, thresholding, edge detection | AI-augmented method | 70% for training, 10% for validation, and 20% for testing | Better performance compared to SVM, VGGNet-16 methods |
| Mohammad A Alzubaidi (2021) [118] | Support vector machine -histogram of oriented gradients (SVM-HOG) | TCIA | Public | Gabor filter, entropy filter, Warping and cropping methods | ‒ | ‒ | Better performance compared to traditional global approach |
| Xiao Zhang (2024) [119] | Deep convolutional neural network (DCNN) model | LUNGx, LIDC-IDRI | Public | Resizing and normalization methods | random spatial transformations and data augmentation methods | ‒ | Better performance compared to ResNet-50, InceptionV3, Xception methods |
| Nevin Aydın (2021) [120] | Deep CNN methods | Hospital dataset | Cohort | ‒ | ‒ | 80% for training, 10% for validation, and 10% for testing | Deep ensemble of convolutional and bidirectional recurrent neural network model |
| Vinit Kumar Gunjan (2022) [121] | Grey wolf optimization algorithm and recurrent neural network (GWO + RNN) | Kaggle | Public | Median filter | Grey wolf optimization algorithm, particle swarm optimization (PSO) | ‒ | Better performance compared to PSO + RNN, GA + RNN methods |
| Worku J Sori (2021) [122] | Two-path convolutional neural network (DFD-Net) | Kaggle Data Science Bowl 2017 challenge (KDSB), LUNA 16 | Public | Residual learning denoising model (DR-Net) | ‒ | ‒ | Better performance compared to DR-Net + DCA, deep learning, deep convolutional neural networks, convolutional neural networks models |
| Hye Soo Cho (2025) [123] | Artificial intelligence system | Hospital dataset | Cohort | ‒ | ‒ | ‒ | Radiologist performance |
| Nitha V R (2023) [124] | ExtRanFS | IQ-OTH/NCCD | Public | Resizing and normalization methods | Adam, Adagrad, Adadelta, RMSprop Optimizers | 80% for training, 20% for testing | Better performance compared to Xception + MLP, MobileNetV2+MLP, InceptionV3+MLP methods |
| Chia Chi Hsiao (2023) [125] | Machine learning-based method | Hospital dataset | Private | Isotropic voxel normalization | Gaussian Bayesian optimization method | 80% for training, 20% for testing | Better performance compared to deep learning optimized by marine predators’ algorithm, 3D CNNs, multi-crop convolutional neural networks, 2-pathway convolutional neural network methods |
| Isha Bhatia (2024) [126] | Lightweight advanced deep neural network (DNN) model | LUNA16 | Public | Ricker wavelet iterative center weighted median filter (RWICWM), histogram equalization technique, Resizing and normalization methods | Adam and RMSprop optimizers | 70% for training, 20% for validation, and 10% for testing | Better performance compared to UNet + WDSI-LSO, InceptionV3 + RWICWM, 3D-CNN + K-means + WDSI-LSO, Hybrid CNN-RNN + RWICWM + PLCOm 2012 methods |
| Amira Bouamrane (2024) [127] | Gradient-weighted class activation mapping (Grad-CAM) | LIDC-IDRI | Public | Converting and resizing methods | Mixup augmentation algorithm | 50% for training, 25% for validation, and 25% for testing | Better performance compared to BiCFormer, gradient boosting machine, DFF-CON, DenseNet-121 and SVM, SVM, LR, RF, DT, GNB, KNN methods |
| Rajeev Dixit (2025) [128] | Attention + Cross-Average Pooling | LIDC-IDRI | Public | Resizing method | Rotation, flipping, scaling methods, stochastic gradient descent (SGD) and Adam optimizers | ‒ | Better performance compared to traditional CNN |
| Rana M Mahmoud (2024) [129] | MobileNet model | LUNA16, LIDC-IDRI | Public | Normalization method, filters | SGD optimizer | 90% for training, 10% for testing | Better performance compared to CDP-ResNet, CA-Net, ALIAS, SANet, Mask R-CNN, 3D-MSViT, 3D SK-ResNet methods |
| Daisuke Yamada (2024) [130] | Multimodal hybrid model | Hospital dataset | Cohort | Normalization method | Random intensity shift, random rotation, random flip, random zoom, random affine transform, cropping methods | 50% for training, 30% for validation, and 20% for testing | Better performance compared to conventional radiomics models |
| Kavitha Belegere Chandraiah (2024) [131] | Convolutional neural network model | LIDC-IDRI | Public | Batch normalization | ‒ | 70% for training, 20% for validation, and 10% for testing | Better performance compared to VGG-16, Alex-Net methods |
| Sneha S Nair (2024) [132] | Artificial neural network (ANN) model | LIDC-IDRI | Public | Median filter, thresholding | ‒ | 80% for training, 20% for testing | Better performance compared to combinational features, hybrid model, Radiomics + CNN, multi-feature CNN methods |
| Venkata Tulasiramu Ponnada (2019) [133] | Efficient CNN (EFFI-CNN) | LIDC-IDRI | Public | Filters | ‒ | ‒ | Better performance compared to ICDSSPLD-CNN, EASPLD-CNN methods |
| Aswathy S U (2022) [134] | Bag of visual words-convolutional recurrent neural network model (BoVW–CRNN) | LIDC-IDRI | Public | Gabor filter, modified color-based histogram equalization | Guaranteed convergence particle swarm optimization (GCPSO) algorithm | 85% for training, 15% for testing | Better performance compared to Scheme & SVM with BOV Classifier, deep belief network and multiple classifiers, principal component analysis and SVM methods |
| Alaa Wehbe (2024) [135] | YOLOv8 small model | Lung PET-CT-DX, Lung3 | Public | ‒ | Adding Gaussian noise, rotation methods | 70% for training, 30% for testing | Better performance compared to faster-RCNN, YOLOv6, YOLOv7, YOLOv5 small methods |
| Nahed Tawfik (2024) [136] | Xception model | Kaggle | Public | Contrast limited adaptive histogram equalization (CLAHE) algorithm | CutMix data augmentation technique | 70% for training, 10% for validation, and 20% for testing | Compared to inception without CLAHE, inception with CLAHE, Xception without CLAHE, Xception with CLAHE, ResNet101 without CLAHE, ResNet101 with CLAHE |
| Suseela Triveni Vemula (2024) [137] | Convolutional neural network (CNN) model | Cancer imaging archive | Public | Normalization method, filters | Rotation, flipping, and scaling methods | ‒ | Better performance compared to VGG-16, VGG-19 methods |
| Sayana Sharma (2019) [138] | Convolutional neural network (CNN) model | CT scan image | Private | Gabor filter, image histogram equalization | Gradient descent optimizer | ‒ | Better performance compared to SVM method |
| Ananya Bhattacharjee (2023) [139] | Modified Xception model | IQ-OTH/NCCD | Public | Resizing and normalization methods | Adam optimizer | 80% for training, 20% for testing | Better performance compared to inception ResNet V2, inception V3, MobileNet V3 Small methods |
| Shaik Karimullah (2024) [140] | Colliding bodies optimization + densely connected CNN (CBO + DenseNet CNN) | LIDC-IDRI | Public | Image histogram equalization | Colliding bodies optimization (CBO) | 70% for training, 10% for validation, and 20% for testing | Better performance compared to neural networks + random forest, ResNet-50, V-Net segmentation, deep pyramidal residual network, neural networks + logistic ML methods |
| Chapala Venkatesh (2022) [141] | Cuckoo search optimization + convolutional neural network + local binary pattern (CSO+CNN+LBP) | CT image | Public | Median filter, thresholding | Cuckoo search optimization | ‒ | Better performance compared to PSO+SVM+LBP, GA+SVM +LBP methods |
| Sugandha Saxena (2024) [142] | Maximum sensitivity neural network (MSNN) model | Hospital dataset | Private | Convolutional network method | Adam optimizer | 70% for training, 30% for testing | Better performance compared to faster R-CNN, faster R-CNN, SC-dynamic R-CNN methods |
| Shanchen Pang (2020) [143] | VGG16-T | Hospital dataset | Cohort | Resizing and normalization methods, filters | Revolving, shifting and reproducing operations | 60% for training, 20% for validation, and 20% for testing | Better performance compared to AlexNet, AlexNet+w, ResNet-34, ResNet-34+w, DenseNet, DenseNet+w methods |
| Sunil Kumar (2025) [144] | Residual U-Net + ResNet-101 | NSCLC-Radiomics | Public | Contrast limited adaptive histogram equalization (CLAHE) | Dimensionality reduction technique, rotating, flipping, zooming, and shifting methods | 80% for training, 10% for validation, and 10% for testing | Better performance compared to residual U-Net + DenseNet-121, U-Net with DenseNet + ResNet-101, U-Net with DenseNet + DenseNet-121 methods |
| N Raghapriya (2024) [145] | Convolutional neural network- long short-term memory (CNN- LSTM) model | Kaggle | Public | Wiener filter, blurring filter | Global particle swarm optimizer (GPSO) | 70% for training, 30% for testing | Better performance compared to PSO, fuzzy C means methods |
| Saurabh Singh Raghuvanshi (2024) [146] | Particle swarm optimization and bayesian optimization (PSbBO-Net) | LC25000 | Public | Min-max technique | Swarm Optimization and Bayesian Optimization (PSbBO), random noise, translation shift, cropping and padding methods | 70% for training, 15% for validation, and 15% for testing | Better performance compared to LeNet (RMSprop), LeNet (ADAM), AlexNet (SGD), AlexNet (SGD-Drop), VGG-16, BoVW methods |
| Yifan Wang (2024) [147] | Radiomics-based reinforcement learning (RRL) model | LDCT scans | Cohort | ‒ | ‒ | 70% for training, 30% for testing | ‒ |
| Lihaoyun Huang (2025) [148] | Generative artificial intelligence (Gen-AI) models | Cancer genome atlas (TCGA) | Cohort | ‒ | ‒ | ‒ | Better performance compared to existing models |
| Muayed S AL Huseiny (2021) [149] | Transfer learning with GoogLeNet | IQ-OTH/NCCD | Public | Gabor filter | ‒ | ‒ | Better performance compared to SVM, MIP-CNN, GoogLeNet, TumorNet methods |
| Laxmikant Tiwari (2021) [150] | Target based weighted Elman DL neural network (TWEDLN) model | LIDC-IDRI | Public | Modified clip limit-based contrasts limited adaptive histograms equalization (MC-CLAHE), Gaussian, median, and Wiener filters | Optimizer | ‒ | Better performance compared to ANFIS, ANN, SVM, KNN, NB methods |
| Qingji Tian (2021) [151] | Enhanced capsule networks- converged search and rescue (ECN-CSAR) model | Lung CT-diagnosis | Public | Resizing and normalization methods | Basic search and rescue (SAR) optimization algorithm, Adam optimizer | ‒ | Better performance compared to ResNet, KE-CNN, CNN methods |
| Malayil Shanid (2020) [152] | SALP-elephant herding optimization algorithm-based deep belief network (SEOA-DBN) model | LIDC-IDRI | Public | Preprocessing method | SALP-elephant herding optimization algorithm | ‒ | Better performance compared to MSFCM clustering, DFCNet, DBN, hybrid geometric active contour methods |
| Muhammad Asim Saleem (2023) [153] | Sooty tern optimization (SHOA)-optimized DNN model | LIDC-IDRI | Public | Normalization method | Sooty tern optimization algorithm (SHOA) | 70% for training, 30% for testing | Better performance compared to Fuse-TDD, MCCNN, FDG-PET, MV-KBC, ODNN-LDA, DPM-DNN methods |
| Rakesh Sankaran (2024) [154] | Probabilistic bilateral convolutional neural networks (PB-CNN) models | LUNA16 | Public | Bidirectional filter | ‒ | ‒ | Better performance compared to KNN, DT, ANN methods |
| Yu Ding (2023) [155] | Artificial intelligence diagnosis model | Chest CT | Private | ‒ | ‒ | 50% for training, 50% for validation | Better performance compared to 7-autoantibody (7-AAB) panel, AI, carcinoembryonic antigen (CEA) |
| Rapeepat Klangbunrueang (2024) [156] | VGG16 model | IQ-OTH/NCCD | Public | Resizing and normalization methods | Rotation, scaling, flipping methods, Adam optimizer | 70% for training, 20% for validation, and 10% for testing | Better performance compared to mobileNetV2, ResNet50, inceptionV3 methods |
| Venkata Tulasiramu Ponnada (2019) [157] | Edge AI system | LIDC-IDRI | Public | ‒ | Adam optimizer | 50% for training, 50% for testing | - |
| Colin Jacobs (2021) [158] | Deep learning algorithms | Kaggle Data Science Bowl 2017 (DSB2017) | Public | ‒ | ‒ | ‒ | Radiologist performance |
| Nasr Y Gharaibeh (2024) [159] | Convolutional neural network-probabilistic neural network (SCNN-PNN) | LUNA 16 | Public | Butterworth high-pass filter (BHPF) | Adam optimizer, Lung cancer data augmented ensemble (LCDAE) architecture | ‒ | Better performance compared to DenseNet201, CNN + SVM methods |
| Mohammad Javad Shafiee (2017) [160] | Evolved deep radiomic sequencer (EDRS) | LIDC-IDRI | Public | Filters | ‒ | ‒ | Better performance compared to DARS, CNN-MIL, SNRS, DRS methods |
| Wang Du (2022) [161] | Artificial intelligence (AI) model | Chest CT image | Cohort | ‒ | ‒ | ‒ | Better performance compared to multidisciplinary teamwork (MDT), Physician performance |
| Imran Nazir (2023) [162] | Machine learning (ML) model | LIDC-IDRI | Public | Resizing and normalization methods | Optimization algorithms | 80% for training, 20% for testing | Better performance compared to gradient-weighted class activation, deep learning, deep convolutional neural network, 3D skeletonization feature based computer-aided detection system, deep reinforcement learning method |
| Mahir Afser Pavel (2024) [163] | Knowledge distillation approach with teaching assistant | NSCLC-Radiomics | Public | Resizing method | Cropping method | 40% for training, 30% for validation, and 30% for testing | Better performance compared to EfficientNetB7, CNN, VCNet, ViT, Swin Transformer, KD ConvNeXt, Enhanced Swin Transformer, MAED, Multi-scale MobileViT methods |
| S Lalitha (2021) [164] | Machine learning algorithm | Laboratory dataset | Private | Adaptive histogram equalization (AHE) technique | ‒ | ‒ | Better performance compared to LESH+CC, Mumford-Shah, Apriori +SVM methods |
| Wafaa Alakwaa (2017) [165] | 3D convolutional neural network (3D-CNN) | DSB2017, LUNA16 | Public | Down sampling, zero-centering, thresholding, and normalization methods | Adam optimizer | 70% for training, 30% for testing | ‒ |
| P Mohamed Shakeel (2019) [166] | Deep learning with instantaneously trained neural networks (DITNN) | Cancer imaging archive (CIA) | Public | Weighted mean histogram equalization method, improved profuse clustering technique (IPCT) | ‒ | 60% for training, 40% for testing | Better performance compared to radial neural networks (RBNN), convolution neural networks (CNN), Hopfield neural networks (HNN), learning vector quantization (LVQ) methods |
| Suren Makaju (2018) [167] | Computer aided diagnosis (CAD) model | LIDC-IDRI | Public | Median filter, Gaussian filter | ‒ | ‒ | Better performance compared to current best model |
| Annemneedi Lakshmanarao (2025) [168] | Machine learning (ML) algorithms | Kaggle | Public | Resizing method | Adam optimizer | 85% for training, 15% for testing | ‒ |
| Chamak Saha (2025) [169] | Lung-AttNet | Kaggle | Public | CLAHE technique, resizing method | Adam and Nadam optimizers | ‒ | Better performance compared to xception, LeNet, VGG16, inception ResNet V2, EffiecientNet B2 methods |
| Kubilay Muhammed Sünnetci (2022) [170] | Probabilistic majority voting and optimization techniques | Chest CT | Public | ‒ | Optimizable support vector machine, optimizable k-nearest neighbor (KNN) | 80% for training, 20% for validation | Better performance compared to volumetric image + newly developed algorithm, deep learning + ensemble, deep feature extraction, stacked generalization principle + sparse autoencoder methods |
| Zaib un Nisa (2023) [171] | Segmented 3D tensors and support vector machines | LUNA 16 | Public | Filters | ‒ | ‒ | Better performance compared to transfer learning VGG16, VGG19, Deep3DScan, FractalNet + CNN, Ensemble Learning, Squeeze Net + Resnet, LungNet-SVM methods |
| Talip Çay (2025) [172] | Convolutional neural networks (CNN) model | Lung CT images | Public | Histogram equalization technique, Otsu’s method, Wiener filter, Canny edge filter | Generative adversarial network (GAN) | 70% for training, 30% for testing | Better performance compared to ResNet50, AlexNet, InceptionV3, VGG19 methods |
| Rabbia Mahum (2023) [173] | Lung-RetinaNet | LIDC-IDRI | Public | Batch normalization (BN) method | Adam optimizer | 75% for training, 25% for testing | Better performance compared to DenseNet201, VGG19, EffiecientNet 82, ResNet101, MobileNetV2 methods |
| S Vishwa Kiran (2022) [174] | Machine learning (ML) with data science-enabled lung cancer diagnosis and classification (MLDS-LCDC) | Chest CT image | Public | Gaussian filter | Sunflower optimization method | 70% for training, 30% for testing | Better performance compared to ODNN, MLP, RBF, ANN, KNN methods |
| Ganashree TS (2025) [175] | CNN model | CT scans | Public | Resizing and normalization methods, filters | ‒ | ‒ | ‒ |
| Zhanlin Ji (2023) [176] | ELCT-YOLO model | CT images | Public | Normalization method | Stochastic gradient descent (SGD) optimizer | 60% for training, 20% for validation, and 20% for testing | Better performance compared to YOLOv3, YOLOv5, YOLOv7-tiny, YOLOv8, SSD, Faster R-CNN methods |
| B Karthikeyan (2024) [177] | Optimal transfer learning method for lung cancer detection and classification (MFFOTL-LCDC) | VIA/I-ELCAP | Public | Normalization method | Remora optimization algorithm (ROA) | 70% for training, 30% for testing, 80% for training, 20% for testing | Better performance compared to ODNN, KNN, DNN, YOLODLN, DBNLND, AGFLCCDGM methods |
| Furkan Berk Seyrek (2024) [178] | Deep learning model | LIDC-IDRI | Public | Sobel filter, resizing method | Adam optimizer | 80% for training, 20% for testing | Better performance compared to AlexNet, ResNet-152V2, VGG-19 methods |
| A Angel Mary (2024) [179] | Block chain and deep learning with dynamic pattern features | LIDC-IDRI | Public | Contrast limited adaptive histogram equalization (CLAHE) | Badger optimization algorithm (HBA) | ‒ | Better performance compared to CNN, ResNet, deep residual network, Inception V3, Improved FasterR-CNN, ProCAN, ASAIL CNN methods |
| C Venkatesh (2020) [180] | Bio-inspired algorithm | CT images of lung cancer | Public | Median filter, Otsu thresholding | Particle swarm optimization |  | Better performance compared to conventional systems |
| Xiaofeng Lin (2021) [181] | Clinical image radiomics deep learning (CIRDL) model | Institution dataset | Cohort | Resizing and normalization methods | Minimum Redundancy Maximum Relevance (mRMR) and the eXtreme Gradient Boosting (XGBoost) algorithms | ‒ | Better performance compared to intra-RDL, Peri-RDL, Gross RDL, IDL, CIDL methods |
| Atul Tiwari (2023) [182] | Hybrid recurrent neural network generative adversarial network (RNN-GAN) model | University dataset | Private | Gaussian filter | Generative adversarial network (GAN), Bat and Whale optimization method | 50% for training, 50% for testing | Better performance compared to CNN, FPSOCNN, SVM, SVM +CNN methods |
| Ahmed Elnakib (2020) [183] | Deep learning optimization model | Early lung cancer action program (ELCAP) | Public | Histogram stretching technique, Wiener filter | Genetic algorithm (GA) feature optimization | 75% for training, 25% for testing | Better performance compared to ALEX, VGG16 methods |
| Roopa Chandrika R (2023) [184] | Deep learning model | LIDC-IDRI | Public | ‒ | Hybrid optimal clustering, optimization Harris Hawk algorithm (HHA) | ‒ | Better performance compared to FCM, Region, Growing, OTSU, Optimal, Clustering, IPGA methods |
| Vikul J Pawar (2023) [185] | Modified cat swarm optimization ‒ layer fused conventional neural network (MCSO+LF-CNN) model | LIDC-IDRI | Public | CLAHE method | Modified cat swarm optimization (MCSO) algorithm | 50%‒90% for training, 10%‒50% for testing | Better performance compared to DBN, SVM, CNN, WOA+LF-CNN, MFO+LF-CNN, CSO+LF-CNN methods |
| Wei Fan (2024) [186] | Artificial intelligence model | Hospital dataset | Cohort | ‒ | ‒ | ‒ | Physician performance |
| Benjamin Hunter (2022) [187] | Radiomics-based decision support tool | UK centres | Cohort | Format changer | ‒ | 70% for training, 30% for testing | Radiologist performance |
| Sneha S Nair (2024) [188] | Random walker- artificial neural networks (RW-ANN) | LIDC-IDRI | Public | Anisotropic diffusion filter | ‒ | ‒ | Better performance compared to KNN, NB, SVM, RF, ANN methods |
| Kang Qi (2024) [189] | Lung-PNet | Chest CT | Cohort | Stochastic window normalization, VOI dilation | Cropping method | 70% for training, 30% for testing | Radiologist performance |
| Mian Muhammad Naeem Abid (2021) [190] | Multi-view convolutional recurrent neural network (MV-CRecNet) | LIDC-IDRI, ELCAP | Public | Batch normalization method | ‒ | 80% for training, 20% for testing; 60% for training, 40% for testing | Better performance compared to 2D-CNN, 3D-CNN, SV-CRecNet methods |
| Pavan Kumar Pagadala (2023) [191] | Deep neural networks model | Kaggle | Public | Histogram equalization technique, Sobel filter, thresholding technique | ‒ | ‒ | Better performance compared to CNN Alexnet + SGD, Deep Learning, CNN with Alexnet, ML with FTIR Signals methods |
| Bin Li (2022) [192] | Computer aided diagnosis (CAD) model | CT scan images | Public | ‒ | ‒ | 70% for training, 10% for validation, and 20% for testing | Physician performance |
| A Asuntha (2020) [193] | Fuzzy particle swarm optimization convolution neural network (FPSOCNN) | Hospital dataset | Private | Histogram equalization (HE) technique, adaptive bilateral filter (ABF), median filter, mean filter, bilateral filter | Fuzzy particle swam optimization (FPSO) method | 50%‒90% for training, 10%‒50% for testing | Better performance compared to feature extraction and LDA based classification, evolutionary convolutional neural network, taxonomic diversity index and phylogenetic distance, frequency domain using support vector machine, wavelet feature descriptor and support vector machine methods |
| Abdulqader Faris Abdulqader (2025) [194] | Multi‑objective deep learning model | Institutional database | Public | Histogram equalization, contrast normalization techniques, Gaussian filter | Rotation, horizontal and vertical flipping, Cropping and padding, and scaling techniques, AdamW optimizer | 70% for training, 15% for validation, and 15% for testing | Better performance compared to YOLOv9, YOLOv10, YOLOv11 models |
| Anindita Saha (2024) [195] | Novel transfer learning model (VER-Net) | Kaggle | Public | Resizing method | Shear range, zoom range, rotation range, horizontal flip true, and vertical flip techniques, Adam and RMSprop optimizers | 65% for training, 10% for validation, and 25% for testing | Better performance compared to NasNetLarge, Xception, DenseNet201, MobileNet, ResNet101, EfficientNetB4 methods |
| Han Yang (2022) [196] | Pulmonary nodules artificial intelligence diagnostic system (PNAIDS) model | Hospital dataset | Cohort | ‒ | ‒ | ‒ | Better performance compared to Mayo model |
| Ran Ni (2025) [197] | Early lung cancer diagnosis model | Hospital dataset | Cohort | ‒ | ‒ | ‒ | Radiologist performance |
| Chengping Zhang (2024) [198] | Dense Net-CNN model | CT scan database | Public | Resizing method, contrast enhancement technique | ‒ | ‒ | Better performance compared to traditional CNNs |
| Asghar Ali Shah (2023) [199] | Deep learning ensemble 2D CNN model | LUNA 16 | Public | Resizing method | Rotation method | 80% for training, 20% for validation | Better performance compared to knowledge-based collaborative deep learning, machine learning algorithms XGBoost and RF, ensemble learning method with SVM, GNB, MLP, and NN |
| Sana Alazwari (2024) [200] | Computer-aided diagnosis for lung cancer by utilizing the waterwheel plant algorithm with deep learning (CADLC-WWPADL) | VIA/I-ELCAP | Public | ‒ | ‒ | 70% for training, 30% for testing | Better performance compared to ODNN, KNN, DNN, YOLO-DLN, DBN-LND, AGFLCC-DGM, DBOMDFF-LCC methods |
| Mohamed Hammad (2025) [201] | Explainable AI gradient-weighted class activation mapping (Grad-CAM) model | Kaggle | Public | Batch normalization method | Image Data Augmenter, rotation, translations, and scaling methods, Adam optimizer | ‒ | Better performance compared to modified DenseNet201, ResNet101, VGG19, AlexNet methods |
| Ward Hendrix (2023) [202] | Artificial intelligence model | LIDC-IDRI, hospital | Public, cohort | ‒ | ‒ | ‒ | Radiologist performance |
| Gür Emre Güraksın (2025) [203] | Lung ensemble convolutional neural network (LECNN) model | IQ-OTH/NCCD | Public | Resizing and morphological operations | Adam optimizer | 80% for training, 20% for testing | Better performance compared to GoogleNet, ResNet18, DarkNet19, EfficientNet methods |
| Shaohua Zheng (2021) [204] | Radiology analysis and malignancy evaluation network (R2MNet) model | LIDC-IDRI | Public | Normalization method | Cropping method | ‒ | Better performance compared to MCNN, TurmorNet, nodule-ResNet, MIT-3DCNN, PN-SAMP, Local-Global Networks methods |

**References**

1. SB P. Performance analysis of lung cancer detection and classification using efficientNet: a deep learning model. Multimed Tools Appl. 2024.

2. AR B, RS VK, SS K. LCD-capsule network for the detection and classification of lung cancer on computed tomography images. Multimedia Tools and Applications. 2023;82(24):37573–92.

3. Aharonu M, Ramasamy LK. An intelligent generative adversarial network multistage lung cancer detection and subtypes classification. International Journal of Machine Learning and Cybernetics. 2024:1–24.

4. Sundaresan AA. Automatic lung cancer detection in low-dose lung CTs using transfer learning. Journal of Advanced Research in Dynamical and Control Systems 2018;10(7):195–201.

5. Alksas A, Shaffie A, Ghazal M, Taher F, Khelifi A, Yaghi M, et al. A novel higher order appearance texture analysis to diagnose lung cancer based on a modified local ternary pattern. Computer Methods and Programs in Biomedicine. 2023;240:107692.

6. Almukhtar FH. Lung cancer diagnosis through CT images using principal component analysis (PCA) and error correcting output codes (ECOC). Journal of Control and Decision. 2024;11(3):472–82.

7. Alshayeji MH, Abed Se. Lung cancer classification and identification framework with automatic nodule segmentation screening using machine learning. Applied Intelligence. 2023;53(16):19724–41.

8. Arumugam SR, Ravichandran B, Baskaran D, Annamalai R. Lung Lobe Segmentation and lung Cancer Detection with Hybrid Optimization Enabled Deep Learning Using CT Images. Journal of Mechanics in Medicine and Biology. 2024.

9. Bagheri Tofighi A, Ahmadi A, Mosadegh H. Improving lung cancer detection via MobileNetV2 and stacked-GRU with explainable AI. International Journal of Information Technology. 2025;17(2):1189–96.

10. Bai Y, Li D, Duan Q, Chen X. Analysis of high-resolution reconstruction of medical images based on deep convolutional neural networks in lung cancer diagnostics. Computer Methods and Programs in Biomedicine. 2022;217:106592.

11. Balaji P, Aluvalu R, Sagar K. Residual attention network based hybrid convolution network model for lung cancer detection. Intelligent Decision Technologies. 2023;17(4):1475–88.

12. Bhattacharjee A, Murugan R, Majumder S, Goel T. Neural network–based computer-aided lung cancer detection. Research on Biomedical Engineering. 2021;37:657–71.

13. Bhattacharjee A, Murugan R, Soni B, Goel T. Ada-gridrf: A fast and automated adaptive boost based grid search optimized random forest ensemble model for lung cancer detection. Physical and Engineering Sciences in Medicine. 2022;45(3):981–94.

14. Bushara A, Kumar RV, Kumar S. An ensemble method for the detection and classification of lung cancer using Computed Tomography images utilizing a capsule network with Visual Geometry Group. Biomedical signal processing and control. 2023;85:104930.

15. Chapala V, Bojja P. IoT based lung cancer detection using machine learning and cuckoo search optimization. International Journal of Pervasive Computing and Communications. 2021;17(5):549–62.

16. Chen L, Zhang Z. The self‐distillation trained multitask dense‐attention network for diagnosing lung cancers based on CT scans. Medical Physics. 2024;51(3):1738–53.

17. Dawood H, Nawaz M, Ilyas MU, Nazir T, Javed A. Attention-guided CenterNet deep learning approach for lung cancer detection. Computers in Biology and Medicine. 2025;186:109613.

18. DEVI S, ATHAVALE V, SURESH A, SHANTHI S, DEEPTI K, VIGNESHWARAN K. TRANSITIONING FROM RADIOGRAPHS TO PRECISION DIAGNOSTICS FOR ATYPICAL CARCINOIDS, ADENOSQUAMOUS CARCINOMA, AND MUCOEPIDERMOID CARCINOMA USING ADVANCED MACHINE LEARNING ALGORITHMS. Journal of the Balkan Tribological Association. 2024;30(3).

19. Dutta AK. Detecting Lung Cancer Using Machine Learning Techniques. Intelligent Automation & Soft Computing. 2022;31(2).

20. Gampala V, Ramya V, Maram B, Pappu SR. Identification of lung cancer using archimedes flow regime optimization enabled deep belief network. Multimedia Tools and Applications. 2024;83(32):78659–88.

21. Gautam N, Basu A, Sarkar R. Lung cancer detection from thoracic CT scans using an ensemble of deep learning models. Neural Computing and Applications. 2024;36(5):2459–77.

22. Heidari A, Javaheri D, Toumaj S, Navimipour NJ, Rezaei M, Unal M. A new lung cancer detection method based on the chest CT images using Federated Learning and blockchain systems. Artificial intelligence in medicine. 2023;141:102572.

23. Helaly HA, Badawy M, El-Gendy EM, Haikal AY. Elcd-nsc2: a novel early lung cancer detection and non-small cell classification framework. Neural Computing and Applications. 2024;36(24):15149–64.

24. Hou S. Auxiliary tumour diagnosis image with deep learning technology. The Journal of Supercomputing. 2022;78(1):578–95.

25. Huidrom R, Chanu YJ, Singh KM. Neuro-evolutional based computer aided detection system on computed tomography for the early detection of lung cancer. Multimedia Tools and Applications. 2022;81(22):32661–73.

26. Jain R, Singh P, Kaur A. An ensemble reinforcement learning-assisted deep learning framework for enhanced lung cancer diagnosis. Swarm and Evolutionary Computation. 2024;91:101767.

27. Kanipriya M, Hemalatha C, Sridevi N, SriVidhya S, Shabu SJ. An improved capuchin search algorithm optimized hybrid CNN-LSTM architecture for malignant lung nodule detection. Biomedical Signal Processing and Control. 2022;78:103973.

28. Kashyap M, Wang X, Panjwani N, Hasan M, Zhang Q, Huang C, et al. Automated Deep Learning-Based Detection and Segmentation of Lung Tumors at CT. Radiology. 2025;314(1):e233029.

29. Kavitha S, Patnala E, Sangaraju HR, Bingu R, Adinarayana S, Dhatterwal JS. An optimized multi-head attention based fused depthwise convolutional model for lung cancer detection. Expert Systems with Applications. 2025:126596.

30. Khalefa SJ. Finite element method and hybrid deep learning approaches: high-accuracy lung cancer detection model. Multiscale and Multidisciplinary Modeling, Experiments and Design. 2024;7(3):3017–29.

31. Nagaraj P. Lung cancer detection and classification using optimized CNN features and Squeeze-Inception-ResNeXt model. Computational Biology and Chemistry. 2025;117:108437.

32. Lanjewar MG, Panchbhai KG, Charanarur P. Lung cancer detection from CT scans using modified DenseNet with feature selection methods and ML classifiers. Expert Systems with Applications. 2023;224:119961.

33. Liang ZJ, Chang S, Gao Y, Cao W, Kuo LR, Pomeroy MJ, et al. Leveraging prior knowledge in machine intelligence to improve lesion diagnosis for early cancer detection. Medical Physics. 2025.

34. Ma L, Wu H, Samundeeswari P. Googlenet-al: A fully automated adaptive model for lung cancer detection. Pattern Recognition. 2024;155:110657.

35. Mammeri S, Amroune M, Haouam M-Y, Bendib I, Corrêa Silva A. Early detection and diagnosis of lung cancer using YOLO v7, and transfer learning. Multimedia Tools and Applications. 2024;83(10):30965–80.

36. Mariappan S, Moses D. Deep learning-based lung cancer detection using CT images. International Journal of Ad Hoc and Ubiquitous Computing. 2024;47(3):143–57.

37. Muthazhagan B, Ravi T, Rajinigirinath D. Sooty-LuCaNet: Sooty tern optimization based deep learning network for lung cancer detection. Journal of Intelligent & Fuzzy Systems. 2023;45(5):8823–36.

38. Nagaraja P, Chennupati SK. Integration of adaptive segmentation with heuristic-aided novel ensemble-based deep learning model for lung cancer detection using CT images. Intelligent Decision Technologies. 2023;17(4):1135–60.

39. Navaneethakrishnan M, Anand MV, Vasavi G, Rani VV. Deep Fuzzy SegNet-based lung nodule segmentation and optimized deep learning for lung cancer detection. Pattern Analysis and Applications. 2023;26(3):1143–59.

40. Ozcelik N, Kıvrak M, Kotan A, Selimoğlu İ. Lung cancer detection based on computed tomography image using convolutional neural networks. Technology and Health Care. 2024;32(3):1795–805.

41. Park Y-J, Choi D, Choi JY, Hyun SH. Performance evaluation of a deep learning system for differential diagnosis of lung cancer with conventional CT and FDG PET/CT using transfer learning and metadata. Clinical Nuclear Medicine. 2021;46(8):635–40.

42. Parvathy C, Jayan J. Automatic Lung Cancer Detection Using Computed Tomography Based on Chan Vese Segmentation and SENET. Optical Memory and Neural Networks. 2024;33(3):339–54.

43. Pethuraj MS, Aboobaider BbM, Salahuddin LB. Analyzing CT images for detecting lung cancer by applying the computational intelligence‐based optimization techniques. Computational Intelligence. 2023;39(6):930–49.

44. Prasad U, Chakravarty S, Mahto G. Lung cancer detection and classification using deep neural network based on hybrid metaheuristic algorithm. Soft Computing. 2024;28(15):8579–602.

45. Quasar SR, Sharma R, Mittal A, Sharma M, Agarwal D, de La Torre Díez I. Ensemble methods for computed tomography scan images to improve lung cancer detection and classification. Multimedia Tools and Applications. 2024;83(17):52867–97.

46. Rathan N, Lokesh S. Enhanced Lung Cancer Diagnosis and Staging With HRNeT: A Deep Learning Approach. International Journal of Imaging Systems and Technology. 2024;34(6):e23193.

47. Rehman A, Harouni M, Zogh F, Saba T, Karimi M, Alamri FS, et al. Detection of lungs tumors in CT scan images using convolutional neural networks. IEEE/ACM Transactions on Computational Biology and Bioinformatics. 2023;21(4):769–77.

48. Sabzalian MH, Kharajinezhadian F, Tajally A, Reihanisaransari R, Alkhazaleh HA, Bokov D. New bidirectional recurrent neural network optimized by improved Ebola search optimization algorithm for lung cancer diagnosis. Biomedical Signal Processing and Control. 2023;84:104965.

49. Shaikh N, Shah P. Automated lung cancer diagnosis using swarm intelligence with deep learning. Computer Methods in Biomechanics and Biomedical Engineering: Imaging & Visualization. 2023;11(6):2363–85.

50. Shakeel PM, Burhanuddin M, Desa MI. Automatic lung cancer detection from CT image using improved deep neural network and ensemble classifier. Neural Computing and Applications. 2022:1–14.

51. Shanid M, Anitha A. Adaptive optimisation driven deep belief networks for lung cancer detection and severity level classification. International Journal of Bio-Inspired Computation. 2021;18(2):114–21.

52. Shen Z, Cao P, Yang J, Zaiane OR. WS-LungNet: A two-stage weakly-supervised lung cancer detection and diagnosis network. Computers in Biology and Medicine. 2023;154:106587.

53. Shi F, Chen B, Cao Q, Wei Y, Zhou Q, Zhang R, et al. Semi-supervised deep transfer learning for benign-malignant diagnosis of pulmonary nodules in chest CT images. IEEE transactions on medical imaging. 2021;41(4):771–81.

54. Siddiqui EA, Chaurasia V, Shandilya M. Detection and classification of lung cancer computed tomography images using a novel improved deep belief network with Gabor filters. Chemometrics and Intelligent Laboratory Systems. 2023;235:104763.

55. Singh A, Dwivedi RK, Rastogi R. Machine Learning Based Framework for Lung Cancer Detection and Image Feature Extraction Using VGG16 with PCA on CT-Scan Images. SN Computer Science. 2024;5(8):1040.

56. Sivasankaran P, Dhanaraj KR. Lung Cancer Detection Using Image Processing Technique Through Deep Learning Algorithm. Revue d'Intelligence Artificielle. 2024;38(1).

57. Sridevi S, RajivKannan A. Development of 3DTDUnet++ with novel function and multi-scale dilated-based deep learning model for lung cancer diagnosis using CT images. Biomedical Signal Processing and Control. 2024;94:106243.

58. Sudha R, Maheswari KU. Automatic lung cancer detection using hybrid particle snake swarm optimization with optimized mask RCNN. Multimedia Tools and Applications. 2024;83(31):76807–31.

59. Sudha R, Maheswari K. Adaptive pelican optimization with optimized mask RCNN for automatic lung cancer detection. Multimedia Tools and Applications. 2025:1–21.

60. Usharani C, Selvapandian A. FedLRes: enhancing lung cancer detection using federated learning with convolution neural network (ResNet50). Neural Computing and Applications. 2025:1–12.

61. Vadala LR, Das M, Madhuri CR, Merugula S. SpiLenet based detection and severity level classification of lung cancer using CT images. Computers and Electrical Engineering. 2025;123:110036.

62. Velmurugan N, Rajeswari R, Naganjaneyulu S, Anupama A. Rat swarm political optimizer based deep learning approach for lung lobe segmentation and lung cancer detection using CT images. Biomedical Signal Processing and Control. 2025;105:107612.

63. Venkatesh C, Bojja P. A dynamic optimization and deep learning technique for detection of lung cancer in CT images and data access through Internet of Things. Wireless Personal Communications. 2022;125(3):2621–46.

64. Wang G, Qiu M, Xing X, Zhou J, Yao H, Li M, et al. Lung cancer scRNA-seq and lipidomics reveal aberrant lipid metabolism for early-stage diagnosis. Science translational medicine. 2022;14(630):eabk2756.

65. Wang M, Yang Z, Zhao R. Advancing lung cancer diagnosis: Combining 3D auto-encoders and attention mechanisms for CT scan analysis. Journal of X-Ray Science and Technology. 2025:08953996241313120.

66. Wani NA, Kumar R, Bedi J. DeepXplainer: An interpretable deep learning based approach for lung cancer detection using explainable artificial intelligence. Computer Methods and Programs in Biomedicine. 2024;243:107879.

67. Xu Y, Wang S, Sun X, Yang Y, Fan J, Jin W, et al. Identification of benign and malignant lung nodules in CT images based on ensemble learning method. Interdisciplinary Sciences: Computational Life Sciences. 2022:1–11.

68. Xu Y, Wang Y, Razmjooy N. Lung cancer diagnosis in CT images based on Alexnet optimized by modified Bowerbird optimization algorithm. Biomedical Signal Processing and Control. 2022;77:103791.

69. Yan C, Razmjooy N. Optimal lung cancer detection based on CNN optimized and improved Snake optimization algorithm. Biomedical Signal Processing and Control. 2023;86:105319.

70. Yang R, Zhang Y, Li W, Li Q, Liu X, Zhang F, et al. Development and external validation of a multimodal integrated feature neural network (MIFNN) for the diagnosis of malignancy in small pulmonary nodules (≤ 10 mm). Biomedical Physics & Engineering Express. 2024;10(4):045008.

71. Zaben SO. Improving lung cancer diagnoses: a machine learning approach for detection and prediction in CT-Scan image analysis. Service Oriented Computing and Applications. 2024:1–16.

72. Zheng Q, Tian X, Yang M, Han S, Elhanashi A, Saponara S, et al. Reconstruction error based implicit regularization method and its engineering application to lung cancer diagnosis. Engineering Applications of Artificial Intelligence. 2025;139:109439.

73. Thanammal K. Lung cancer detection via deep learning-based pyramid network with honey badger algorithm. Measurement: Sensors. 2024;31:100993.

74. Wankhade S, Vigneshwari S. A novel hybrid deep learning method for early detection of lung cancer using neural networks. Healthcare Analytics. 2023;3:100195.

75. Lee J, Chun J, Kim H, Kim JS, Park SY. Development and evaluation of an integrated model based on a deep segmentation network and demography-added radiomics algorithm for segmentation and diagnosis of early lung adenocarcinoma. Computerized Medical Imaging and Graphics. 2023;109:102299.

76. Lee JH, Oh SJ, Kim K, Lim CY, Choi SH, Chung MJ. Improved unsupervised 3D lung lesion detection and localization by fusing global and local features: Validation in 3D low-dose computed tomography. Medical Image Analysis. 2025;103:103559.

77. Masood A, Sheng B, Li P, Hou X, Wei X, Qin J, et al. Computer-assisted decision support system in pulmonary cancer detection and stage classification on CT images. Journal of biomedical informatics. 2018;79:117–28.

78. Zhang Y, Zhang F, Shen C, Qiao G, Wang C, Jin F, et al. Multi-omics model is an effective means to diagnose benign and malignant pulmonary nodules. Clinics. 2025;80:100599.

79. Jayapradha J, Haw S-C, Palanichamy N, Ng K-W, Aneja M, Taiyab A. EMI-LTI: An enhanced integrated model for lung tumor identification using Gabor filter and ROI. MethodsX. 2025;14:103247.

80. Hammad M, ElAffendi M, Asim M, Abd El-Latif AA, Hashiesh R. Automated lung cancer detection using novel genetic TPOT feature optimization with deep learning techniques. Results in Engineering. 2024;24:103448.

81. Pandian R, Vedanarayanan V, Kumar DR, Rajakumar R. Detection and classification of lung cancer using CNN and Google net. Measurement: Sensors. 2022;24:100588.

82. Shatnawi MQ, Abuein Q, Al-Quraan R. Deep learning-based approach to diagnose lung cancer using CT-scan images. Intelligence-Based Medicine. 2025;11:100188.

83. Abe AA, Nyathi M, Okunade A, Pilloy W, Kgole B, Nyakale N. A Robust Deep Learning Algorithm for Lung Cancer Detection from Computed Tomography Images. Intelligence-Based Medicine. 2025:100203.

84. Tsivgoulis M, Papastergiou T, Megalooikonomou V. An improved SqueezeNet model for the diagnosis of lung cancer in CT scans. Machine Learning with Applications. 2022;10:100399.

85. Maleki N, Niaki STA. An intelligent algorithm for lung cancer diagnosis using extracted features from Computerized Tomography images. Healthcare Analytics. 2023;3:100150.

86. Yanagawa M, Niioka H, Kusumoto M, Awai K, Tsubamoto M, Satoh Y, et al. Diagnostic performance for pulmonary adenocarcinoma on CT: comparison of radiologists with and without three-dimensional convolutional neural network. European Radiology. 2021;31:1978–86.

87. Alamgeer M, Mengash HA, Marzouk R, Nour MK, Hilal AM, Motwakel A, et al. Deep Learning Enabled Computer Aided Diagnosis Model for Lung Cancer using Biomedical CT Images. Computers, Materials & Continua. 2022;73(1).

88. Gonçalves L, Novo J, Cunha A, Campilho A. Learning lung nodule malignancy likelihood from radiologist annotations or diagnosis data. Journal of Medical and Biological Engineering. 2018;38(3):424–42.

89. Toğaçar M, Ergen B, Cömert Z. Detection of lung cancer on chest CT images using minimum redundancy maximum relevance feature selection method with convolutional neural networks. Biocybernetics and Biomedical Engineering. 2020;40(1):23–39.

90. Yang K, Liu J, Tang W, Zhang H, Zhang R, Gu J, et al. Identification of benign and malignant pulmonary nodules on chest CT using improved 3D U-Net deep learning framework. European journal of radiology. 2020;129:109013.

91. Abe A, Nyathi M, Okunade A. Lung cancer diagnosis from computed tomography scans using convolutional neural network architecture with Mavage pooling technique. AIMS Medical Science. 2025;12(1):13–27.

92. Bhukya S, Ganagoni V, Nangunoori SS, Enapothula ST. A Deep Learning Framework Using Enhanced Convolutional Neural Network for Detection of Lung Cancer from CT Images. International Research Journal of Multidisciplinary Technovation. 2025;7(1):138–50.

93. JENITA SUBASH DKS. OCHOA-RNN: OPPOSITIONAL BASED CHIMP OPTIMIZATION ALGORITHM (OCHOA) AND RECURRENT

NEURAL NETWORK (RNN) HYBRID CLASSIFIER MODEL FOR LUNG CANCER DIAGNOSIS. Journal of Theoretical and Applied Information Technology. 2023;101(6):2442–58.

94. Taher KF, Ali AM, Hadi GM. MASK-RCNN on the diagnoses of lung cancer in Kurdistan Region of Iraq. Zanco Journal of Pure and Applied Sciences. 2024;36(1):40–8.

95. Thangavel C, Palanichamy J, Chinnasamy S. An advanced perceptual U-Net segmentation based DDO-SDN classification system for lung pulmonary cancer detection. Imaging. 2023;15(1):12–22.

96. Prasad J, Chakravarty S, Krishna MV. Lung cancer detection using an integration of fuzzy K-means clustering and deep learning techniques for CT lung images. Bulletin of the Polish Academy of Sciences Technical Sciences. 2022:e139006–e.

97. Qadir AM, Abdalla PA, Abd DF. A Hybrid Lung Cancer Model for Diagnosis and Stage Classification from Computed Tomography Images. Iraqi Journal for Electrical & Electronic Engineering. 2024;20(2).

98. Mostafa B, Sakr M, Keshk A. Employing the Capabilities of LSTM and Bi-LSTM for Lung Cancer Detection and Classification. International Journal of Intelligent Engineering & Systems. 2024;17(5).

99. Ozdemir O, Russell RL, Berlin AA. A 3D probabilistic deep learning system for detection and diagnosis of lung cancer using low-dose CT scans. IEEE transactions on medical imaging. 2019;39(5):1419–29.

100. Elloumi N, Seddik H, Chaabane SB, Nadra T. A 3D Processing Technique to Detect Lung Tumor. 2023 Published by (IJACSA). 2023.

101. Pang S, Zhang Y, Ding M, Wang X, Xie X. A deep model for lung cancer type identification by densely connected convolutional networks and adaptive boosting. IEEE Access. 2019;8:4799–805.

102. Chenyang L, Chan S-C. A joint detection and recognition approach to lung cancer diagnosis from CT images with label uncertainty. IEEE Access. 2020;8:228905–21.

103. Bhavani Krishna GMT. Advanced mask region-based convolutional neural network based deep-learning model for lung cancer detection. IAES International Journal of Artificial Intelligence. 2024;14(1):1179–86.

104. Duan S, Cao H, Liu H, Miao L, Wang J, Zhou X, et al. Development of a machine learning-based multimode diagnosis system for lung cancer. Aging (Albany NY). 2020;12(10):9840.

105. Chowdary S, Purushotaman SB. An Improved Archimedes Optimization-aided Multi-scale Deep Learning Segmentation with dilated ensemble CNN classification for detecting lung cancer using CT images. Network: Computation in Neural Systems. 2024:1–39.

106. Jakimovski G, Davcev D. Using double convolution neural network for lung cancer stage detection. Applied Sciences. 2019;9(3):427.

107. Ozdemir B, Aslan E, Pacal I. Attention Enhanced InceptionNeXt Based Hybrid Deep Learning Model for Lung Cancer Detection. IEEE Access. 2025.

108. Hussain Ali Y, Sabu Chooralil V, Balasubramanian K, Manyam RR, Kidambi Raju S, T. Sadiq A, et al. Optimization system based on convolutional neural network and internet of medical things for early diagnosis of lung cancer. Bioengineering. 2023;10(3):320.

109. Raptis S, Ilioudis C, Theodorou K. Uncovering the Diagnostic Power of Radiomic Feature Significance in Automated Lung Cancer Detection: An Integrative Analysis of Texture, Shape, and Intensity Contributions. BioMedInformatics. 2024;4(4):2400–25.

110. Liu A, Wang Z, Yang Y, Wang J, Dai X, Wang L, et al. Preoperative diagnosis of malignant pulmonary nodules in lung cancer screening with a radiomics nomogram. Cancer Communications. 2020;40(1):16–24.

111. Shafi I, Din S, Khan A, Díez IDLT, Casanova RdJP, Pifarre KT, et al. An effective method for lung cancer diagnosis from ct scan using deep learning-based support vector network. Cancers. 2022;14(21):5457.

112. Sollini M, Kirienko M, Gozzi N, Bruno A, Torrisi C, Balzarini L, et al. The Development of an Intelligent Agent to Detect and Non-Invasively Characterize Lung Lesions on CT Scans: Ready for the “Real World”? Cancers. 2023;15(2):357.

113. Alamgeer M, Alruwais N, Alshahrani HM, Mohamed A, Assiri M. Dung beetle optimization with deep feature fusion model for lung cancer detection and classification. Cancers. 2023;15(15):3982.

114. Masood A, Yang P, Sheng B, Li H, Li P, Qin J, et al. Cloud-based automated clinical decision support system for detection and diagnosis of lung cancer in chest CT. IEEE journal of translational engineering in health and medicine. 2019;8:1–13.

115. Zhang L, Zeng B, Liu J, Lin H, Lei P, Fan B, et al. Application Potential of Radiomics based on the Unenhanced CT Image for the Identification of Benign or Malignant Pulmonary Nodules. Current Medical Imaging. 2024;20(1):e15734056246425.

116. Song G, Dai Q, Nie Y, Chen G. Differential diagnosis of benign and malignant pulmonary nodules in ct images based on multitask learning. Current Medical Imaging. 2024;20(1):e15734056252399.

117. Ansari MM, Kumar S, Heyat MBB, Ullah H, Hayat MAB, Sumbul, et al. SVMVGGNet-16: A Novel Machine and Deep Learning Based Approaches for Lung Cancer Detection using Combined SVM and VGGNet-16. Current Medical Imaging. 2025;21(1):e15734056348824.

118. Alzubaidi MA, Otoom M, Jaradat H. Comprehensive and comparative global and local feature extraction framework for lung cancer detection using CT scan images. IEEe Access. 2021;9:158140–54.

119. Zhang X, Wang X, Huang T, Sheng J. Deep Learning-Powered Lung Cancer Diagnosis: Harnessing IoT Medical Data and CT Images. International Journal of Advanced Computer Science & Applications. 2024;15(4).

120. Aydın N, Çelik Ö, Aslan AF, Odabaş A, Dündar E, Şahin MC. Detection of lung cancer on computed tomography using artificial intelligence applications developed by deep learning methods and the contribution of deep learning to the classification of lung carcinoma. Current Medical Imaging Reviews. 2021;17(9):1137–41.

121. Gunjan VK, Singh N, Shaik F, Roy S. Detection of lung cancer in CT scans using grey wolf optimization algorithm and recurrent neural network. Health and Technology. 2022;12(6):1197–210.

122. Sori WJ, Feng J, Godana AW, Liu S, Gelmecha DJ. DFD-Net: lung cancer detection from denoised CT scan image using deep learning. Frontiers of Computer Science. 2021;15:1–13.

123. Cho HS, Hwang EJ, Yi J, Choi B, Park CM. Artificial intelligence system for identification of overlooked lung metastasis in abdominopelvic computed tomography scans of patients with malignancy. Diagnostic and Interventional Radiology. 2025;31(2):102.

124. VR N, Chandra SS V. ExtRanFS: An automated lung cancer malignancy detection system using extremely randomized feature selector. Diagnostics. 2023;13(13):2206.

125. Hsiao C-C, Peng C-H, Wu F-Z, Cheng D-C. Impact of Voxel Normalization on a Machine Learning-Based Method: A Study on Pulmonary Nodule Malignancy Diagnosis Using Low-Dose Computed Tomography (LDCT). Diagnostics. 2023;13(24):3690.

126. Bhatia I, Aarti, Ansarullah SI, Amin F, Alabrah A. Lightweight Advanced Deep Neural Network (DNN) Model for Early-Stage Lung Cancer Detection. Diagnostics. 2024;14(21):2356.

127. Bouamrane A, Derdour M, Bennour A, Elfadil Eisa TA, M. Emara A-H, Al-Sarem M, et al. Toward Robust Lung Cancer Diagnosis: Integrating Multiple CT Datasets, Curriculum Learning, and Explainable AI. Diagnostics. 2024;15(1):1.

128. Rajeev Dixit PK, Shashank Ojha. Implementing Deep Learning Models for Early Detection and Segmentation of Lung Cancer from Medical Imaging. Communications on Applied Nonlinear Analysis. 2025;32(2):545–67.

129. Rana M Mahmoud ME, Mohamed Taha. 3D visualization diagnostics for lung cancer detection. IAES International Journal of Artificial Intelligence. 2024;13(4):4630–41.

130. Yamada D, Kojima F, Otsuka Y, Kawakami K, Koishi N, Oba K, et al. Multimodal modeling with low-dose CT and clinical information for diagnostic artificial intelligence on mediastinal tumors: a preliminary study. BMJ Open Respiratory Research. 2024;11(1):e002249.

131. Kavitha Belegere Chandraiah NKB. An optimal model for detection of lung cancer using convolutional neural network. Indonesian Journal of Electrical Engineering and Computer Science. 2024;34(1):134–43.

132. Nair SS, Devi VM, Bhasi S. Lung Cancer Detection from CT Images: Modified Adaptive Threshold Segmentation with Support Vector Machines and Artificial Neural Network Classifier. Current Medical Imaging. 2024;20(1):e140723218727.

133. Ponnada VT, Srinivasu SN. Efficient CNN for lung cancer detection. Int J Recent Technol Eng. 2019;8(2):3499–505.

134. SU A, PP FR, Abraham A, Stephen D. Deep learning-based BoVW–CRNN model for lung tumor detection in nano-segmented CT images. Electronics. 2022;12(1):14.

135. Wehbe A, Dellepiane S, Minetti I. Enhanced Lung Cancer Detection and TNM Staging Using YOLOv8 and TNMClassifier: An Integrated Deep Learning Approach for CT Imaging. IEEE Access. 2024.

136. Tawfik N, Emara HM, El-Shafai W, Soliman NF, Algarni AD, Abd El-Samie FE. Enhancing Early Detection of Lung Cancer through Advanced Image Processing Techniques and Deep Learning Architectures for CT Scans. Computers, Materials and Continua. 2024;81(1):271–307.

137. Vemula ST, Sreevani M, Rajarajeswari P, Bhargavi K, Tavares JMR, Alankritha S. Deep Learning Techniques for Lung Cancer Recognition. Engineering, Technology & Applied Science Research. 2024;14(4):14916–22.

138. Sayana Sharma MK, Deepak Saini. Lung Cancer Detection using Convolutional Neural Network. International Journal of Engineering and Advanced Technology. 2019;8(6):3256–62.

139. Bhattacharjee A, Rabea S, Bhattacharjee A, Elkaeed EB, Murugan R, Selim HMRM, et al. A multi-class deep learning model for early lung cancer and chronic kidney disease detection using computed tomography images. Frontiers in Oncology. 2023;13:1193746.

140. Karimullah S, Khan M, Shaik F, Alabduallah B, Almjally A. An integrated method for detecting lung cancer via CT scanning via optimization, deep learning, and IoT data transmission. Frontiers in Oncology. 2024;14:1435041.

141. Venkatesh C, Ramana K, Lakkisetty SY, Band SS, Agarwal S, Mosavi A. A neural network and optimization based lung cancer detection system in CT images. Frontiers in public health. 2022;10:769692.

142. Saxena S, Prasad N. Design of novel convolution neural network model for lung cancer detection by using sensitivity maps. Int J Artif Intell ISSN. 2024;2252(8938):3219.

143. Pang S, Meng F, Wang X, Wang J, Song T, Wang X, et al. VGG16-T: a novel deep convolutional neural network with boosting to identify pathological type of lung cancer in early stage by CT images. International Journal of Computational Intelligence Systems. 2020;13(1):771–80.

144. Kumar S. Optimized Lung Cancer Identification in CT Imaging: A Synergistic Deep Learning Approach with Residual U-Net Segmentation and Swin Transformer Feature Extraction. International Journal of Computer Information Systems and Industrial Management Applications. 2025;17:16–.

145. N Raghapriya YK. Lung Cancer Detection Using Integration of Hybrid Segmentation Approach and DL Techniques. SSRG International Journal of Electronics and Communication Engineering. 2024;11(6):148–57.

146. Raghuvanshi SS, Arya K, Patel V. PSbBO-Net: A Hybrid Particle Swarm and Bayesian Optimization-based DenseNet for Lung Cancer Detection using Histopathological and CT Images. International Journal of Electrical and Electronics Research. 2024;12(3):1074–86.

147. Wang Y, Zhou C, Ying L, Lee E, Chan H-P, Chughtai A, et al. Leveraging serial low-dose CT scans in radiomics-based reinforcement learning to improve early diagnosis of lung cancer at baseline screening. Radiology: Cardiothoracic Imaging. 2024;6(3):e230196.

148. Huang L, Lin A, Li H, Wang Q, Shen J, Jiang A, et al. Unveiling large multimodal models in pulmonary CT: A comparative assessment of generative AI performance in lung cancer diagnostics. View. 2025:20250077.

149. Al-Huseiny MS, Sajit AS. Transfer learning with GoogLeNet for detection of lung cancer. Indonesian Journal of Electrical Engineering and computer science. 2021;22(2):1078–86.

150. Tiwari L, Raja R, Awasthi V, Miri R, Sinha G, Alkinani MH, et al. Detection of lung nodule and cancer using novel Mask-3 FCM and TWEDLNN algorithms. Measurement. 2021;172:108882.

151. Tian Q, Wu Y, Ren X, Razmjooy N. A new optimized sequential method for lung tumor diagnosis based on deep learning and converged search and rescue algorithm. Biomedical Signal Processing and Control. 2021;68:102761.

152. Shanid M, Anitha A. Lung cancer detection from CT images using salp-elephant optimization-based deep learning. Biomedical Engineering: Applications, Basis and Communications. 2020;32(01):2050001.

153. Saleem MA, Thien Le N, Asdornwised W, Chaitusaney S, Javeed A, Benjapolakul W. Sooty tern optimization algorithm-based deep learning model for diagnosing NSCLC tumours. Sensors. 2023;23(4):2147.

154. Rakesh Sankaran SS, Lakshay Jeet Singh, Jaspreet Sidhu, Anisha Chaudhary, Jagtej Singh. Deep learning-based computerized diagnosis of lung cancer. Salud, Ciencia y Tecnologia. 2024;4:1–8.

155. Ding Y, Zhang J, Zhuang W, Gao Z, Kuang K, Tian D, et al. Improving the efficiency of identifying malignant pulmonary nodules before surgery via a combination of artificial intelligence CT image recognition and serum autoantibodies. European Radiology. 2023;33(5):3092–102.

156. Klangbunrueang R, Pookduang P, Chansanam W, Lunrasri T, editors. AI-Powered Lung Cancer Detection: Assessing VGG16 and CNN Architectures for CT Scan Image Classification. Informatics; 2025: MDPI.

157. Ponnada VT, Srinivasu SN. Integrated clinician decision supporting system for pneumonia and lung cancer detection. International Journal of Innovative Technology and Exploring Engineering (IJITEE). 2019.

158. Jacobs C, Setio AA, Scholten ET, Gerke PK, Bhattacharya H, M. Hoesein FA, et al. Deep learning for lung cancer detection on screening CT scans: results of a large-scale public competition and an observer study with 11 radiologists. Radiology: Artificial Intelligence. 2021;3(6):e210027.

159. Gharaibeh NY, De Fazio R, Al-Naami B, Al-Hinnawi A-R, Visconti P. Automated Lung Cancer Diagnosis Applying Butterworth Filtering, Bi-Level Feature Extraction, and Sparce Convolutional Neural Network to Luna 16 CT Images. Journal of Imaging. 2024;10(7):168.

160. Shafiee MJ, Chung AG, Khalvati F, Haider MA, Wong A. Discovery radiomics via evolutionary deep radiomic sequencer discovery for pathologically proven lung cancer detection. Journal of medical imaging. 2017;4(4):041305–.

161. Du W, He B, Luo X, Chen M. Diagnostic value of artificial intelligence based on CT image in benign and malignant pulmonary nodules. Journal of Oncology. 2022;2022(1):5818423.

162. Nazir I, Haq Iu, AlQahtani SA, Jadoon MM, Dahshan M. Machine Learning‐Based Lung Cancer Detection Using Multiview Image Registration and Fusion. Journal of Sensors. 2023;2023(1):6683438.

163. Pavel MA, Islam R, Babor SB, Mehadi R, Khan R. Non-small cell lung cancer detection through knowledge distillation approach with teaching assistant. PloS one. 2024;19(11):e0306441.

164. Lalitha S. An automated lung cancer detection system based on machine learning algorithm. Journal of Intelligent & Fuzzy Systems. 2021;40(4):6355–64.

165. Alakwaa W, Nassef M, Badr A. Lung cancer detection and classification with 3D convolutional neural network (3D-CNN). International Journal of Advanced Computer Science and Applications. 2017;8(8).

166. Shakeel PM, Burhanuddin MA, Desa MI. Lung cancer detection from CT image using improved profuse clustering and deep learning instantaneously trained neural networks. Measurement. 2019;145:702–12.

167. Makaju S, Prasad P, Alsadoon A, Singh A, Elchouemi A. Lung cancer detection using CT scan images. Procedia Computer Science. 2018;125:107–14.

168. Annemneedi Lakshmanarao NG, Nagagopiraju Vullam, Mandapati Sridhar, Modalavalasa Krishna Kanth, Uma Maheswari Rayudu. Lung cancer detection using hybrid integration of autoencoder feature extraction and ML techniques. Indonesian Journal of Electrical Engineering and Computer Science. 2025;37(1):416~24.

169. Saha C, Saha S, Rahman MA, Milu MH, Higa H, Rashid MA, et al. Lung-AttNet: An Attention Mechanism based CNN Architecture for Lung Cancer Detection with Federated Learning. IEEE Access. 2025.

170. Sünnetci KM, Alkan A. Lung cancer detection by using probabilistic majority voting and optimization techniques. International Journal of Imaging Systems and Technology. 2022;32(6):2049–65.

171. un Nisa Z, Jaffar A, Bhatti SM, Butt UM. Lung Cancer Detection using Segmented 3D Tensors and Support Vector Machines. International Journal of Advanced Computer Science and Applications. 2023;14(10).

172. ÇAY T. Lung cancer diagnosis with GAN supported deep learning models. Bio-Medical Materials and Engineering. 2025:09592989241308775.

173. Mahum R, Al-Salman AS. Lung-RetinaNet: Lung cancer detection using a RetinaNet with multi-scale feature fusion and context module. IEEE Access. 2023;11:53850–61.

174. Vishwa Kiran S, Kaur I, Thangaraj K, Saveetha V, Kingsy Grace R, Arulkumar N. Machine learning with data science-enabled lung cancer diagnosis and classification using computed tomography images. International Journal of Image and Graphics. 2023;23(03):2240002.

175. Ganashree TS MC, Mithileysh Sathiyanarayanan. Convolutional Neural Networks and AI Technology for Early Detection of Lung Cancer: A Deep Learning Approach. International Research Journal of Multidisciplinary Scope. 2025;6(2):141–51.

176. Ji Z, Zhao J, Liu J, Zeng X, Zhang H, Zhang X, et al. ELCT-YOLO: An efficient one-stage model for automatic lung tumor detection based on CT images. Mathematics. 2023;11(10):2344.

177. Karthikeyan B, Seethalakshmi N, Nandhini V, Vinoth D, Muthusamy P, Bellam K. Multimodal Feature Fusion Using Optimal Transfer Learning Approach for Lung Cancer Detection and Classification on CT Images. Full Length Article. 2024;12(2024):84–4.

178. Seyrek FB, Yiğit H. Diagnosis of lung cancer from computed tomography scans with deep learning methods. Journal of Universal Computer Science. 2024;30(8):1089.

179. Mary AA, Thanammal K. BlockChain and Deep Learning with Dynamic Pattern Features for Lung Cancer Diagnosis. International Journal of Advanced Computer Science & Applications. 2024;15(8).

180. Venkatesh C, Bojja P. Lung cancer detection using bio-inspired algorithm in CT scans and secure data transmission through IoT cloud. International Journal of Advanced Computer Science and Applications. 2020;11(11).

181. Lin X, Jiao H, Pang Z, Chen H, Wu W, Wang X, et al. Lung cancer and granuloma identification using a deep learning model to extract 3-dimensional radiomics features in CT imaging. Clinical Lung Cancer. 2021;22(5):e756–e66.

182. Tiwari A, Hannan SA, Pinnamaneni R, Al-Ansari ARM, El-Ebiary YAB, Prema S, et al. Optimized ensemble of hybrid rnn-gan models for accurate and automated lung tumour detection from ct images. International Journal of Advanced Computer Science and Applications (IJACSA). 2023;14(7).

183. Elnakib A, Amer HM, Abou-Chadi FE. Early lung cancer detection using deep learning optimization. 2020.

184. Anitha V, Behera NR, Krishna PV, NamdeoraoJogekar R, Singh K. Detection of Lung Cancer Using Optimal Hybrid Segmentation and Classification. International Journal of Computer Information Systems and Industrial Management Applications. 2023;15:11–.

185. Pawar VJ, Premchand P, editors. Modified convolutional neural network for lung cancer detection: improved cat swarm-based optimal training. Web Intelligence; 2023: SAGE Publications Sage UK: London, England.

186. Fan W, Liu H, Zhang Y, Chen X, Huang M, Xu B. Diagnostic value of artificial intelligence based on computed tomography (CT) density in benign and malignant pulmonary nodules: a retrospective investigation. PeerJ. 2024;12:e16577.

187. Hunter B, Chen M, Ratnakumar P, Alemu E, Logan A, Linton-Reid K, et al. A radiomics-based decision support tool improves lung cancer diagnosis in combination with the Herder score in large lung nodules. EBioMedicine. 2022;86.

188. Nair SS, Devi VM, Bhasi S. Enhanced lung cancer detection: Integrating improved random walker segmentation with artificial neural network and random forest classifier. Heliyon. 2024;10(7).

189. Qi K, Wang K, Wang X, Zhang Y-D, Lin G, Zhang X, et al. Lung-PNet: an automated deep learning model for the diagnosis of invasive adenocarcinoma in pure ground-glass nodules on chest CT. American Journal of Roentgenology. 2024;222(1):e2329674.

190. Abid MMN, Zia T, Ghafoor M, Windridge D. Multi-view convolutional recurrent neural networks for lung cancer nodule identification. Neurocomputing. 2021;453:299–311.

191. Pagadala PK, Pinapatruni SL, Chanda RK, Katakam S, Peri LSK, Reddy DA. Enhancing Lung Cancer Detection from Lung CT Scan Using Image Processing and Deep Neural Networks. Revue d'Intelligence Artificielle. 2023;37(6):1597.

192. Li B, Gao M, Wang T. The role of visual sensor-based CT imaging for rapid diagnosis of lung cancer markers in athletic patients. REVISTA INTERNACIONAL DE MEDICINA Y CIENCIAS DE LA ACTIVIDAD FISICA Y DEL DEPORTE. 2022;22(88):318–35.

193. Asuntha A, Srinivasan A. Deep learning for lung Cancer detection and classification. Multimedia Tools and Applications. 2020;79(11):7731–62.

194. Abdulqader AF, Abdulameer S, Bishoyi AK, Yadav A, Rekha M, Kundlas M, et al. Multi-objective deep learning for lung cancer detection in CT images: enhancements in tumor classification, localization, and diagnostic efficiency. Discover Oncology. 2025;16(1):529.

195. Saha A, Ganie SM, Pramanik PKD, Yadav RK, Mallik S, Zhao Z. VER-Net: a hybrid transfer learning model for lung cancer detection using CT scan images. BMC medical imaging. 2024;24(1):120.

196. Yang H, Chen H, Zhang G, Li H, Ni R, Yu Y, et al. Diagnostic value of circulating genetically abnormal cells to support computed tomography for benign and malignant pulmonary nodules. BMC cancer. 2022;22(1):382.

197. Ni R, Huang Y, Wang L, Chen H, Zhang G, Yu Y, et al. An early lung cancer diagnosis model for non-smokers incorporating ct imaging analysis and circulating genetically abnormal cells (CACs). BMC cancer. 2025;25(1):124.

198. Zhang C, Aamir M, Guan Y, Al-Razgan M, Awwad EM, Ullah R, et al. Enhancing lung cancer diagnosis with data fusion and mobile edge computing using DenseNet and CNN. Journal of Cloud Computing. 2024;13(1):91.

199. Shah AA, Malik HAM, Muhammad A, Alourani A, Butt ZA. Deep learning ensemble 2D CNN approach towards the detection of lung cancer. Scientific reports. 2023;13(1):2987.

200. Alazwari S, Alsamri J, Asiri MM, Maashi M, Asklany SA, Mahmud A. Computer-aided diagnosis for lung cancer using waterwheel plant algorithm with deep learning. Scientific Reports. 2024;14(1):20647.

201. Hammad M, ElAffendi M, El-Latif AAA, Ateya AA, Ali G, Plawiak P. Explainable AI for lung cancer detection via a custom CNN on CT images. Scientific Reports. 2025;15(1):12707.

202. Hendrix W, Hendrix N, Scholten ET, Mourits M, Trap-de Jong J, Schalekamp S, et al. Deep learning for the detection of benign and malignant pulmonary nodules in non-screening chest CT scans. Communications medicine. 2023;3(1):156.

203. Güraksın GE, Kayadibi I. A Hybrid LECNN Architecture: A Computer-Assisted Early Diagnosis System for Lung Cancer Using CT Images. International Journal of Computational Intelligence Systems. 2025;18(1):35.

204. Zheng S, Shen Z, Pei C, Ding W, Lin H, Zheng J, et al. Interpretative computer-aided lung cancer diagnosis: from radiology analysis to malignancy evaluation. Computer Methods and Programs in Biomedicine. 2021;210:106363.

**Supplementary File**

**Main findings**

The included articles utilized various AI techniques, including CNN (Convolutional Neural Network),[1-3] SVM (Support Vector Machine),[4,5] RF (Random Forest),[2,4] KNN (K-Nearest Neighbor),[6] PM-DL (pattern matching combined with deep learning),[7-9] ANN (Artificial Neural Network),[10-12] DNN (Deep Neural Network),[13-15] CDNs (Convolutional Dense Networks),[16-18] DLS (Deep Learning System),[19,20] LSTM (Long Short-Term Memory),[3,21,22] NNE (Neural Network Ensemble),[23,24] and LDA (Linear Discriminant Analysis).[21] The CNN model appears to be the most commonly used model in the papers. Additionally, hybrid approaches combining machine learning and deep learning also seem to be effective for these models. For example, a hybrid of RF and CNN is observed,[2] and the SVM algorithm from machine learning has been frequently utilized in these studies.

There is a possibility of overlap since some studies might have included the same patients by utilizing the same databases. 60 studies used LIDC-IDRI dataset,[2,3,5,7,9,11,12,16,19,25-81] 21 studies used LUNA 16 dataset [14,19,45,56,57,59,60,64,82-94] an 16 studies used IQ-OTH/NCCD dataset.[17,22,26,95-113]

**References**

1. Arumugam SR, Ravichandran B, Baskaran D, Annamalai R. Lung Lobe Segmentation and lung Cancer Detection with Hybrid Optimization Enabled Deep Learning Using CT Images. Journal of Mechanics in Medicine and Biology 2024.

2. Dutta AK. Detecting Lung Cancer Using Machine Learning Techniques. Intelligent Automation & Soft Computing 2022; 31(2).

3. Kanipriya M, Hemalatha C, Sridevi N, SriVidhya S, Shabu SJ. An improved capuchin search algorithm optimized hybrid CNN-LSTM architecture for malignant lung nodule detection. Biomedical Signal Processing and Control 2022; 78: 103973.

4. Maleki N, Niaki STA. An intelligent algorithm for lung cancer diagnosis using extracted features from Computerized Tomography images. Healthcare Analytics 2023; 3: 100150.

5. Ansari MM, Kumar S, Heyat MBB, et al. SVMVGGNet-16: A Novel Machine and Deep Learning Based Approaches for Lung Cancer Detection using Combined SVM and VGGNet-16. Current Medical Imaging 2025; 21(1): e15734056348824.

6. Sünnetci KM, Alkan A. Lung cancer detection by using probabilistic majority voting and optimization techniques. International Journal of Imaging Systems and Technology 2022; 32(6): 2049-65.

7. Alksas A, Shaffie A, Ghazal M, et al. A novel higher order appearance texture analysis to diagnose lung cancer based on a modified local ternary pattern. Computer Methods and Programs in Biomedicine 2023; 240: 107692.

8. Venkatesh C, Ramana K, Lakkisetty SY, Band SS, Agarwal S, Mosavi A. A neural network and optimization based lung cancer detection system in CT images. Frontiers in public health 2022; 10: 769692.

9. Mary AA, Thanammal K. BlockChain and Deep Learning with Dynamic Pattern Features for Lung Cancer Diagnosis. International Journal of Advanced Computer Science & Applications 2024; 15(8).

10. Duan S, Cao H, Liu H, et al. Development of a machine learning-based multimode diagnosis system for lung cancer. Aging (Albany NY) 2020; 12(10): 9840.

11. Nair SS, Devi VM, Bhasi S. Lung Cancer Detection from CT Images: Modified Adaptive Threshold Segmentation with Support Vector Machines and Artificial Neural Network Classifier. Current Medical Imaging 2024; 20(1): e140723218727.

12. Nair SS, Devi VM, Bhasi S. Enhanced lung cancer detection: Integrating improved random walker segmentation with artificial neural network and random forest classifier. Heliyon 2024; 10(7).

13. Jakimovski G, Davcev D. Using double convolution neural network for lung cancer stage detection. Applied Sciences 2019; 9(3): 427.

14. Bhatia I, Aarti, Ansarullah SI, Amin F, Alabrah A. Lightweight Advanced Deep Neural Network (DNN) Model for Early-Stage Lung Cancer Detection. Diagnostics 2024; 14(21): 2356.

15. Saleem MA, Thien Le N, Asdornwised W, Chaitusaney S, Javeed A, Benjapolakul W. Sooty tern optimization algorithm-based deep learning model for diagnosing NSCLC tumours. Sensors 2023; 23(4): 2147.

16. Gautam N, Basu A, Sarkar R. Lung cancer detection from thoracic CT scans using an ensemble of deep learning models. Neural Computing and Applications 2024; 36(5): 2459-77.

17. Jain R, Singh P, Kaur A. An ensemble reinforcement learning-assisted deep learning framework for enhanced lung cancer diagnosis. Swarm and Evolutionary Computation 2024; 91: 101767.

18. Lanjewar MG, Panchbhai KG, Charanarur P. Lung cancer detection from CT scans using modified DenseNet with feature selection methods and ML classifiers. Expert Systems with Applications 2023; 224: 119961.

19. Ozdemir O, Russell RL, Berlin AA. A 3D probabilistic deep learning system for detection and diagnosis of lung cancer using low-dose CT scans. IEEE transactions on medical imaging 2019; 39(5): 1419-29.

20. Park Y-J, Choi D, Choi JY, Hyun SH. Performance evaluation of a deep learning system for differential diagnosis of lung cancer with conventional CT and FDG PET/CT using transfer learning and metadata. Clinical Nuclear Medicine 2021; 46(8): 635-40.

21. Hammad M, ElAffendi M, Asim M, Abd El-Latif AA, Hashiesh R. Automated lung cancer detection using novel genetic TPOT feature optimization with deep learning techniques. Results in Engineering 2024; 24: 103448.

22. Mostafa B, Sakr M, Keshk A. Employing the capabilities of LSTM and Bi-LSTM for lung cancer detection and classification. International Journal of Intelligent Engineering & Systems 2024; 17(5): 412-23.

23. Chowdary S, Purushotaman SB. An Improved Archimedes Optimization-aided Multi-scale Deep Learning Segmentation with dilated ensemble CNN classification for detecting lung cancer using CT images. Network: Computation in Neural Systems 2024: 1-39.

24. Kashyap M, Wang X, Panjwani N, et al. Automated deep learning–based detection and segmentation of lung tumors at ct imaging. Radiology 2025; 314(1): e233029.

25. Sundaresan AA. Automatic lung cancer detection in low-dose lung CTs using transfer learning. Journal of Advanced Research in Dynamical and Control Systems 2018; 10(7): 195-201.

26. Aharonu M, Ramasamy LK. An intelligent generative adversarial network multistage lung cancer detection and subtypes classification. International Journal of Machine Learning and Cybernetics 2025; 16(5): 3819-42.

27. AR B, RS VK, SS K. LCD-capsule network for the detection and classification of lung cancer on computed tomography images. Multimedia Tools and Applications 2023; 82(24): 37573-92.

28. Alshayeji MH, Abed Se. Lung cancer classification and identification framework with automatic nodule segmentation screening using machine learning. Applied Intelligence 2023; 53(16): 19724-41.

29. Arumugam SR, Ravichandran B, Baskaran D, Annamalai R. LUNG LOBE SEGMENTATION AND LUNG CANCER DETECTION WITH HYBRID OPTIMIZATION-ENABLED DEEP LEARNING USING CT IMAGES. Journal of Mechanics in Medicine and Biology 2025: 2450047.

30. Bhattacharjee A, Murugan R, Soni B, Goel T. Ada-gridrf: A fast and automated adaptive boost based grid search optimized random forest ensemble model for lung cancer detection. Physical and Engineering Sciences in Medicine 2022; 45(3): 981-94.

31. Bushara A, Kumar RV, Kumar S. An ensemble method for the detection and classification of lung cancer using Computed Tomography images utilizing a capsule network with Visual Geometry Group. Biomedical signal processing and control 2023; 85: 104930.

32. Gampala V, Ramya V, Maram B, Pappu SR. Identification of lung cancer using archimedes flow regime optimization enabled deep belief network. Multimedia Tools and Applications 2024; 83(32): 78659-88.

33. Hou S. Auxiliary tumour diagnosis image with deep learning technology. The Journal of Supercomputing 2022; 78(1): 578-95.

34. Huidrom R, Chanu YJ, Singh KM. Neuro-evolutional based computer aided detection system on computed tomography for the early detection of lung cancer. Multimedia Tools and Applications 2022; 81(22): 32661-73.

35. Khalefa SJ. Finite element method and hybrid deep learning approaches: high-accuracy lung cancer detection model. Multiscale and Multidisciplinary Modeling, Experiments and Design 2024; 7(3): 3017-29.

36. Mammeri S, Amroune M, Haouam M-Y, Bendib I, Corrêa Silva A. Early detection and diagnosis of lung cancer using YOLO v7, and transfer learning. Multimedia Tools and Applications 2024; 83(10): 30965-80.

37. Mariappan S, Moses D. Deep learning-based lung cancer detection using CT images. International Journal of Ad Hoc and Ubiquitous Computing 2024; 47(3): 143-57.

38. Muthazhagan B, Ravi T, Rajinigirinath D. Sooty-LuCaNet: Sooty tern optimization based deep learning network for lung cancer detection. Journal of Intelligent & Fuzzy Systems 2023; 45(5): 8823-36.

39. Navaneethakrishnan M, Anand MV, Vasavi G, Rani VV. Deep Fuzzy SegNet-based lung nodule segmentation and optimized deep learning for lung cancer detection. Pattern Analysis and Applications 2023; 26(3): 1143-59.

40. Prasad U, Chakravarty S, Mahto G. Lung cancer detection and classification using deep neural network based on hybrid metaheuristic algorithm. Soft Computing-A Fusion of Foundations, Methodologies & Applications 2024; 28.

41. Rathan N, Lokesh S. Enhanced Lung Cancer Diagnosis and Staging With HRNeT: A Deep Learning Approach. International Journal of Imaging Systems and Technology 2024; 34(6): e23193.

42. Rehman A, Harouni M, Zogh F, et al. Detection of lungs tumors in CT scan images using convolutional neural networks. IEEE/ACM Transactions on Computational Biology and Bioinformatics 2023; 21(4): 769-77.

43. Shanid M, Anitha A. Adaptive optimisation driven deep belief networks for lung cancer detection and severity level classification. International Journal of Bio-Inspired Computation 2021; 18(2): 114-21.

44. Shen Z, Cao P, Yang J, Zaiane OR. WS-LungNet: A two-stage weakly-supervised lung cancer detection and diagnosis network. Computers in Biology and Medicine 2023; 154: 106587.

45. Siddiqui EA, Chaurasia V, Shandilya M. Detection and classification of lung cancer computed tomography images using a novel improved deep belief network with Gabor filters. Chemometrics and Intelligent Laboratory Systems 2023; 235: 104763.

46. Sivasankaran P, Dhanaraj KR. Lung Cancer Detection Using Image Processing Technique Through Deep Learning Algorithm. Revue d'Intelligence Artificielle 2024; 38(1).

47. Sudha R, Maheswari KU. Automatic lung cancer detection using hybrid particle snake swarm optimization with optimized mask RCNN. Multimedia Tools and Applications 2024; 83(31): 76807-31.

48. Sudha R, Maheswari K. Adaptive pelican optimization with optimized mask RCNN for automatic lung cancer detection. Multimedia Tools and Applications 2025: 1-21.

49. Vadala LR, Das M, Madhuri CR, Merugula S. SpiLenet based detection and severity level classification of lung cancer using CT images. Computers and Electrical Engineering 2025; 123: 110036.

50. Velmurugan N, Rajeswari R, Naganjaneyulu S, Anupama A. Rat swarm political optimizer based deep learning approach for lung lobe segmentation and lung cancer detection using CT images. Biomedical Signal Processing and Control 2025; 105: 107612.

51. Xu Y, Wang S, Sun X, et al. Identification of benign and malignant lung nodules in CT images based on ensemble learning method. Interdisciplinary Sciences: Computational Life Sciences 2022; 14(1): 130-40.

52. Thanammal K. Lung cancer detection via deep learning-based pyramid network with honey badger algorithm. Measurement: Sensors 2024; 31: 100993.

53. Lee JH, Oh SJ, Kim K, Lim CY, Choi SH, Chung MJ. Improved unsupervised 3D lung lesion detection and localization by fusing global and local features: Validation in 3D low-dose computed tomography. Medical Image Analysis 2025; 103: 103559.

54. Gonçalves L, Novo J, Cunha A, Campilho A. Learning lung nodule malignancy likelihood from radiologist annotations or diagnosis data. Journal of Medical and Biological Engineering 2018; 38(3): 424-42.

55. Prasad J, Chakravarty S, Krishna MV. Lung cancer detection using an integration of fuzzy K-means clustering and deep learning techniques for CT lung images. Bulletin of the Polish Academy of Sciences Technical Sciences 2022: e139006-e.

56. Chenyang L, Chan S-C. A joint detection and recognition approach to lung cancer diagnosis from CT images with label uncertainty. IEEE Access 2020; 8: 228905-21.

57. Bhavani K, Thimmaiah G. Advanced mask region-based convolutional neural network based deep-learning model for lung cancer detection. IAES Int J Artif Intel(IJ-AI) 2024.

58. Hussain Ali Y, Sabu Chooralil V, Balasubramanian K, et al. Optimization system based on convolutional neural network and internet of medical things for early diagnosis of lung cancer. Bioengineering 2023; 10(3): 320.

59. Masood A, Yang P, Sheng B, et al. Cloud-based automated clinical decision support system for detection and diagnosis of lung cancer in chest CT. IEEE journal of translational engineering in health and medicine 2019; 8: 1-13.

60. Song G, Dai Q, Nie Y, Chen G. Differential diagnosis of benign and malignant pulmonary nodules in ct images based on multitask learning. Current Medical Imaging 2024; 20(1): e15734056252399.

61. Zhang X, Wang X, Huang T, Sheng J. Deep Learning-Powered Lung Cancer Diagnosis: Harnessing IoT Medical Data and CT Images. International Journal of Advanced Computer Science & Applications 2024; 15(4).

62. Bouamrane A, Derdour M, Bennour A, et al. Toward Robust Lung Cancer Diagnosis: Integrating Multiple CT Datasets, Curriculum Learning, and Explainable AI. Diagnostics 2024; 15(1): 1.

63. Rajeev Dixit PK, Shashank Ojha. Implementing Deep Learning Models for Early Detection and Segmentation of Lung Cancer from Medical Imaging. Communications on Applied Nonlinear Analysis 2025; 32: 545-56.

64. Rana M Mahmoud ME, Mohamed Taha. 3D visualization diagnostics for lung cancer detection. IAES International Journal of Artificial Intelligence 2024; 13: 4630-41.

65. Chandraiah KB, Bhoganna NK. An optimal model for detection of lung cancer using convolutional neural network. Indonesian Journal of Electrical Engineering and Computer Science 2024; 34(1): 134-43.

66. Ponnada VT, Srinivasu SN. Efficient CNN for lung cancer detection. Int J Recent Technol Eng 2019; 8(2): 3499-505.

67. SU A, PP FR, Abraham A, Stephen D. Deep learning-based BoVW–CRNN model for lung tumor detection in nano-segmented CT images. Electronics 2022; 12(1): 14.

68. Karimullah S, Khan M, Shaik F, Alabduallah B, Almjally A. An integrated method for detecting lung cancer via CT scanning via optimization, deep learning, and IoT data transmission. Frontiers in Oncology 2024; 14: 1435041.

69. Tiwari L, Raja R, Awasthi V, et al. Detection of lung nodule and cancer using novel Mask-3 FCM and TWEDLNN algorithms. Measurement 2021; 172: 108882.

70. Shanid M, Anitha A. Lung cancer detection from CT images using salp-elephant optimization-based deep learning. Biomedical Engineering: Applications, Basis and Communications 2020; 32(01): 2050001.

71. Ponnada VT, Srinivasu SN. Integrated clinician decision supporting system for pneumonia and lung cancer detection. International Journal of Innovative Technology and Exploring Engineering (IJITEE) 2019.

72. Shafiee MJ, Chung AG, Khalvati F, Haider MA, Wong A. Discovery radiomics via evolutionary deep radiomic sequencer discovery for pathologically proven lung cancer detection. Journal of medical imaging 2017; 4(4): 041305-.

73. Nazir I, Haq Iu, AlQahtani SA, Jadoon MM, Dahshan M. Machine Learning‐Based Lung Cancer Detection Using Multiview Image Registration and Fusion. Journal of Sensors 2023; 2023(1): 6683438.

74. Makaju S, Prasad P, Alsadoon A, Singh A, Elchouemi A. Lung cancer detection using CT scan images. Procedia Computer Science 2018; 125: 107-14.

75. Mahum R, Al-Salman AS. Lung-RetinaNet: Lung cancer detection using a RetinaNet with multi-scale feature fusion and context module. IEEE Access 2023; 11: 53850-61.

76. Seyrek FB, Yiğit H. Diagnosis of lung cancer from computed tomography scans with deep learning methods. Journal of Universal Computer Science 2024; 30(8): 1089.

77. Anitha V, Behera NR, Krishna PV, NamdeoraoJogekar R, Singh K. Detection of Lung Cancer Using Optimal Hybrid Segmentation and Classification. International Journal of Computer Information Systems and Industrial Management Applications 2023; 15: 11-.

78. Pawar VJ, Premchand P. Modified convolutional neural network for lung cancer detection: improved cat swarm-based optimal training. Web Intelligence; 2023: SAGE Publications Sage UK: London, England; 2023. p. 37-59.

79. Abid MMN, Zia T, Ghafoor M, Windridge D. Multi-view convolutional recurrent neural networks for lung cancer nodule identification. Neurocomputing 2021; 453: 299-311.

80. Hendrix W, Hendrix N, Scholten ET, et al. Deep learning for the detection of benign and malignant pulmonary nodules in non-screening chest CT scans. Communications medicine 2023; 3(1): 156.

81. Zheng S, Shen Z, Pei C, et al. Interpretative computer-aided lung cancer diagnosis: from radiology analysis to malignancy evaluation. Computer Methods and Programs in Biomedicine 2021; 210: 106363.

82. Dawood H, Nawaz M, Ilyas MU, Nazir T, Javed A. Attention-guided CenterNet deep learning approach for lung cancer detection. Computers in Biology and Medicine 2025; 186: 109613.

83. Heidari A, Javaheri D, Toumaj S, Navimipour NJ, Rezaei M, Unal M. A new lung cancer detection method based on the chest CT images using Federated Learning and blockchain systems. Artificial intelligence in medicine 2023; 141: 102572.

84. Yang R, Zhang Y, Li W, et al. Development and external validation of a multimodal integrated feature neural network (MIFNN) for the diagnosis of malignancy in small pulmonary nodules (≤ 10 mm). Biomedical Physics & Engineering Express 2024; 10(4): 045008.

85. Wankhade S, Vigneshwari S. A novel hybrid deep learning method for early detection of lung cancer using neural networks. Healthcare Analytics 2023; 3: 100195.

86. Tsivgoulis M, Papastergiou T, Megalooikonomou V. An improved SqueezeNet model for the diagnosis of lung cancer in CT scans. Machine Learning with Applications 2022; 10: 100399.

87. Bhukya S, Ganagoni V, Nangunoori SS, Enapothula ST. A Deep Learning Framework Using Enhanced Convolutional Neural Network for Detection of Lung Cancer from CT Images. International Research Journal of Multidisciplinary Technovation 2025; 7(1): 138-50.

88. Shafi I, Din S, Khan A, et al. An effective method for lung cancer diagnosis from ct scan using deep learning-based support vector network. Cancers 2022; 14(21): 5457.

89. Sollini M, Kirienko M, Gozzi N, et al. The Development of an Intelligent Agent to Detect and Non-Invasively Characterize Lung Lesions on CT Scans: Ready for the “Real World”? Cancers 2023; 15(2): 357.

90. Sankaran R, Sen S, Singh LJ, Sidhu J, Chaudhary A, Singh J. Deep learning-based computerized diagnosis of lung cancer. Salud, Ciencia y Tecnologia 2024.

91. Gharaibeh NY, De Fazio R, Al-Naami B, Al-Hinnawi A-R, Visconti P. Automated lung cancer diagnosis applying butterworth filtering, bi-level feature extraction, and sparce convolutional neural network to luna 16 CT images. Journal of Imaging 2024; 10(7): 168.

92. Alakwaa W, Nassef M, Badr A. Lung cancer detection and classification with 3D convolutional neural network (3D-CNN). International Journal of Advanced Computer Science and Applications 2017; 8(8).

93. un Nisa Z, Jaffar A, Bhatti SM, Butt UM. Lung Cancer Detection using Segmented 3D Tensors and Support Vector Machines. International Journal of Advanced Computer Science and Applications 2023; 14(10).

94. Shah AA, Malik HAM, Muhammad A, Alourani A, Butt ZA. Deep learning ensemble 2D CNN approach towards the detection of lung cancer. Scientific reports 2023; 13(1): 2987.

95. Bagheri Tofighi A, Ahmadi A, Mosadegh H. Improving lung cancer detection via MobileNetV2 and stacked-GRU with explainable AI. International Journal of Information Technology 2025; 17(2): 1189-96.

96. Kavitha S, Patnala E, Sangaraju HR, Bingu R, Adinarayana S, Dhatterwal JS. An optimized multi-head attention based fused depthwise convolutional model for lung cancer detection. Expert Systems with Applications 2025; 271: 126596.

97. Ma L, Wu H, Samundeeswari P. GoogLeNet-AL: A fully automated adaptive model for lung cancer detection. Pattern Recognition 2024; 155: 110657.

98. Parvathy C, Jayan J. Automatic Lung Cancer Detection Using Computed Tomography Based on Chan Vese Segmentation and SENET. Optical Memory and Neural Networks 2024; 33(3): 339-54.

99. Sabzalian MH, Kharajinezhadian F, Tajally A, Reihanisaransari R, Alkhazaleh HA, Bokov D. New bidirectional recurrent neural network optimized by improved Ebola search optimization algorithm for lung cancer diagnosis. Biomedical Signal Processing and Control 2023; 84: 104965.

100. Usharani C, Selvapandian A. FedLRes: enhancing lung cancer detection using federated learning with convolution neural network (ResNet50). Neural Computing and Applications 2025; 37(14): 8273-84.

101. Yan C, Razmjooy N. Optimal lung cancer detection based on CNN optimized and improved Snake optimization algorithm. Biomedical Signal Processing and Control 2023; 86: 105319.

102. Jayapradha J, Haw S-C, Palanichamy N, Ng K-W, Aneja M, Taiyab A. EMI-LTI: An enhanced integrated model for lung tumor identification using Gabor filter and ROI. MethodsX 2025; 14: 103247.

103. Shatnawi MQ, Abuein Q, Al-Quraan R. Deep learning-based approach to diagnose lung cancer using CT-scan images. Intelligence-Based Medicine 2025; 11: 100188.

104. Abe AA, Nyathi M, Okunade A, Pilloy W, Kgole B, Nyakale N. A robust deep learning algorithm for lung cancer detection from computed tomography images. Intelligence-Based Medicine 2025; 11: 100203.

105. Abe A, Nyathi M, Okunade A. Lung cancer diagnosis from computed tomography scans using convolutional neural network architecture with Mavage pooling technique. AIMS Medical Science 2025; 12(1): 13-27.

106. JENITA SUBASH KS. OCHOA-RNN: Oppositional based chimp optimization algorithm (OCHOA) and recurrent neural network (RNN) hybrid classifier model for lung cancer diagnosis. Journal of Theoretical and Applied Information Technology 2023; 101(6): 2442-58.

107. Qadir AM, Abdalla PA, Abd DF. A Hybrid Lung Cancer Model for Diagnosis and Stage Classification from Computed Tomography Images. Iraqi Journal for Electrical & Electronic Engineering 2024; 20(2).

108. Ozdemir B, Aslan E, Pacal I. Attention enhanced inceptionnext based hybrid deep learning model for lung cancer detection. IEEE Access 2025.

109. VR N, Chandra SS V. ExtRanFS: An automated lung cancer malignancy detection system using extremely randomized feature selector. Diagnostics 2023; 13(13): 2206.

110. Bhattacharjee A, Rabea S, Bhattacharjee A, et al. A multi-class deep learning model for early lung cancer and chronic kidney disease detection using computed tomography images. Frontiers in Oncology 2023; 13: 1193746.

111. Al-Huseiny MS, Sajit AS. Transfer learning with GoogLeNet for detection of lung cancer. Indonesian Journal of Electrical Engineering and computer science 2021; 22(2): 1078-86.

112. Klangbunrueang R, Pookduang P, Chansanam W, Lunrasri T. AI-Powered Lung Cancer Detection: Assessing VGG16 and CNN Architectures for CT Scan Image Classification. Informatics; 2025: MDPI; 2025. p. 18.

113. Güraksın GE, Kayadibi I. A hybrid LECNN architecture: a computer-assisted early diagnosis system for lung cancer using CT images. International Journal of Computational Intelligence Systems 2025; 18(1): 35.
